# Supplementary material for: Two-photon-absorbing ruthenium complexes enable near infrared light-driven photocatalysis
Source: Nat Commun. 2022 Apr 28;13:2288. doi: 10.1038/s41467-022-29981-3 (PMC9051202; doi:10.1038/s41467-022-29981-3)
Supplement: Supplementary file 1 — Supplementary Information [file 41467_2022_29981_MOESM1_ESM.pdf]

Supplementary Information for

Two-photon-absorbing ruthenium complexes enable near infrared light-driven photocatalysis

Guanqun Han[a], Guodong Li[a], Jie Huang[b], Chuang Han[a], Claudia Turro[b,*], and Yujie Sun[a,*]

[a]Department of Chemistry, University of Cincinnati, Cincinnati, Ohio, USA
[b]Department of Chemistry & Biochemistry, The Ohio State University, Columbus, Ohio, USA

Inventory of Supplementary Information

Supplementary Figures 1-9 are related to the synthesis.
Supplementary Figures 10-25 and Supplementary Tables 1-10 are related to Figure 2 and Table 1.
Supplementary Figures 26-42 are related to Figure 3.
Supplementary Figures 43-49 are related to Figure 4.

Table of contents

**Content**	**Page**
**SUPPLEMENTARY FIGURES**	9
**Supplementary Figure 1.** Synthetic paths for ligands and complexes **1-5**.	9
**Supplementary Figure 2.** ^{1}H NMR (400 MHz, $CDCl_3$, 298 K) (top) and ^{13}C{^{1}H} NMR (101 MHz, $CDCl_3$, 298 K) (bottom) spectra of bpyvp-H in $CDCl_3$.	10
**Supplementary Figure 3.** ^{1}H NMR (400 MHz, $CDCl_3$, 298 K) (top), ^{19}F NMR (376 MHz, $CDCl_3$, 298 K) (middle), and ^{13}C{^{1}H} NMR (101 MHz, $CDCl_3$, 298 K) (bottom) spectra of bpyvp-F in $CDCl_3$.	11
**Supplementary Figure 4.** ^{1}H NMR (400 MHz, $CDCl_3$, 298 K) (top) and ^{13}C{^{1}H} NMR (101 MHz, $CDCl_3$, 298 K) (bottom) spectra of bpyvp-OMe in $CDCl_3$.	13
**Supplementary Figure 5.** ^{1}H NMR (400 MHz, CD_3CN, 298 K) (top) and ^{13}C{^{1}H} NMR (101 MHz, CD_3CN, 298 K) (bottom) spectra of **1** in CD_3CN.	14
**Supplementary Figure 6.** ^{1}H NMR (400 MHz, CD_3CN, 298 K) (top) and ^{13}C{^{1}H} NMR (101 MHz, CD_3CN, 298 K) (bottom) spectra of **2** in CD_3CN.	15
**Supplementary Figure 7.** ^{1}H NMR (400 MHz, CD_3CN, 298 K) (top) and ^{13}C{^{1}H} NMR(101 MHz, CD_3CN, 298 K) (bottom) spectra of **3** in CD_3CN.	16
**Supplementary Figure 8.** ^{1}H NMR (400 MHz, CD_3COCD_3, 298 K) (top), ^{19}F NMR (376 MHz, CD_3CN, 298 K) (middle) and ^{13}C{^{1}H} NMR (101 MHz, CD_3CN, 298 K) (bottom) spectra of **4** in CD_3CN.	17
**Supplementary Figure 9.** ^{1}H NMR (400 MHz, CD_3CN, 298 K) (top) and ^{13}C{^{1}H} NMR (101 MHz, CD_3CN, 298 K) (bottom) spectra of **5** in CD_3CN.	19
**Supplementary Figure 10.** (a) Absorption, (b) emission and excitation spectra of **1** in CH_3CN. *Insets* in (a) are the calculated EDDMs of selected singlet excited states of **1** (red indicates electron decrease and yellow indicates electron increase).	20
**Supplementary Figure 11**. (a) Absorption, (b) emission and excitation spectra of **2** in CH_3CN. *Insets* in (a) are the calculated EDDMs of selected singlet excited states of **2** (red indicates electron decrease and yellow indicates electron increase).	21

Supplementary Figure 12. (a) Absorption, (b) emission and excitation spectra of **3** in CH_3CN. *Insets* in (a) are the calculated EDDMs of selected singlet excited states of **3** (red indicates electron decrease and yellow indicates electron increase). 22

Supplementary Figure 13. (a) Absorption, (b) emission and excitation spectra of **4** in CH_3CN. *Insets* in (a) are the calculated EDDMs of selected singlet excited states of **4** (red indicates electron decrease and yellow indicates electron increase). 23

Supplementary Figure 14. (a) Absorption, (b) emission and excitation spectra of **5** in CH_3CN. *Insets* in (a) are the calculated EDDMs of selected singlet excited states of **5** (red indicates electron decrease and yellow indicates electron increase). 24

Supplementary Figure 15. Electrochemistry measurements of **1** in DMF: (a) cyclic voltammetry, (b) square wave voltammetry, and (c) cyclic voltammetry with different scan rate (v) ranging from 20 to 500 mV/s. Insert is the relationship between an oxidation peak (at 0.77 V vs $Fc^{+/0}$) or a reduction peak (at -1.64 V vs $Fc^{+/0}$) versus $v^{1/2}$. Condition: three electrode system with Pt wire as the counter electrode, glassy carbon as the working electrode and SCE as the reference electrode, 0.1 M $LiClO_4$ in deaerated DMF. Scan rate of the cyclic voltammetry is 100 mV/s. 25

Supplementary Figure 16. Electrochemistry measurements of **2** in DMF: (a) cyclic voltammetry, (b) square wave voltammetry, and (c) cyclic voltammetry with different scan rate (v) ranging from 20 to 500 mV/s. Insert is the relationship between an oxidation peak (at 0.73 V vs $Fc^{+/0}$) or a reduction peak (at -1.63 V vs $Fc^{+/0}$) versus $v^{1/2}$. Condition: three electrode system with Pt wire as the counter electrode, glassy carbon as the working electrode and SCE as the reference electrode, 0.1 M $LiClO_4$ in deaerated DMF. Scan rate of the cyclic voltammetry is 100 mV/s. 26

Supplementary Figure 17. Electrochemistry measurement of **3** in DMF: (a) cyclic voltammetry, (b) square wave voltammetry, and (c) cyclic voltammetry with different scan rate (v) ranging from 20 to 500 mV/s. Insert is the relationship between an oxidation peak (at 0.67 V vs $Fc^{+/0}$) or a reduction peak (at -1.62 V vs $Fc^{+/0}$) versus $v^{1/2}$. Condition: three electrode system with Pt wire as the counter electrode, glassy carbon as the working electrode and SCE as the reference electrode, 0.1 M $LiClO_4$ in deaerated DMF. Scan rate of the cyclic voltammetry is 100 mV/s. 27

Supplementary Figure 18. Electrochemistry measurement of **4** in DMF: (a) cyclic voltammetry, (b) square wave voltammetry, and (c) cyclic voltammetry with different scan rate (v) ranging from 20 to 500 mV/s. Insert is the relationship between an oxidation peak current (at 0.68 V vs $Fc^{+/0}$) or a reduction peak (at -1.62 V vs $Fc^{+/0}$) versus $v^{1/2}$. Condition: three electrode system with Pt wire as the counter electrode, glassy carbon as the working electrode and SCE as the reference 28

electrode, 0.1 M $LiClO_4$ in deaerated DMF. Scan rate of the cyclic voltammetry is 100 mV/s.

Supplementary Figure 19. Electrochemistry measurement of **5** in DMF: (a) cyclic voltammetry, (b) square wave voltammetry, and (c) cyclic voltammetry with different scan rate (v) ranging from 20 to 500 mV/s. Insert is the relationship between an oxidation peak current (at 0.62 V vs $Fc^{+/0}$) or a reduction peak (at -1.67 V vs $Fc^{+/0}$) versus $v^{1/2}$. Condition: three electrode system with Pt wire as the counter electrode, glassy carbon as the working electrode and SCE as the reference electrode, 0.1 M $LiClO_4$ in deaerated DMF. Scan rate of the cyclic voltammetry is 100 mV/s. 29

Supplementary Table 1. Calculated MOs of **1** (Isovalue = 0.04). 30

Supplementary Table 2. Calculated MOs of **2** (Isovalue = 0.04). 32

Supplementary Table 3. Calculated MOs of **3** (Isovalue = 0.04). 34

Supplementary Table 4. Calculated MOs of **4** (Isovalue = 0.04). 36

Supplementary Table 5. Calculated MOs of **5** (Isovalue = 0.04). 38

Supplementary Table 6. Calculated singlet electronic transitions (f > 0.01) of **1** in CH_3CN (Isovalue = 0.04). 40

Supplementary Table 7. Calculated singlet electronic transitions (f > 0.01) of **2** in CH_3CN (Isovalue = 0.04). 44

Supplementary Table 8. Calculated singlet electronic transitions (f > 0.01) of **3** in CH_3CN (Isovalue = 0.04). 49

Supplementary Table 9. Calculated singlet electronic transitions (f > 0.01) of **4** in CH_3CN (Isovalue = 0.04). 54

Supplementary Table 10. Calculated singlet electronic transitions (f > 0.01) of **5** in CH_3CN (Isovalue = 0.04). 59

Cartesian coordinates of optimized **1** 64

Cartesian coordinates of optimized **2** 67

Cartesian coordinates of optimized **3** 70

Cartesian coordinates of optimized **4** 74

Cartesian coordinates of optimized **5** 78

Supplementary Figure 20. Transient absorption spectra of **2** under irradiation of (a) 480 nm and (b) 800 nm. 82

Supplementary Figure 21. Transient absorption spectra of **3** under irradiation of (a) 480 nm and (b) 800 nm. 83

Supplementary Figure 22. Transient absorption spectra of **4** under irradiation of (a) 480 nm and (b) 800 nm. 84

Supplementary Figure 23. Emission decay of **3** in deaerated (top) and aerated (bottom) CH_3CN. Insets show the intensity plotted on a log scale (excited at 480 nm and decay trace taken at 670 nm). 85

Supplementary Figure 24. Emission decay of **4** in deaerated (top) and aerated (bottom) CH_3CN. Insets show the intensity plotted on a log scale (excited at 480 nm and decay trace taken at 670 nm). 86

Supplementary Figure 25. Emission decay of **5** in deaerated (top) and aerated (bottom) CH_3CN. Insets show the intensity plotted on a log scale (excited at 480 nm and decay trace taken at 670 nm). 87

Supplementary Figure 26. ^{1}H NMR (400 MHz, $CDCl_3$, 298 K) spectra of benzyl amine C-N coupling using **1** as the photosensitizer with different irradiation time: (a) 30 min; (b) 1 h; (c) 2 h; (d) 4 h. Condition: 50 mM benzyl amine, 1 mol% **1**, 5 mL CH_3CN, O_2, 740 nm LEDs, room temperature. 88

Supplementary Figure 27. ^{1}H NMR (400 MHz, $CDCl_3$, 298 K) spectra of benzyl amine C-N coupling using **2** as the photosensitizer with different irradiation time: (a) 20 min; (b) 30 min; (c) 40 min; (d) 60 min; (e) 80 min; (f) 100 min. Condition: 50 mM of benzyl amine, 1 mol% **2**, 5 mL CH_3CN, O_2, 740 nm LEDs, room temperature. 89

Supplementary Figure 28. ^{1}H NMR (400 MHz, $CDCl_3$, 298 K) spectra of benzyl amine C-N coupling using **3** as the photosensitizer with different irradiation time: (a) 10 min; (b) 20 min; (c) 30 min; (d) 40 min; (e) 50 min. Condition: 50 mM benzyl amine, 1 mol% **3**, 5 mL CH_3CN, O_2, 740 nm LEDs, room temperature. 91

Supplementary Figure 29. ^{1}H NMR (400 MHz, $CDCl_3$, 298 K) spectra of benzyl amine C-N coupling using **4** as the photosensitizer with different irradiation time: (a) 10 min; (b) 20 min; (c) 40 min; (d) 80 min; (e) 120 min; (f) 160 min. Condition: 50 mM benzyl amine, 1 mol% **4**, 5 mL CH_3CN, O_2, 740 nm LEDs, room temperature. 92

Supplementary Figure 30. ^{1}H NMR (400 MHz, $CDCl_3$, 298 K) spectra of benzyl amine C-N coupling using **5** as the photosensitizer with different irradiation time: (a) 3 min; (b) 10 min; (c) 15 min; (d) 20 min; (e) 30 min. Condition: 50 mM benzyl amine, 1 mol% **5**, 5 mL CH_3CN, O_2, 740 nm LEDs, room temperature. 94

Supplementary Figure 31. ^{1}H NMR (400 MHz, $CDCl_3$, 298 K) spectra of benzyl amine C-N coupling. (a) Standard benzyl amine; (b) Standard condition: 50 mM benzyl amine, 1 mol%, 5 mL CH_3CN, O_2, 740 nm LEDs, room temperature, 30 min; (c) time is 50 min; (d) no photosensitizer; (e) no irradiation; (f) Ar atmosphere; (g) Air atmosphere; (h) photosensitizer is $[Ru(bpy)_3](PF_6)_2$; (i) photosensitizer is **1**; (j) photosensitizer is complex **1** and time is 4 h; (k) photosensitizer is **2**; (l) photosensitizer is **2** and time is 100 min; (m) photosensitizer is **4**; (n) photosensitizer **4** and time is 160 min; (o) photosensitizer is **5**. 95

Supplementary Figure 32. (a) Yield change over time upon irradiation at 730 nm of different power. (b) The obtained yields after 20 min irradiation at 730 nm of different power. Conditions: 50 mM benzyl amine, 1 mol% **5**, 2 mL CH_3CN, O_2, irradiation using 730 nm LED (M730L5) of controlled power at room temperature with a 695 nm long-pass filter. 98

Supplementary Figure 33. (a) Experimental set-up. (b) Yields of the photocatalytic C-N coupling of benzyl amine in different reaction tubes. Photocatalysis condition of each tube: 50 mM benzyl amine, 1 mol% 5, 4 mL CH_3CN, open to air, irradiation at 456 or 740 nm for 6 h at room temperature. 99

Supplementary Figure 34. ^{1}H NMR (400 MHz, $CDCl_3$, 298 K) spectra of the photocatalytic C-N coupling of benzyl amine in different reaction tubes. Photocatalysis condition of each tube: 50 mM benzyl amine, 1 mol% 5, 4 mL CH_3CN, open to air, irradiation at 456 nm for 6 h at room temperature. 100

Supplementary Figure 35. ^{1}H NMR (400 MHz, $CDCl_3$, 298 K) spectra of the photocatalytic C-N coupling of benzyl amine in different reaction tubes. Photocatalysis condition of each tube: 50 mM benzyl amine, 1 mol% 5, 4 mL CH_3CN, open to air, irradiation at 740 nm for 6 h at room temperature. 101

Supplementary Figure 36. ^{1}H NMR (400 MHz, $CDCl_3$, 298 K) spectra of thioanisole sulfoxidation using **5** as the photosensitizer with different irradiation time: (a) standard thioanisole; (b) 20 min; (c) 40 min; (d) 1 h; (e) 2 h; (f) 3 h. Condition: 50 mM benzyl amine, 1 mol% **5**, 4 mL CH_3CN, 1 mL CH_3OH, O_2, 740 nm LEDs, room temperature. 102

Supplementary Figure 37. Yield change over time of thioanisole sulfoxidation. Condition: 50 mM thioanisole, 1 mol% **5**, 4 mL CH_3CN, 1 mL CH_3OH, O_2, 740 nm LEDs, room temperature. 103

Supplementary Figure 38. ^{1}H NMR (400 MHz, $CDCl_3$, 298 K) spectra of anthracene [4+2] Diels-Alder reaction using **5** as the photosensitizer with different irradiation time: (a) standard anthracene; (b) 20 min; (c) 40 min; (d) 1 h; (e) 2 h; (f) 4 h; (g) 6 h. Condition: 10 mM anthracene, 1 mol% **5**, 5 mL CH_3CN, O_2, 740 nm LEDs, room temperature. 104

Supplementary Figure 39. Yield change over time of anthracene [4+2] Diels-Alder reaction. Condition: 10 mM anthracene, 1 mol% **5**, 4 mL CH_3CN, O_2, 740 nm LEDs, room temperature. 105

Supplementary Figure 40. ^{1}H NMR (400 MHz, $CDCl_3$, 298 K) spectra of cyclooctene oxidation using **5** as the photosensitizer with different irradiation time: (a) standard cyclooctene; (b) 1 h; (c) 3 h; (d) 5 h; (e) 7 h; (f) 10 h. Condition: 50 mM cyclooctene, 1 mol% **5**, 5 mL CD_3CN, O_2, 740 nm LEDs, room temperature. 106

Supplementary Figure 41. Yield change over time of anthracene oxidation. Condition: 10 mM anthracene, 1 mol% **5**, 5 mL CH_3CN, O_2, 740 nm LEDs, room temperature. 107

Supplementary Figure 42. ^{1}H NMR (400 MHz, $CDCl_3$, 298 K) spectra of HMF oxidation using **5** as the photosensitizer with different irradiation time: (a) standard HMF; (b) 30 min; (c) 1 h; (d) 2 h; (e) 3 h; (f) 4 h; (g) 5 h. Condition: 50 mM HMF, 1 mol% **5**, 5 mL CH_3CN, O_2, 740 nm LEDs, room temperature. 108

Supplementary Figure 43. ^{1}H NMR (400 MHz, $CDCl_3$, 298 K) spectra before (a) and after (b) dehalogenation of phenacyl bromide using **5** as the photosensitizer. Condition: 50 mM phenacyl bromide, 10 equiv. TEOA, 0.2 mol% **5**, 5 mL CH_3CN, Ar, 740 nm LEDs, room temperature, 8 h, purified via column chromatography (hexane/ethyl acetate = 100/3). 110

Supplementary Figure 44. ^{1}H NMR (400 MHz, $CDCl_3$, 298 K) spectra before (a) and after (b) cyanation of tetrahydroisoquinoline using **5** as the photosensitizer. Condition: 50 mM tetrahydroisoquinoline, 1 mol% **5**, 5 mL CH_3CN, Ar, 740 nm LEDs, room temperature, 24 h, purified via column chromatography (hexane/ethyl acetate = 100/3). 111

Supplementary Figure 45. Proposed photocatalytic cycle of cyanation using **5** as the photosensitizer under 740 nm irradiation. **$[Ru]^{2+}$** represents **5**. 112

Supplementary Figure 46. ^{1}H NMR (400 MHz, $CDCl_3$, 298 K) (a) and $^{13}C\{^1H\}$ NMR (101 MHz, $CDCl_3$, 298 K) (b) spectra after C-Ccoupling 113

between benzaldehyde and allyl acetate using **5** as the photosensitizer and $Ni(OTf)_2$ as a cocatalyst. Condition: 50 mM benzaldehyde, 10 equiv. allyl acetate, 5 mol% **5**, 15 mol% $Ni(OTf)_2$, 20 mol% *o*-phenanthroline, 10 equiv. DIPEA, 2 mL CH_3CN, Ar, 740 nm LEDs, room temperature, 72 h, purified via column chromatography (hexane/ethyl acetate = 10/1).

Supplementary Figure 47. ^1H NMR (400 MHz, $CDCl_3$, 298 K) (a) and $^{13}C\{^1H\}$ NMR (101 MHz, $CDCl_3$, 298 K) (b) spectra after C-C coupling between 4-methoxybenzaldehyde and allyl acetate using **5** as the photosensitizer and $Ni(OTf)_2$ as a cocatalyst. Condition: 50 mM 4-methoxybenzaldehyde, 10 equiv. allyl acetate, 5 mol% **5**, 15 mol% $Ni(OTf)_2$, 20 mol% *o*-phenanthroline, 10 equiv. DIPEA, 2 mL CH_3CN, Ar, 740 nm LEDs, room temperature, 72 h, purified via column chromatography (hexane/ethyl acetate = 10/1). 114

Supplementary Figure 48. ^1H NMR (400 MHz, $CDCl_3$, 298 K) (a) and $^{13}C\{^1H\}$ NMR (101 MHz, $CDCl_3$, 298 K) (b) spectra after C-C coupling between 4-(trifluoromethyl)benzaldehyde and allyl acetate using **5** as the photosensitizer and $Ni(OTf)_2$ as a cocatalyst. Condition: 50 mM 4-(trifluoromethyl)benzaldehyde, 10 equiv. allyl acetate, 5 mol% **5**, 15 mol% $Ni(OTf)_2$, 20 mol% *o*-phenanthroline, 10 equiv. DIPEA, 2 mL CH_3CN, Ar, 740 LEDs nm, room temperature, 72 h, purified via column chromatography (hexane/ethyl acetate = 10/1). 115

Supplementary Figure 49. Proposed photocatalytic cycle of the allylation of aromatic aldehydes using **5** and Ni-cocatalyst under 740 nm irradiation. **$[Ru]^{2+}$** represents **5**. 116

SUPPLEMENTARY FIGURES

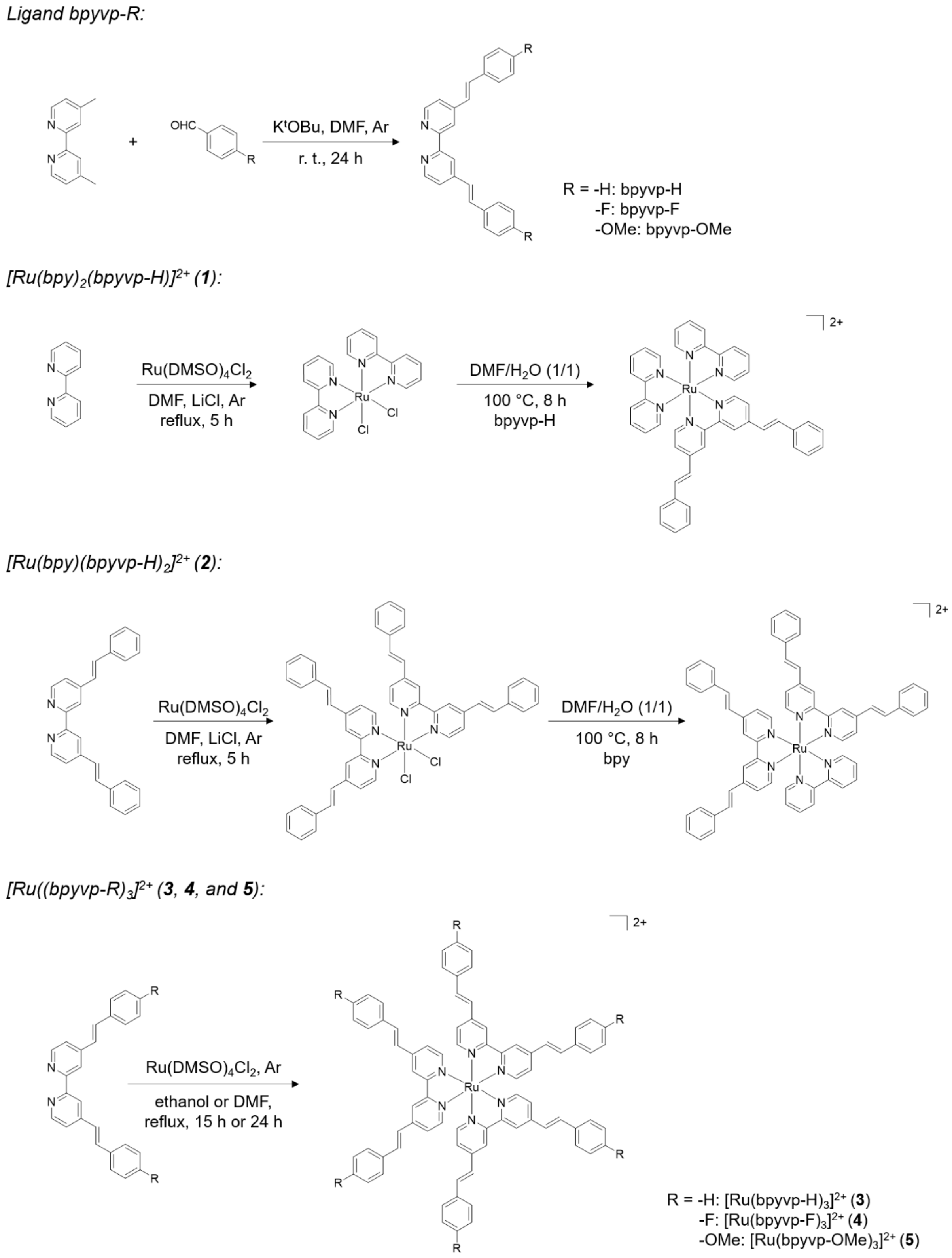

Supplementary Figure 1. Synthetic paths for ligands and complexes **1**-**5**.

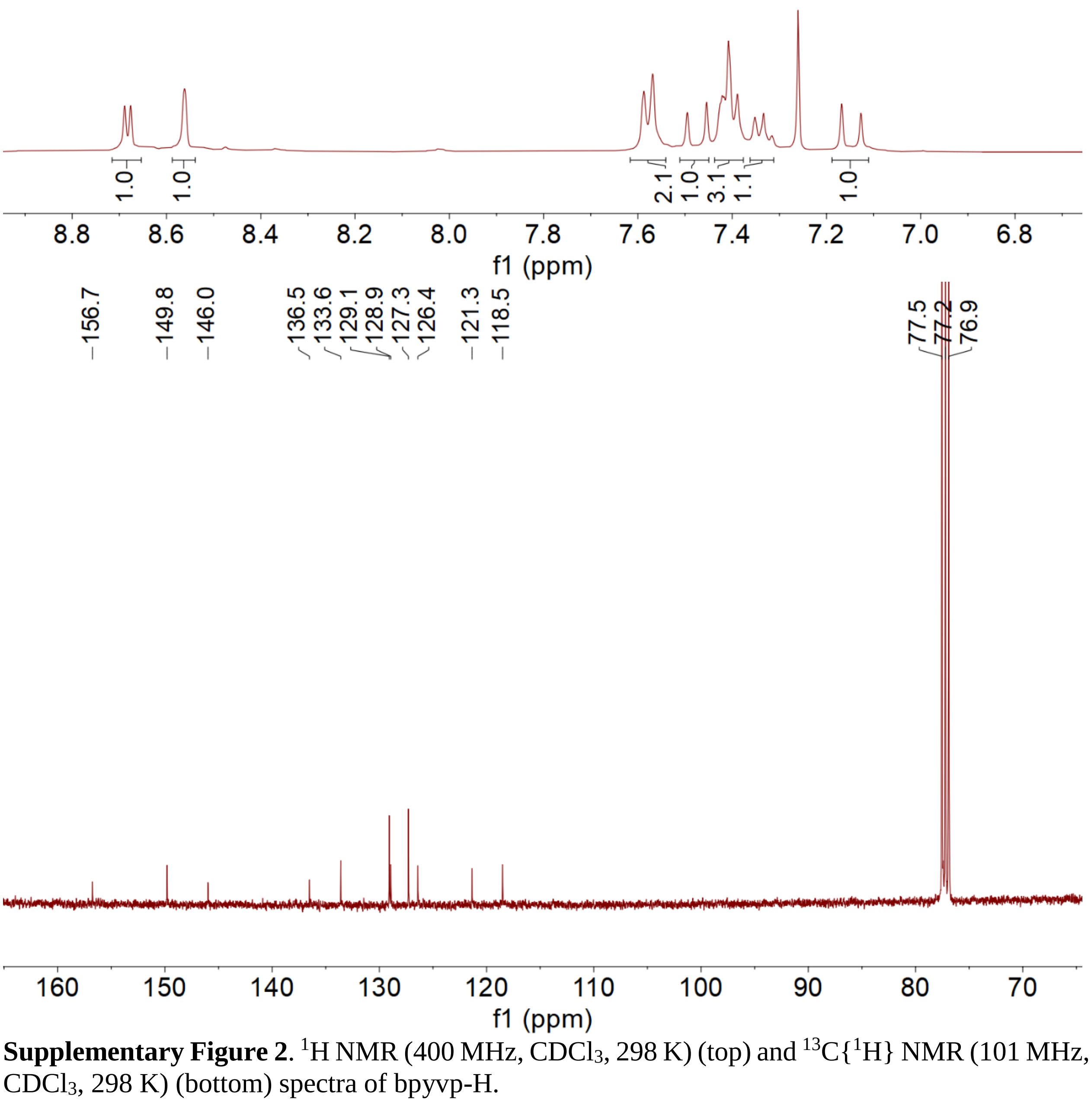

Supplementary Figure 2. ^{1}H NMR (400 MHz, $CDCl_3$, 298 K) (top) and $^{13}C\{^{1}H\}$ NMR (101 MHz, $CDCl_3$, 298 K) (bottom) spectra of bpyvp-H.

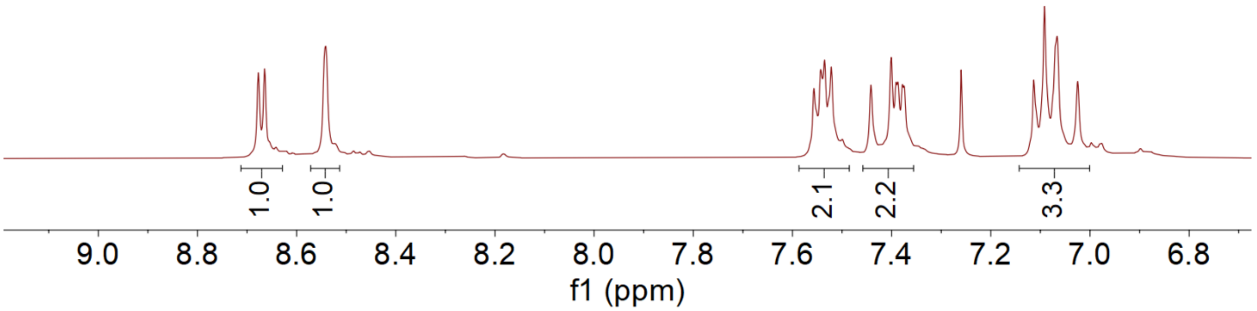
1.0
1.0
2.1
2.2
3.3
9.0 8.8 8.6 8.4 8.2 8.0 7.8 7.6 7.4 7.2 7.0 6.8
f1 (ppm)

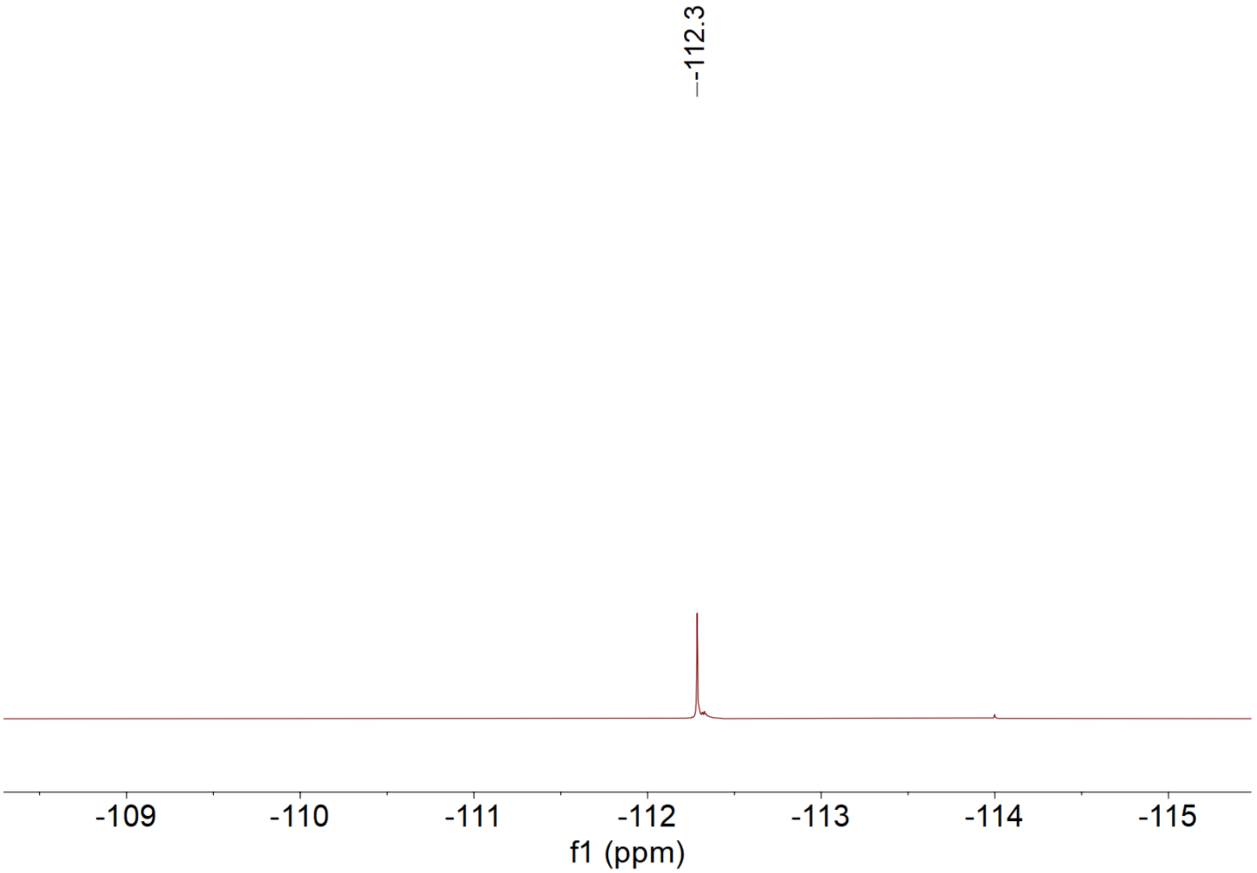
-112.3
-109 -110 -111 -112 -113 -114 -115
f1 (ppm)

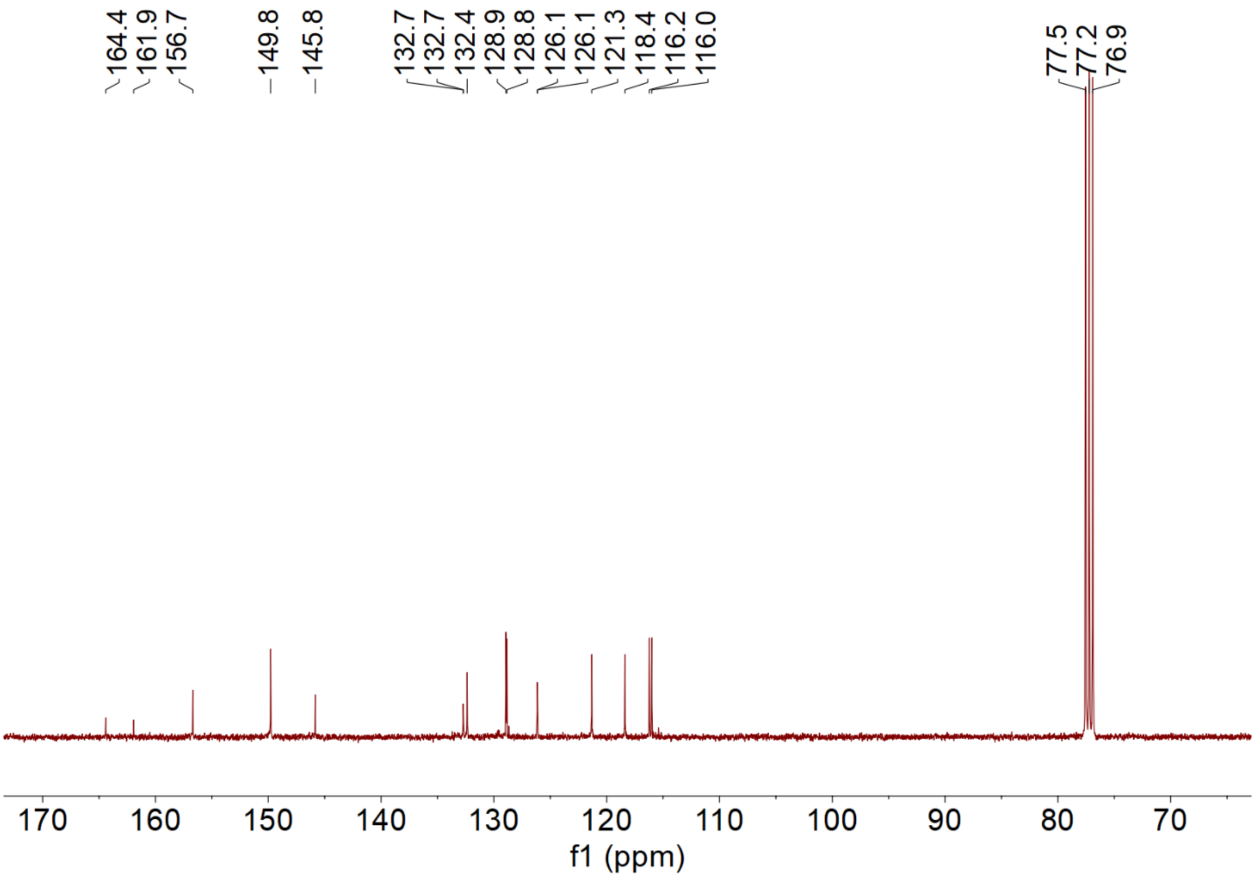

Supplementary Figure 3. ^{1}H NMR (400 MHz, $CDCl_3$, 298 K) (top), ^{19}F NMR (376 MHz, $CDCl_3$, 298 K) (middle), and $^{13}C\{^{1}H\}$ NMR (101 MHz, $CDCl_3$, 298 K) (bottom) spectra of bpyvp-F.

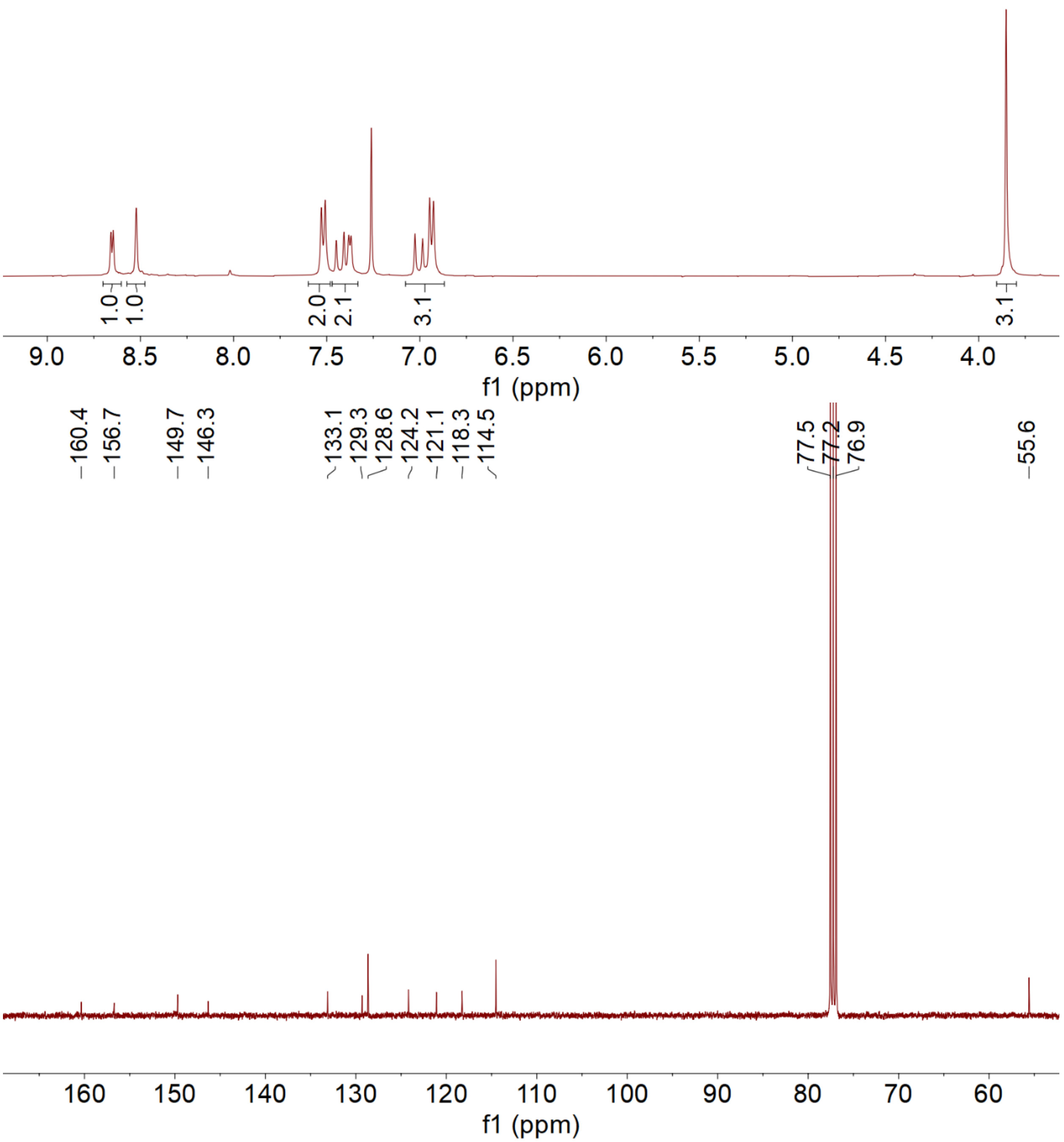

Supplementary Figure 4. ^{1}H NMR (400 MHz, $CDCl_3$, 298 K) (top) and $^{13}C\{^{1}H\}$ NMR (101 MHz, $CDCl_3$, 298 K) (bottom) spectra of bpyvp-OMe.

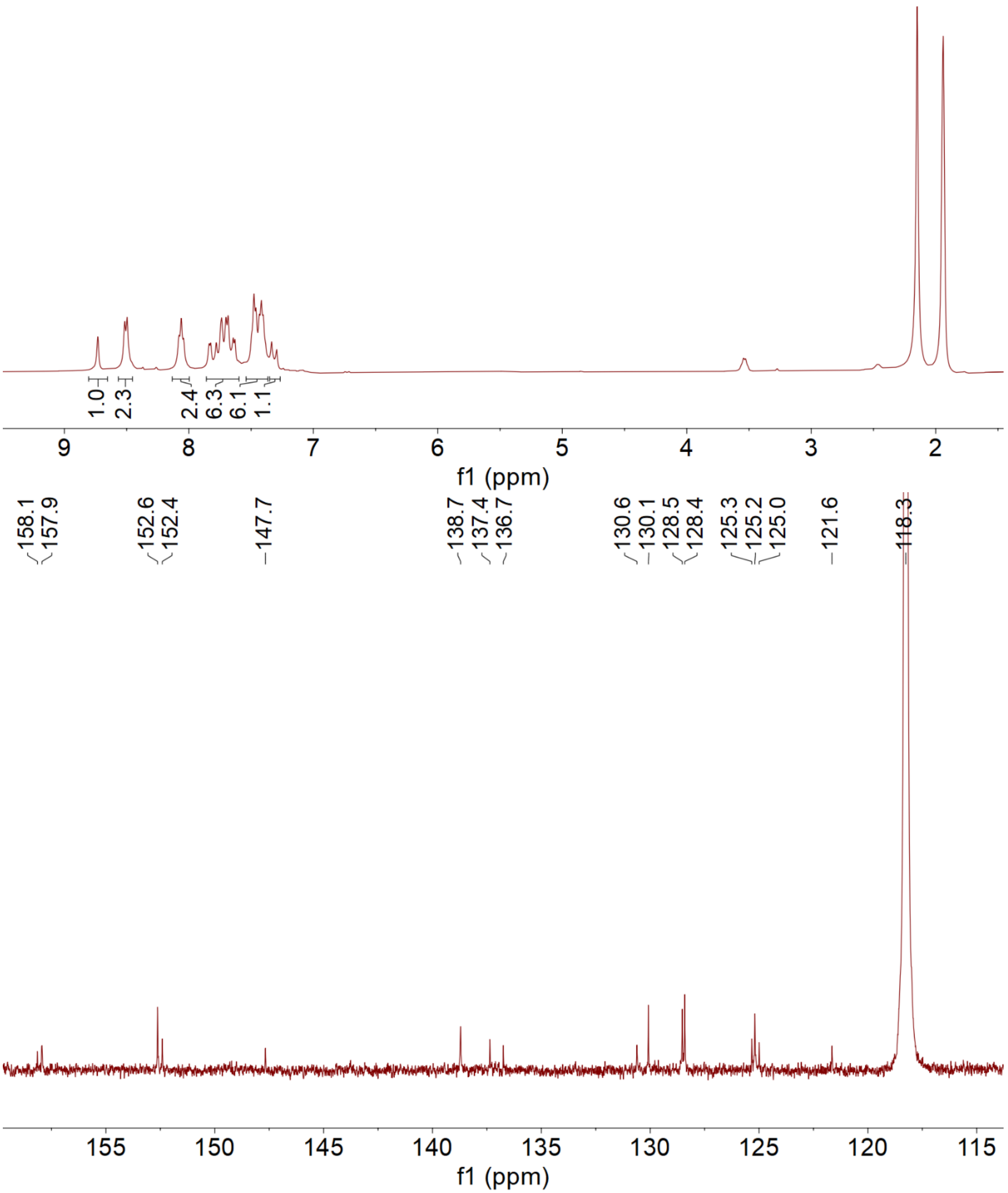

Supplementary Figure 5. ^{1}H NMR (400 MHz, CD_3CN, 298 K) (top) and $^{13}C\{^{1}H\}$ NMR (101 MHz, CD_3CN, 298 K) (bottom) spectra of **1**.

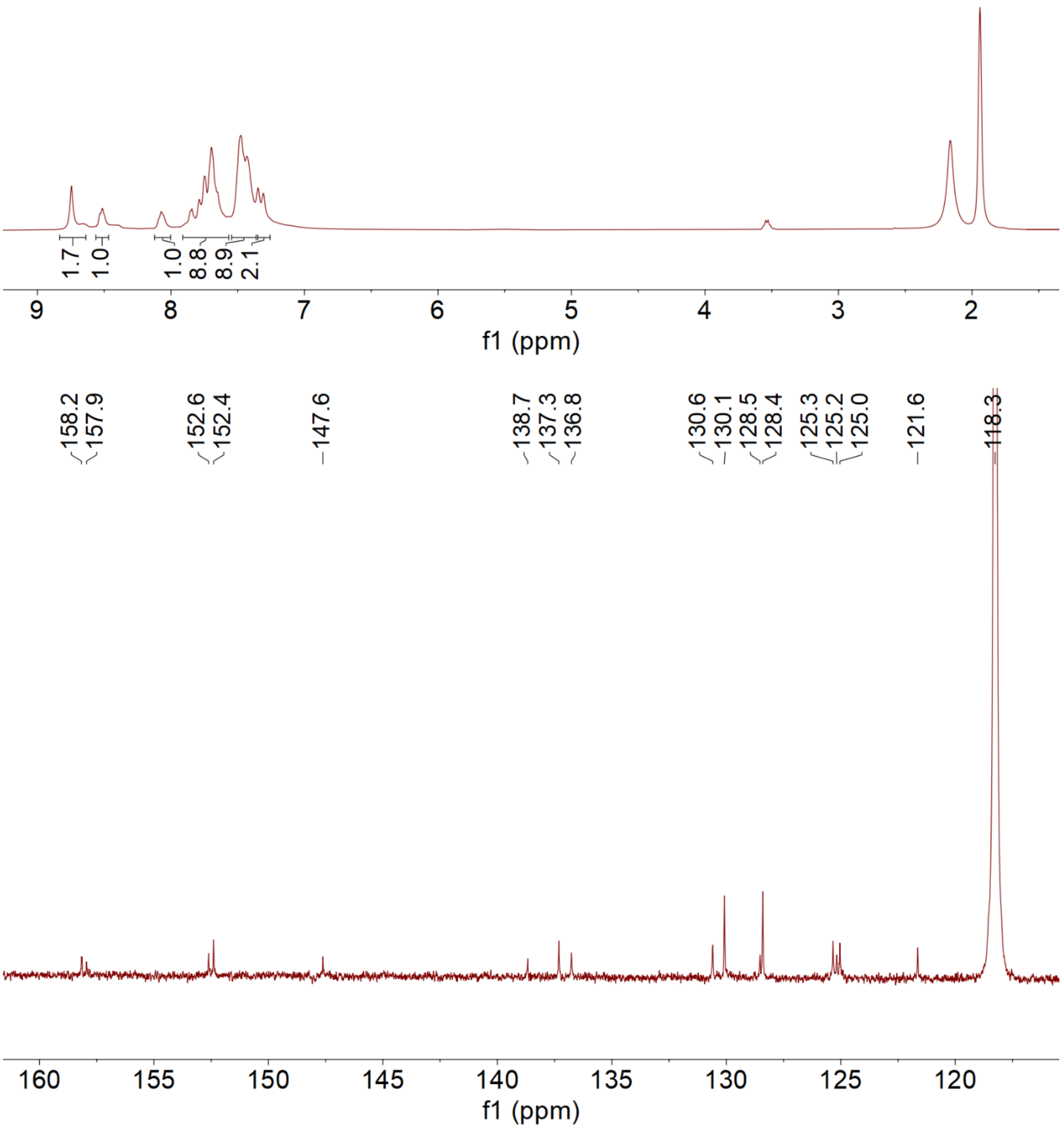

Supplementary Figure 6. ^{1}H NMR (400 MHz, CD_3CN, 298 K) (top) and $^{13}C\{^1H\}$ NMR (101 MHz, CD_3CN, 298 K) (bottom) spectra of **2**.

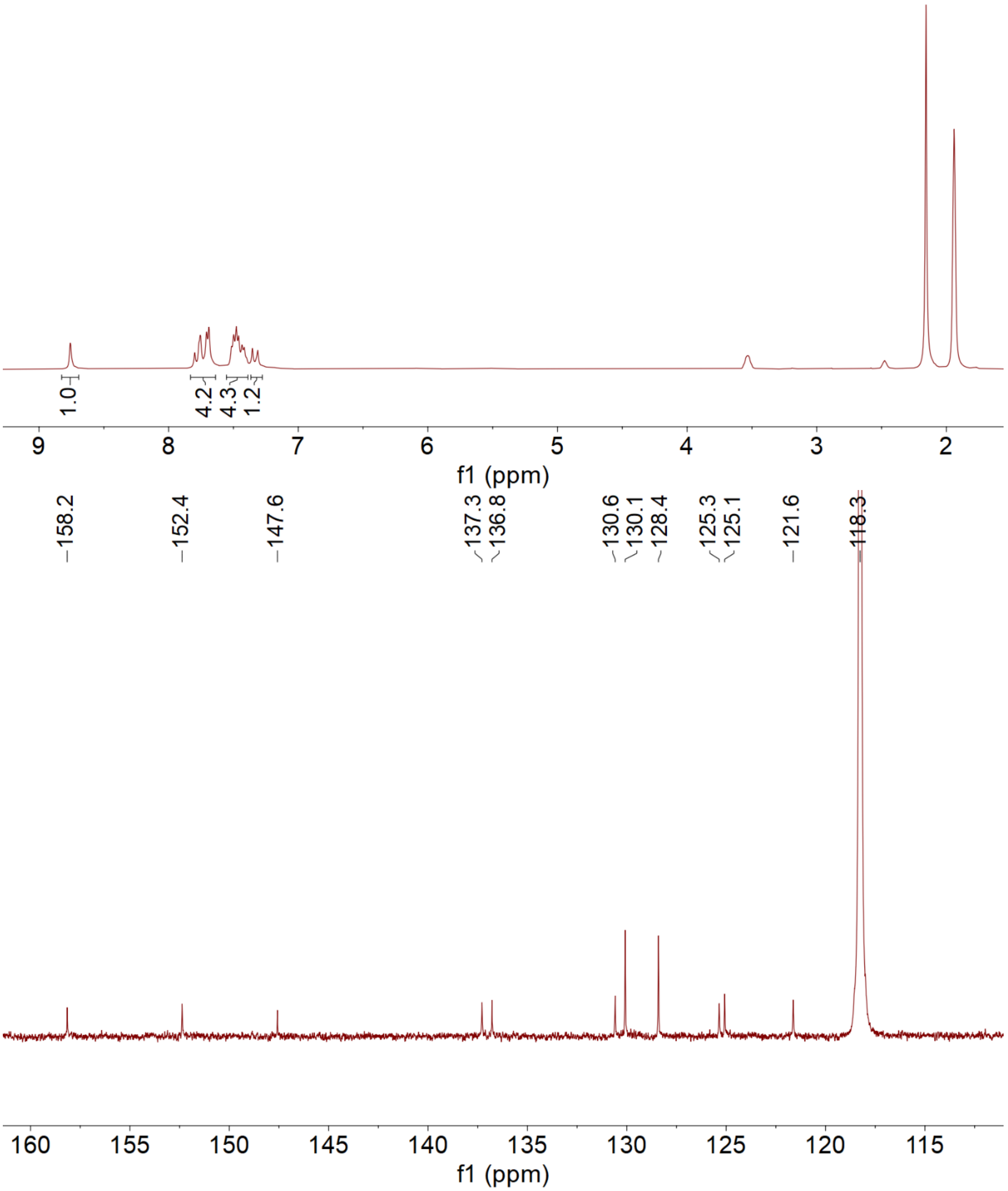

Supplementary Figure 7. ^{1}H NMR (400 MHz, CD_3CN, 298 K) (top) and $^{13}C\{^{1}H\}$ NMR (101 MHz, CD_3CN, 298 K) (bottom) spectra of **3**.

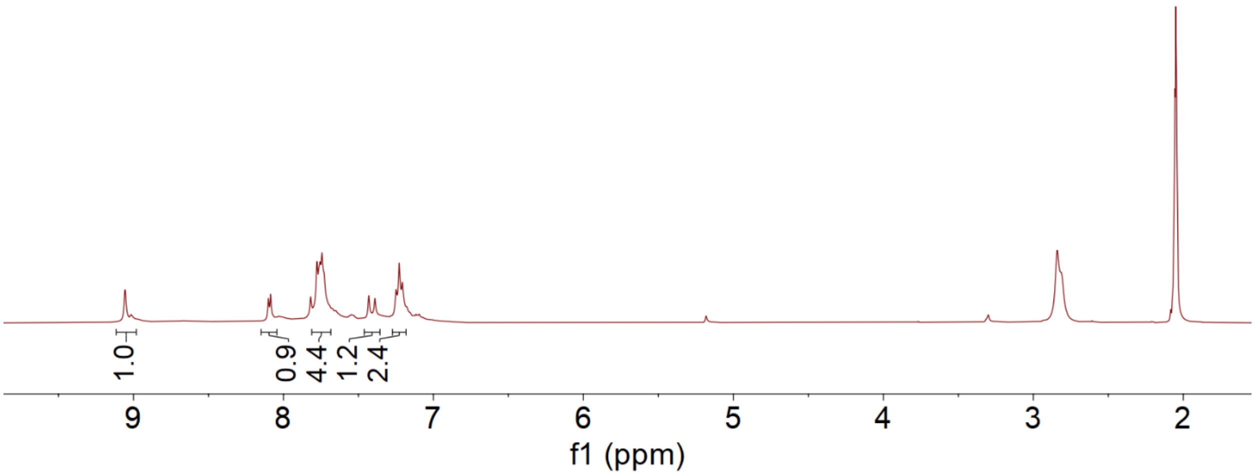
1.0
0.9
4.4
1.2
2.4
9
8
7
6
5
4
3
2
f1 (ppm)

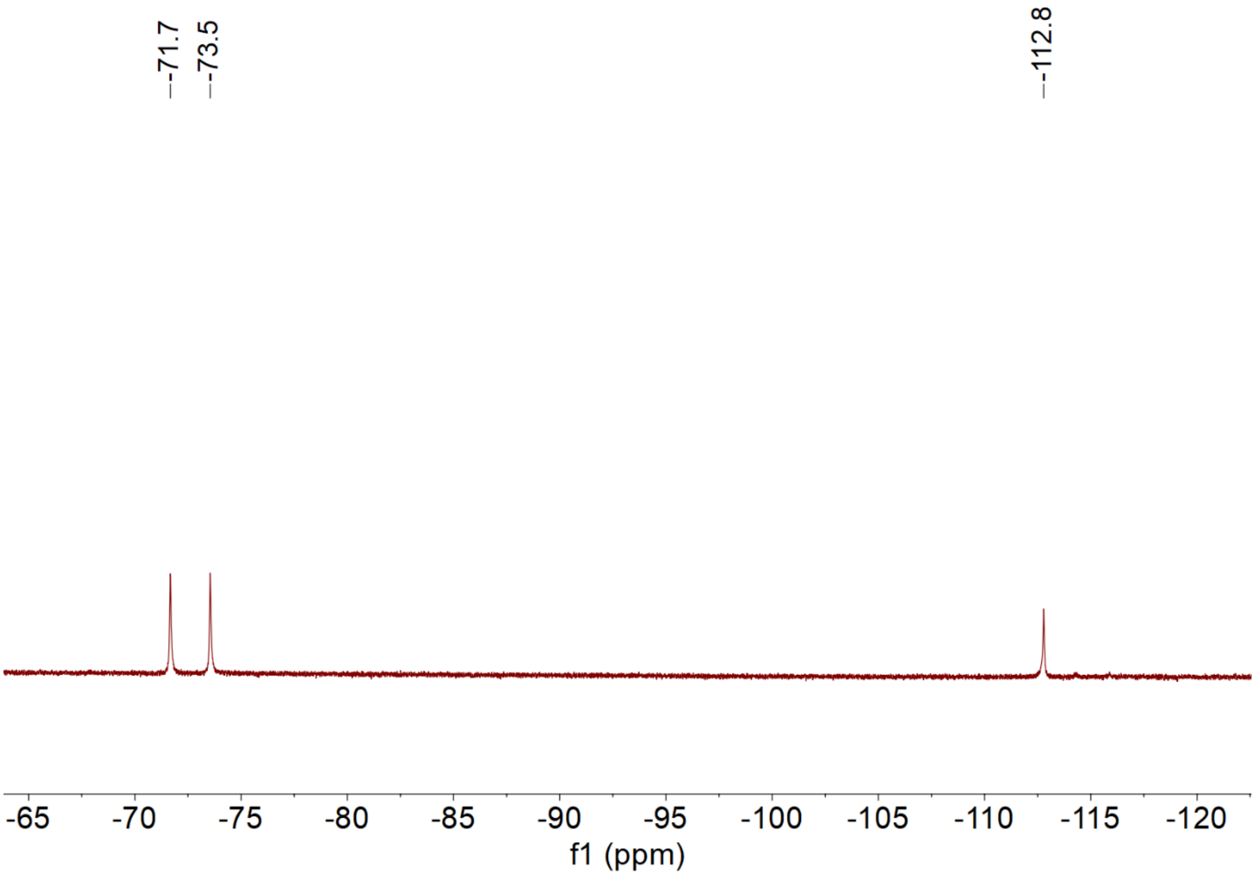
-71.7
-73.5
-112.8
-65
-70
-75
-80
-85
-90
-95
-100
-105
-110
-115
-120
f1 (ppm)

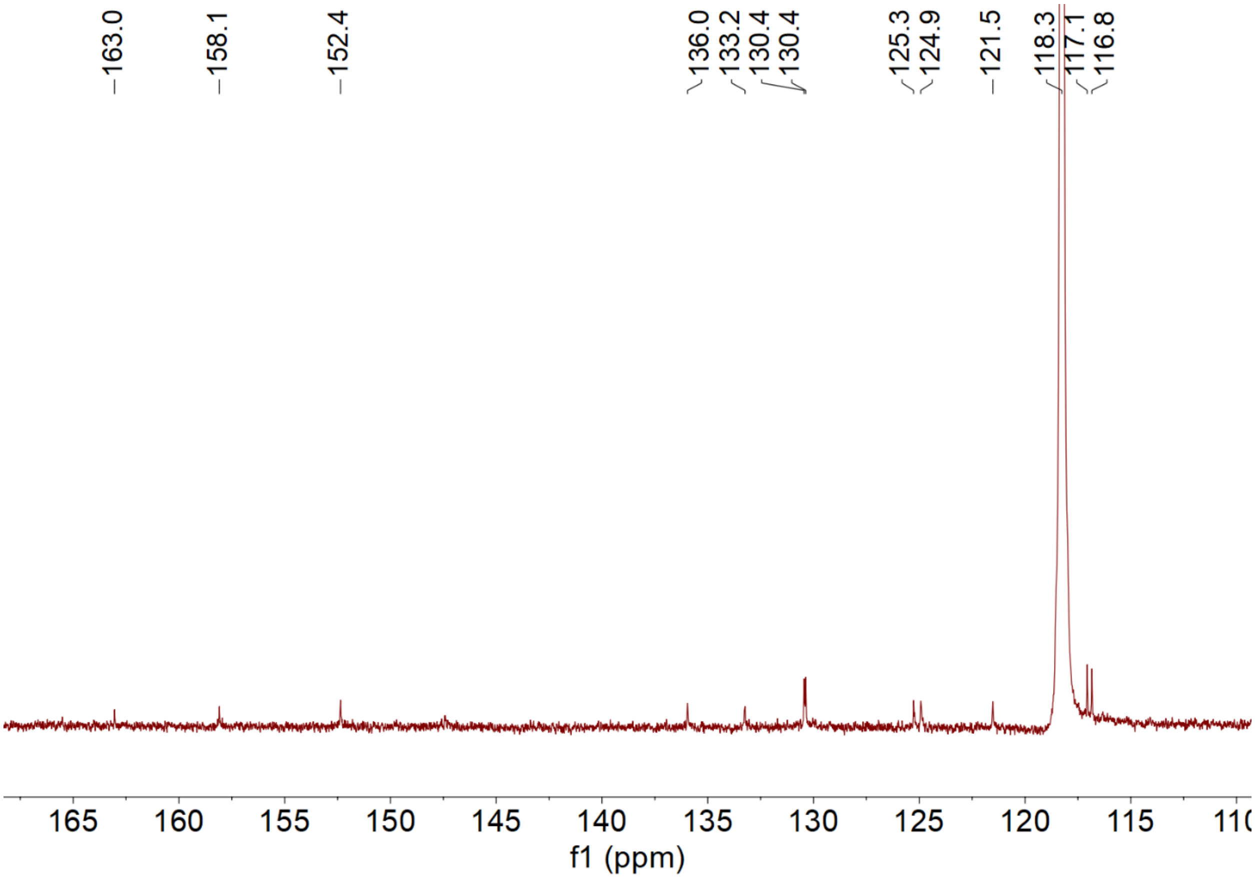

Supplementary Figure 8. ^{1}H NMR (400 MHz, CD_3COCD_3, 298 K) (top), ^{19}F NMR (376 MHz, CD_3CN, 298 K) (middle) and $^{13}C\{^{1}H\}$ NMR (101 MHz, CD_3CN, 298 K) (bottom) spectra of **4**.

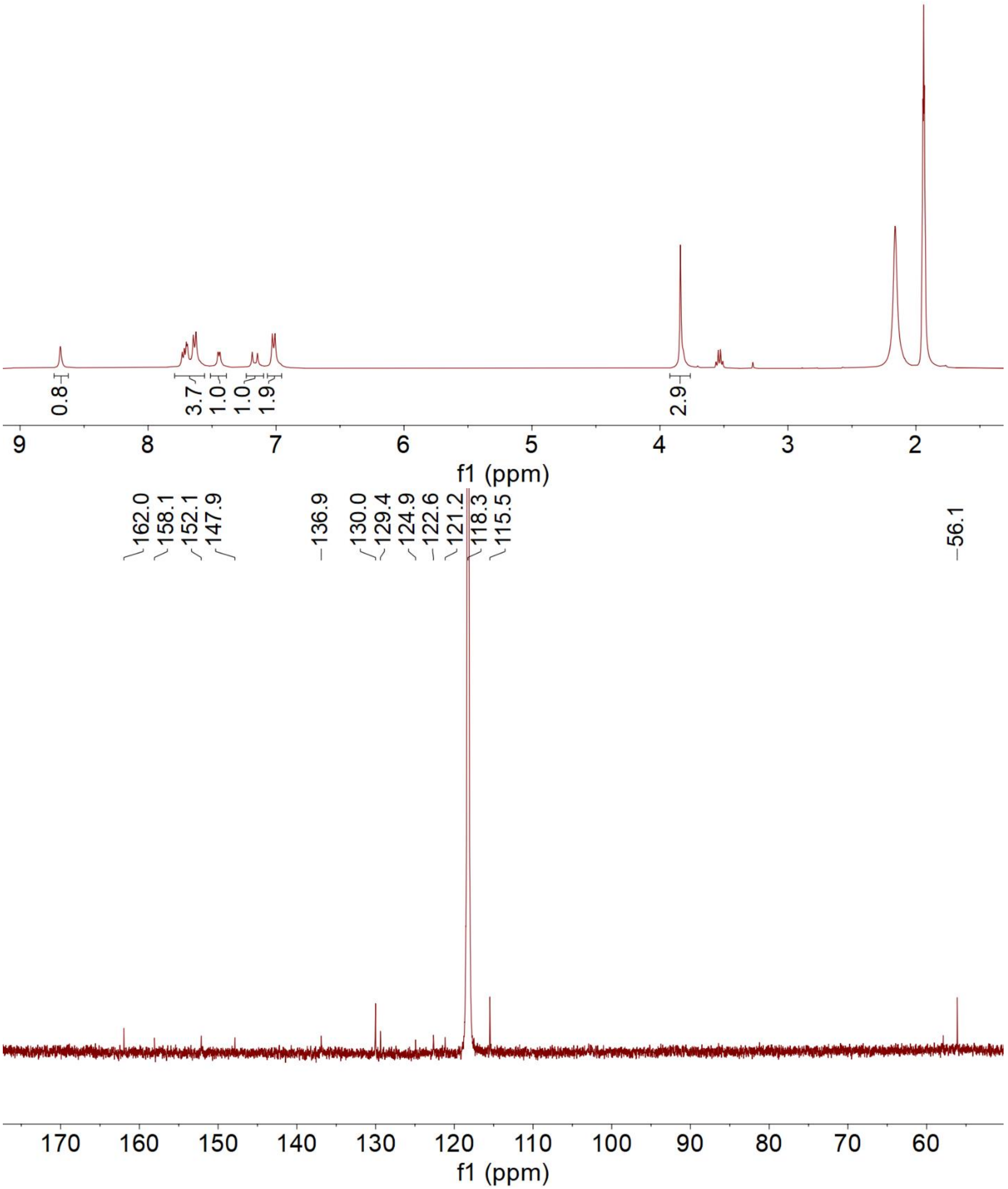

Supplementary Figure 9. ^{1}H NMR (400 MHz, CD_3CN, 298 K) (top) and $^{13}C\{^{1}H\}$ NMR (101 MHz, CD_3CN, 298 K) (bottom) spectra of **5**.

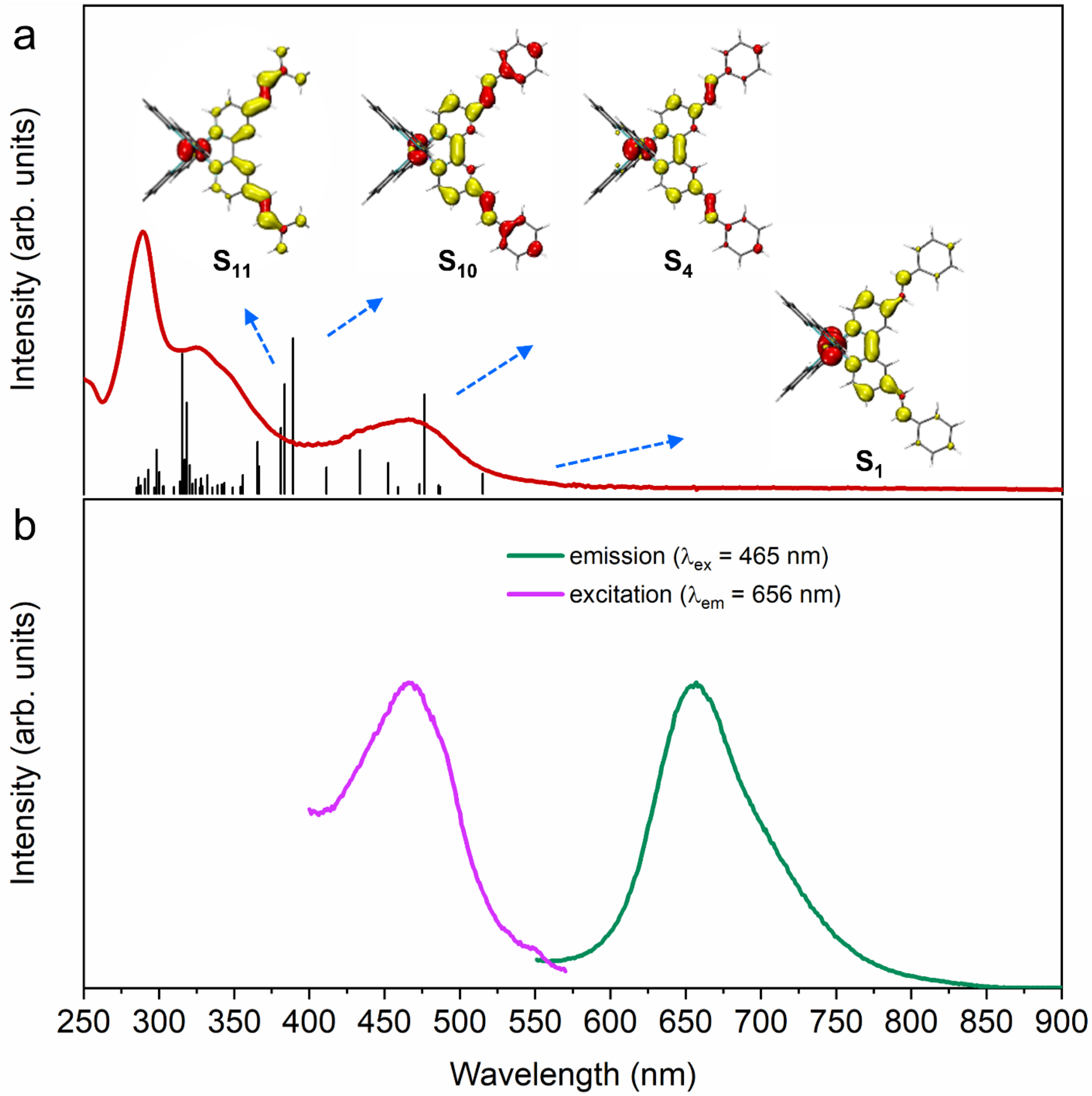

Supplementary Figure 10. (a) Absorption and (b) emission and excitation spectra of **1** in CH_3CN. *Insets* in (a) are the calculated EDDMs of selected singlet excited states of **1** (red indicates electron decrease and yellow indicates electron increase).

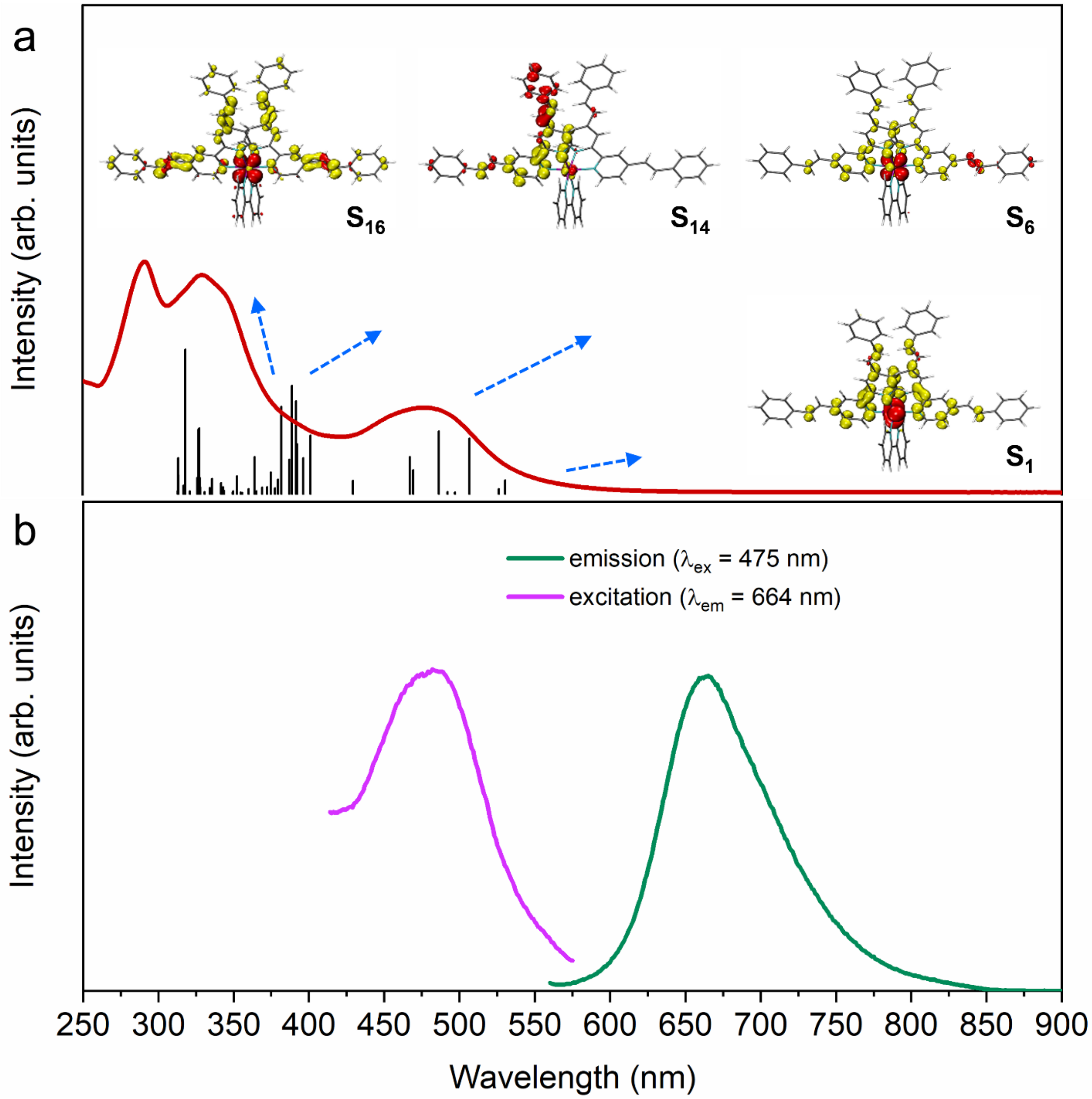

Supplementary Figure 11. (a) Absorption and (b) emission and excitation spectra of **2** in CH_3CN. *Insets* in (a) are the calculated EDDMs of selected singlet excited states of **2** (red indicates electron decrease and yellow indicates electron increase).

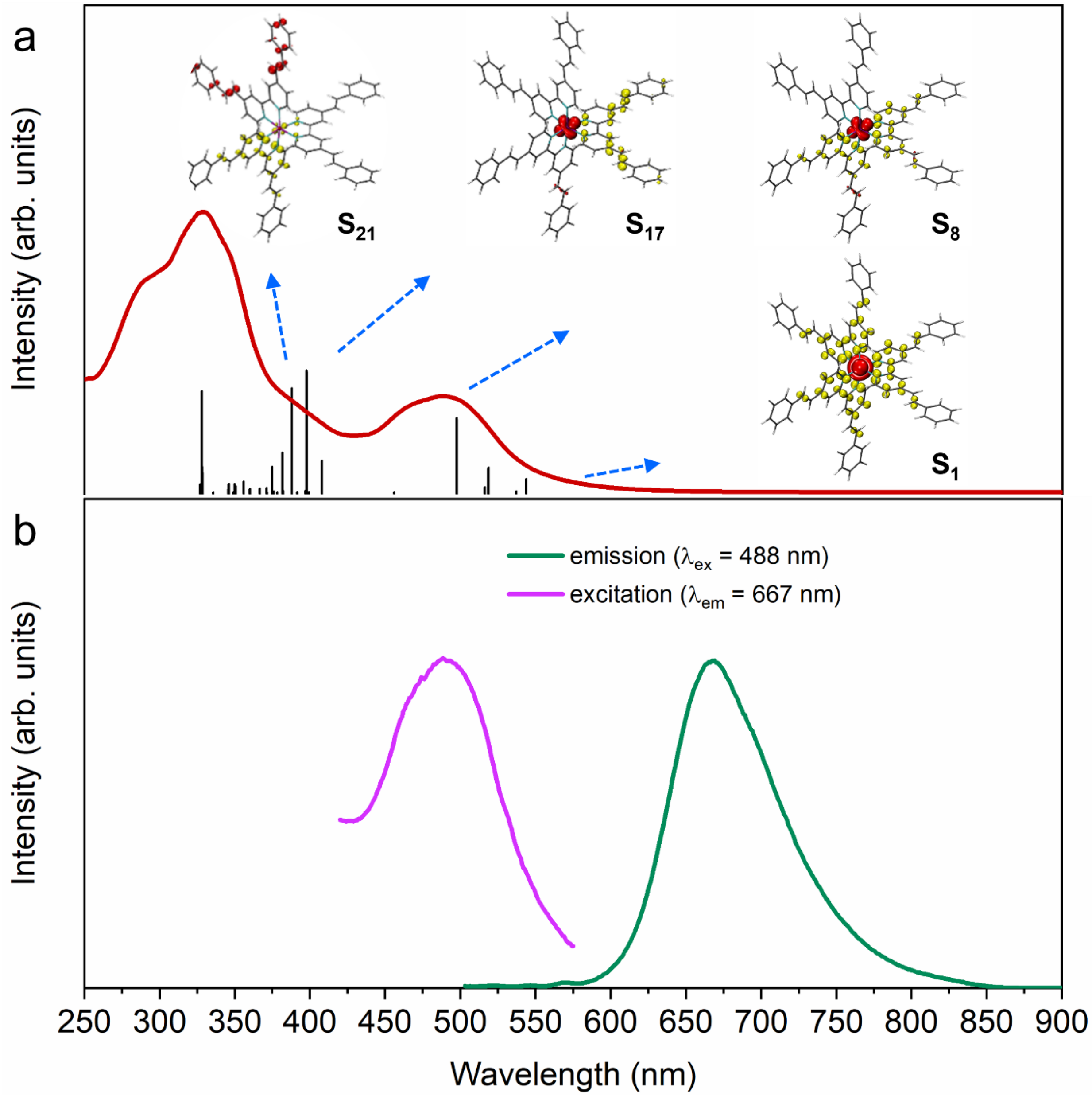

Supplementary Figure 12. (a) Absorption and (b) emission and excitation spectra of **3** in CH_3CN. *Insets* in (a) are the calculated EDDMs of selected singlet excited states of **3** (red indicates electron decrease and yellow indicates electron increase).

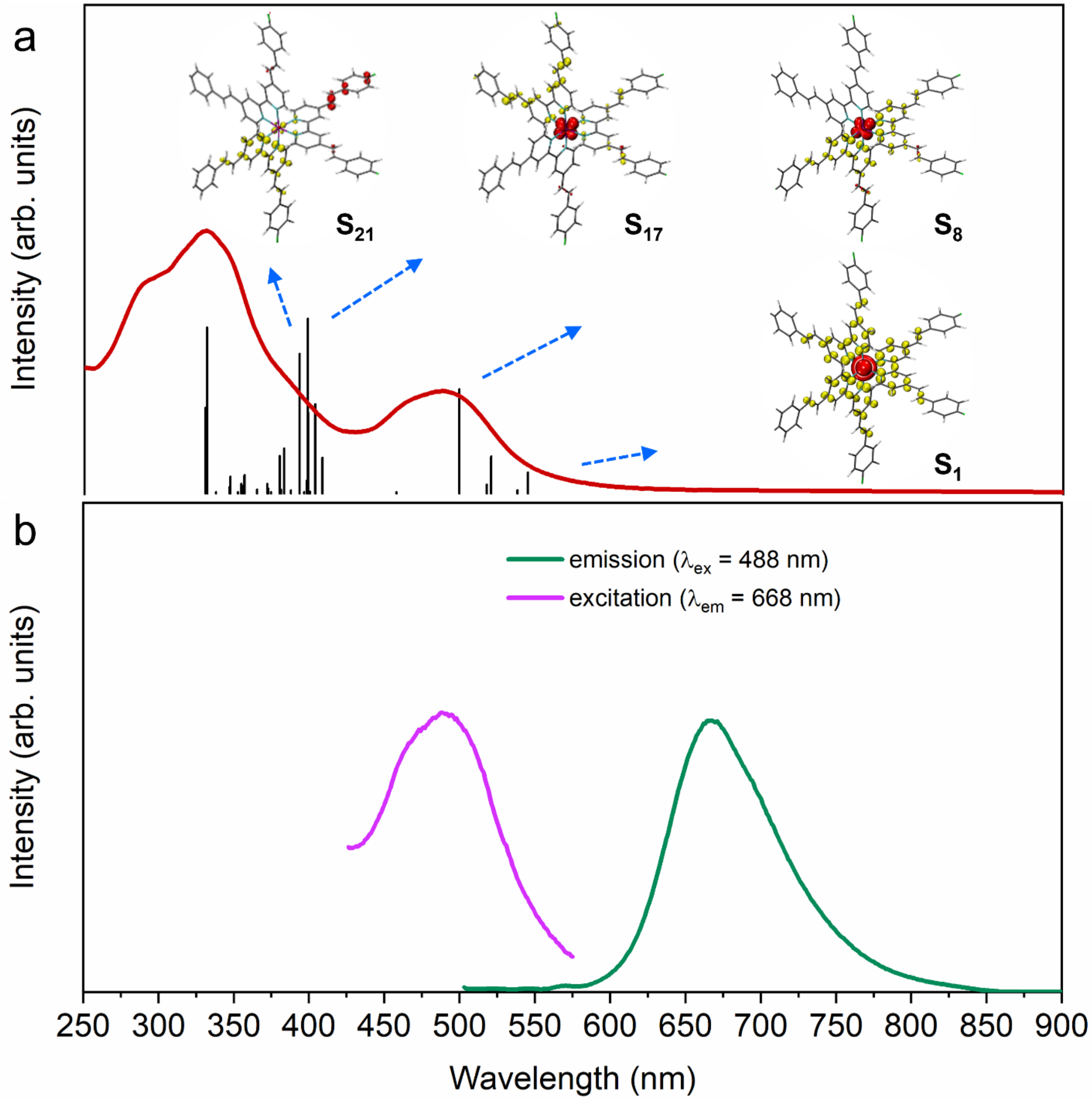

Supplementary Figure 13. (a) Absorption and (b) emission and excitation spectra of **4** in CH_3CN. *Insets* in (a) are the calculated EDDMs of selected singlet excited states of **4** (red indicates electron decrease and yellow indicates electron increase).

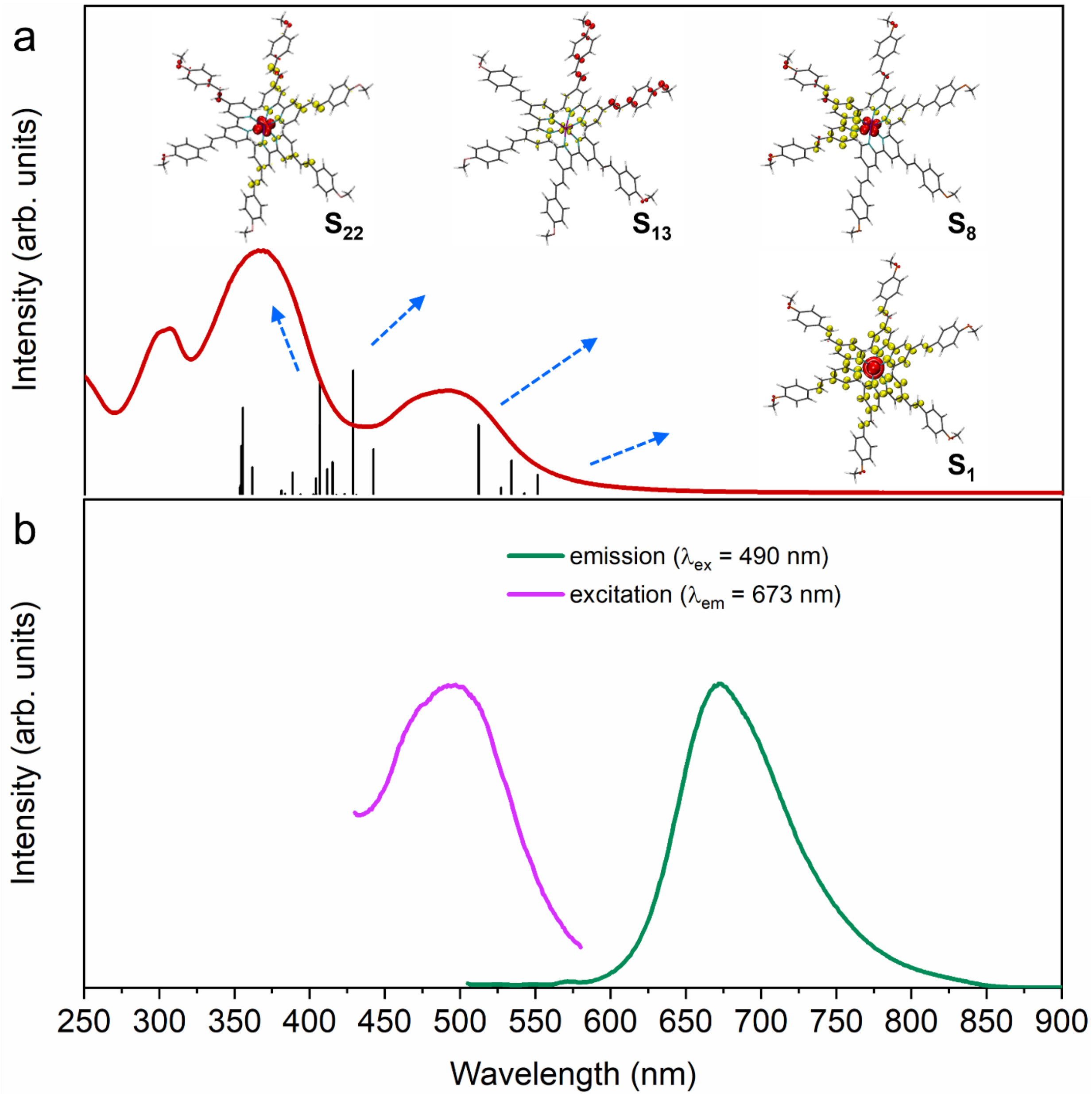

Supplementary Figure 14. (a) Absorption and (b) emission and excitation spectra of **5** in CH_3CN. *Insets* in (a) are the calculated EDDMs of selected singlet excited states of **5** (red indicates electron decrease and yellow indicates electron increase).

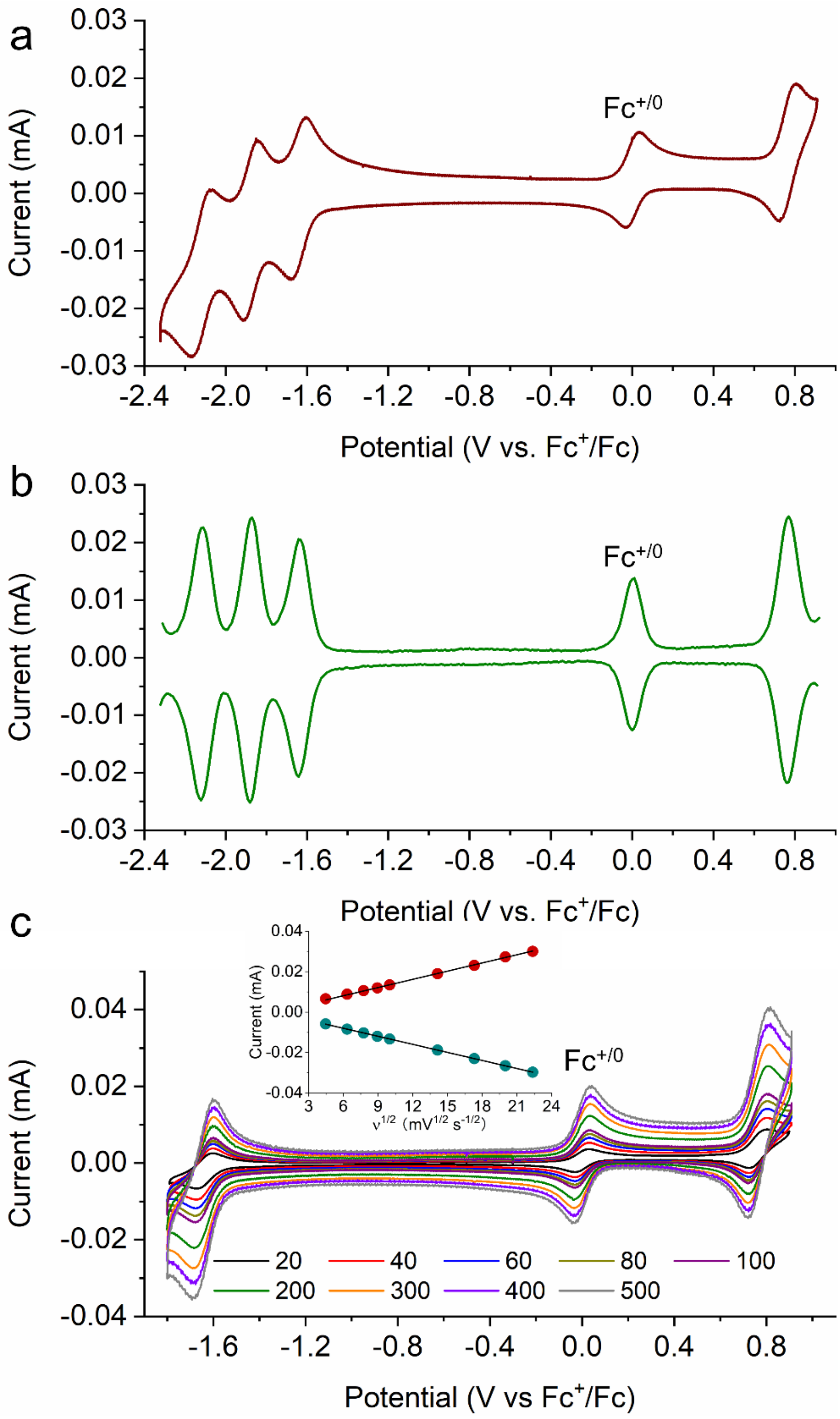

Supplementary Figure 15. Electrochemistry measurements of **1** in DMF: (a) cyclic voltammetry, (b) square wave voltammetry, and (c) cyclic voltammetry with different scan rate (v) ranging from 20 to 500 mV/s. Insert is the relationship between an oxidation peak (at 0.77 V vs $Fc^{+/0}$) or a reduction peak (at -1.64 V vs $Fc^{+/0}$) versus $v^{1/2}$. Condition: three electrode system with Pt wire as the counter electrode, glassy carbon as the working electrode and SCE as the reference electrode, 0.1 M $LiClO_4$ in deaerated DMF. Scan rate of the cyclic voltammetry is 100 mV/s.

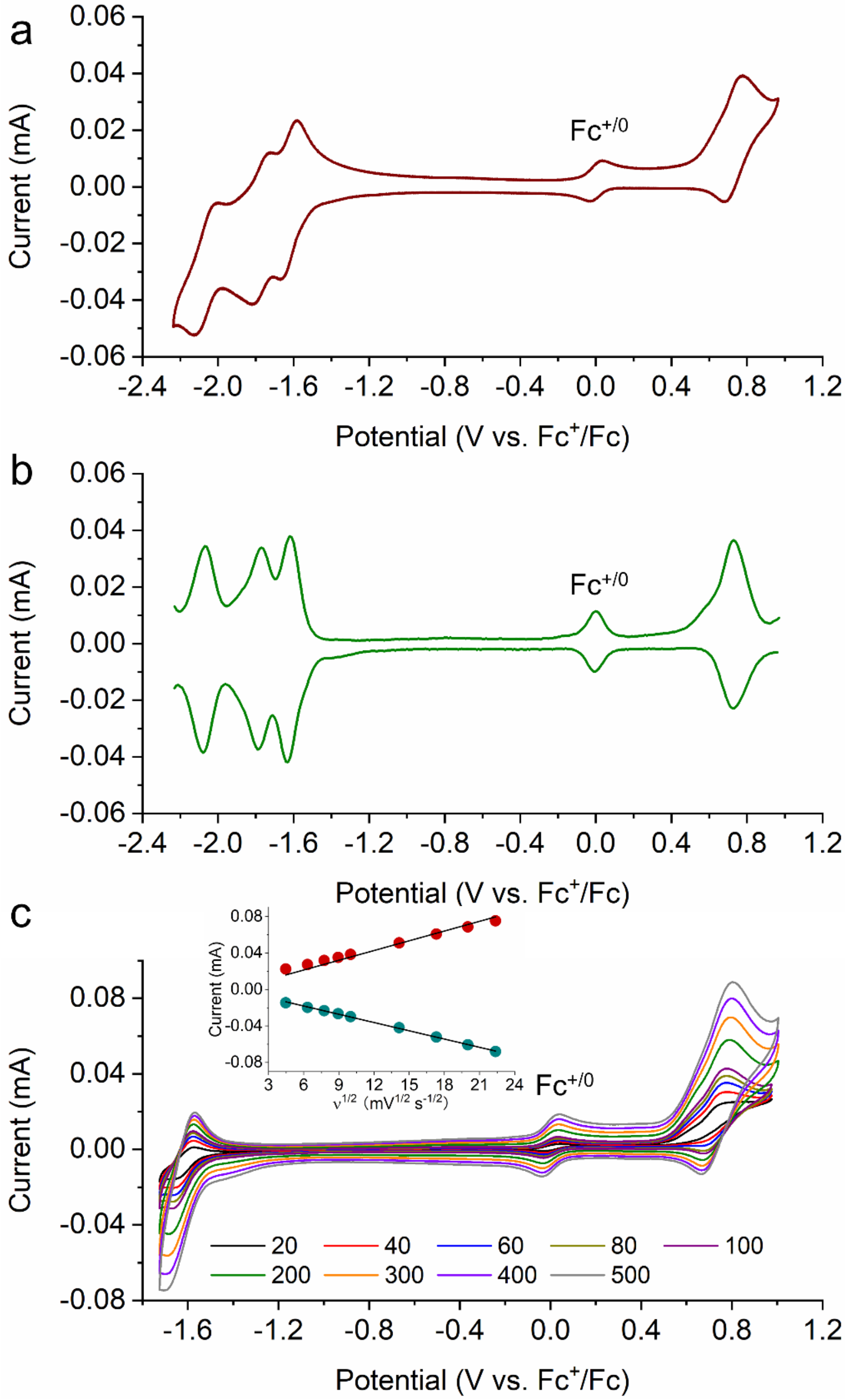

Supplementary Figure 16. Electrochemistry measurements of **2** in DMF: (a) cyclic voltammetry, (b) square wave voltammetry, and (c) cyclic voltammetry with different scan rate (v) ranging from 20 to 500 mV/s. Insert is the relationship between an oxidation peak (at 0.73 V vs $Fc^{+/0}$) or a reduction peak (at -1.63 V vs $Fc^{+/0}$) versus $v^{1/2}$. Condition: three electrode system with Pt wire as the counter electrode, glassy carbon as the working electrode and SCE as the reference electrode, 0.1 M $LiClO_4$ in deaerated DMF. Scan rate of the cyclic voltammetry is 100 mV/s.

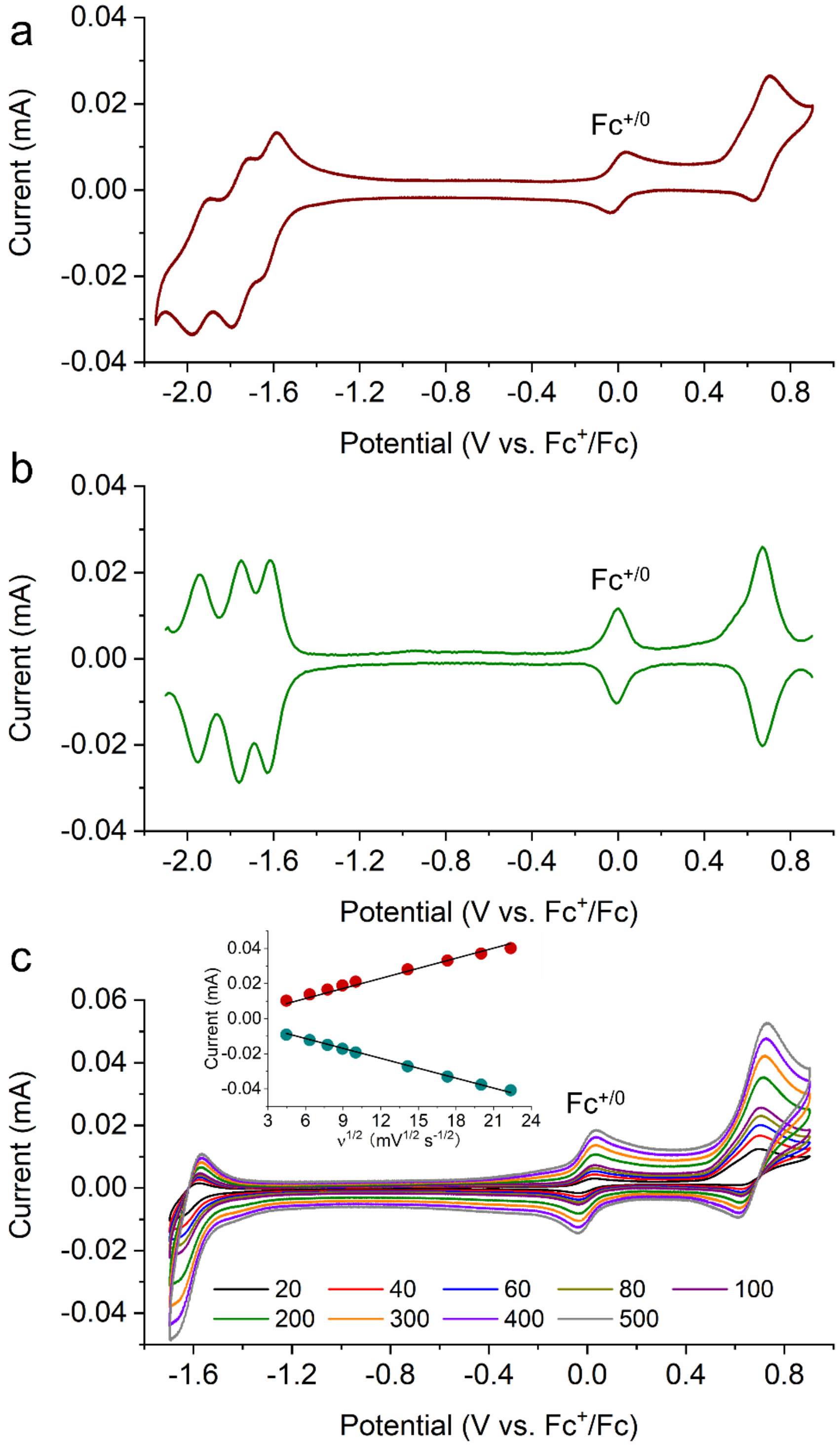

Supplementary Figure 17. Electrochemistry measurement of **3** in DMF: (a) cyclic voltammetry, (b) square wave voltammetry, and (c) cyclic voltammetry with different scan rate (v) ranging from 20 to 500 mV/s. Insert is the relationship between an oxidation peak (at 0.67 V vs $Fc^{+/0}$) or a reduction peak (at -1.62 V vs $Fc^{+/0}$) versus $v^{1/2}$. Condition: three electrode system with Pt wire as the counter electrode, glassy carbon as the working electrode and SCE as the reference electrode, 0.1 M $LiClO_4$ in deaerated DMF. Scan rate of the cyclic voltammetry is 100 mV/s.

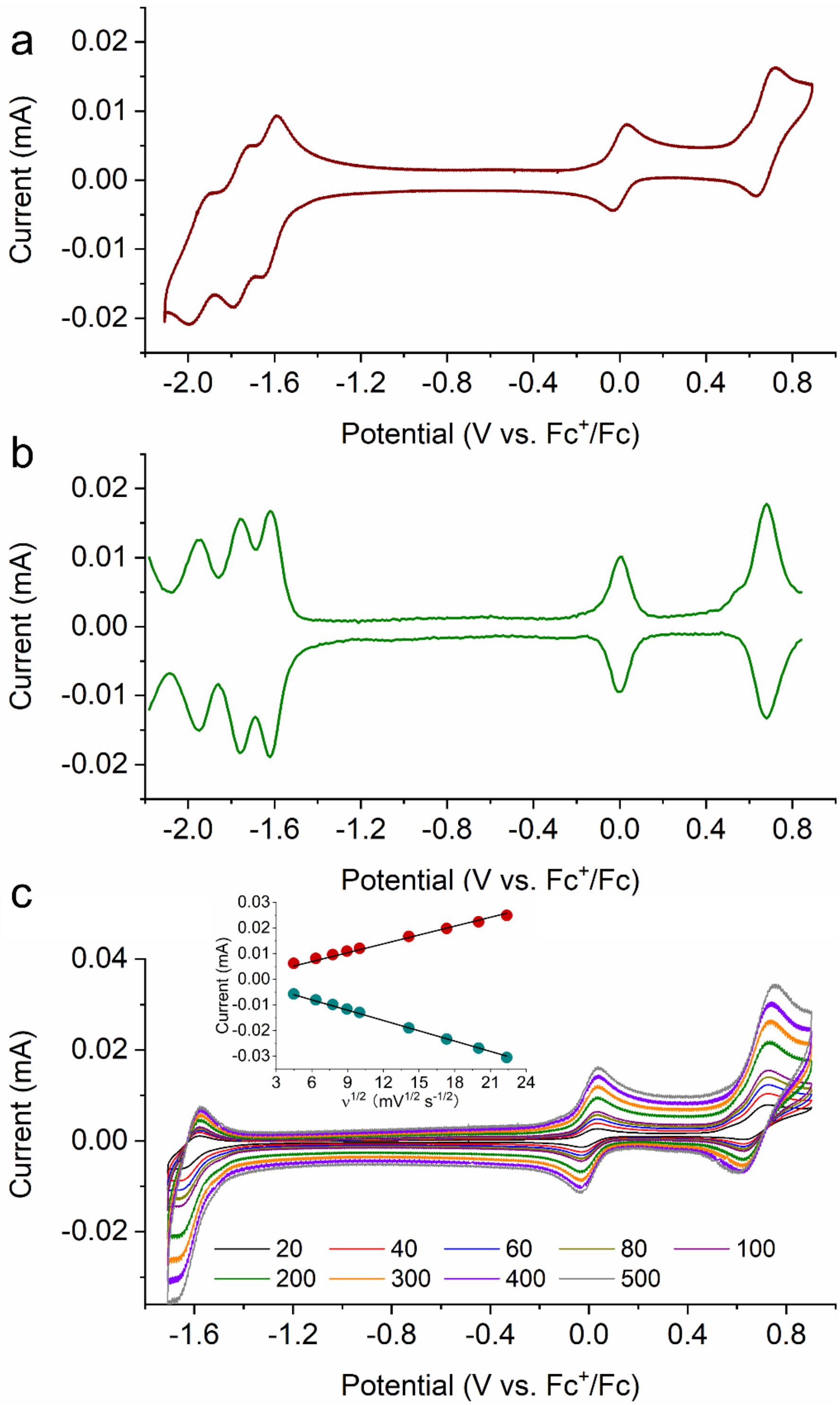

Supplementary Figure 18. Electrochemistry measurement of **4** in DMF: (a) cyclic voltammetry, (b) square wave voltammetry, and (c) cyclic voltammetry with different scan rate (v) ranging from 20 to 500 mV/s. Insert is the relationship between an oxidation peak current (at 0.68 V vs $Fc^{+/0}$) or a reduction peak (at -1.62 V vs $Fc^{+/0}$) versus $v^{1/2}$. Condition: three electrode system with Pt wire as the counter electrode, glassy carbon as the working electrode and SCE as the reference electrode, 0.1 M $LiClO_4$ in deaerated DMF. Scan rate of the cyclic voltammetry is 100 mV/s.

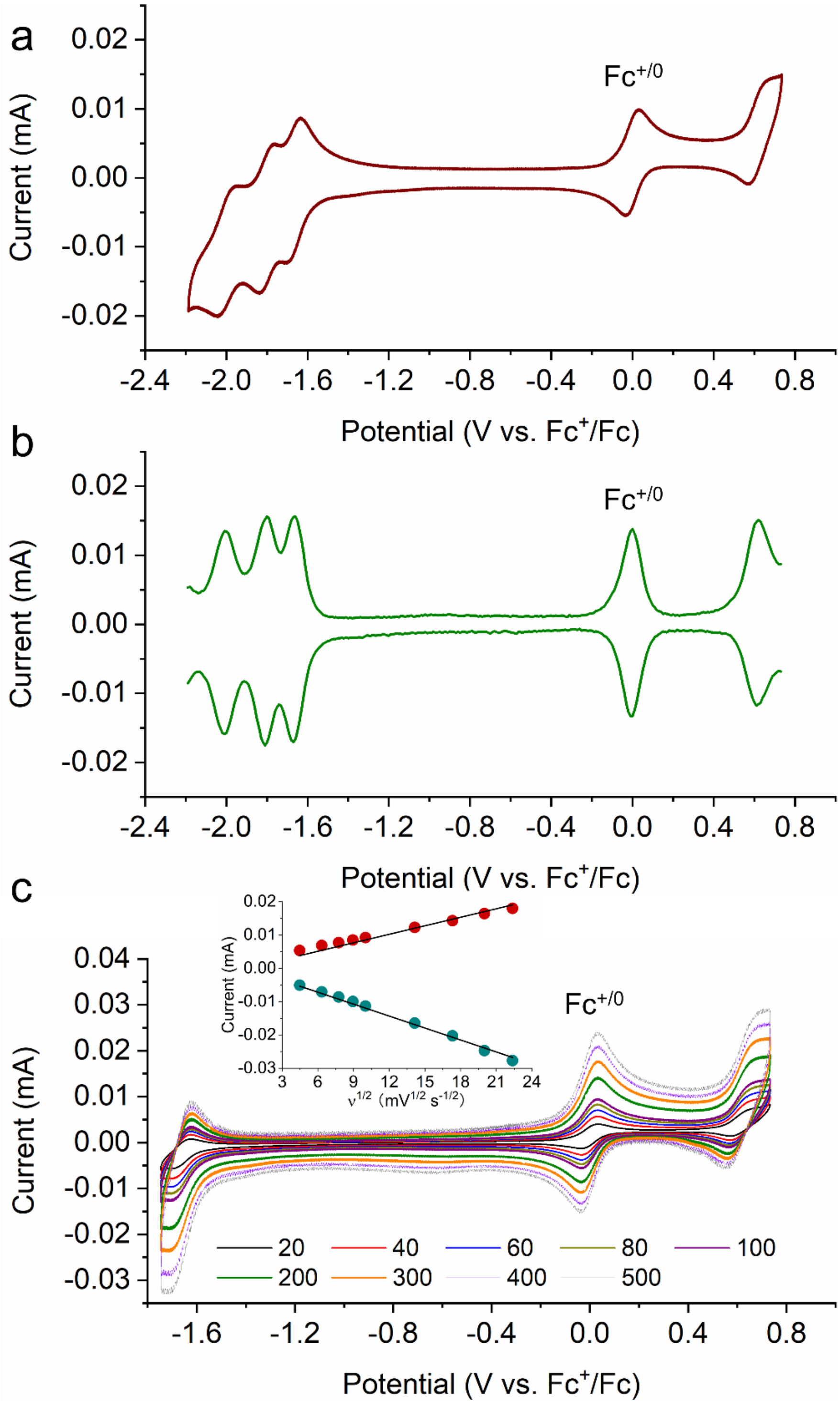

Supplementary Figure 19. Electrochemistry measurement of **5** in DMF: (a) cyclic voltammetry, (b) square wave voltammetry, and (c) cyclic voltammetry with different scan rate (v) ranging from 20 to 500 mV/s. Insert is the relationship between an oxidation peak current (at 0.62 V vs $Fc^{+/0}$) or a reduction peak (at -1.67 V vs $Fc^{+/0}$) versus $v^{1/2}$. Condition: three electrode system with Pt wire as the counter electrode, glassy carbon as the working electrode and SCE as the reference electrode, 0.1 M $LiClO_4$ in deaerated DMF. Scan rate of the cyclic voltammetry is 100 mV/s.

Supplementary Table 1. Calculated MOs of **1** (Isovalue = 0.04).

MOs	Energy (eV)	Orbitals
HOMO-5 (179)	-7.13	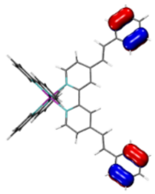
HOMO-4 (180)	-6.59	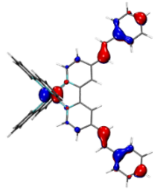
HOMO-3 (181)	-6.44	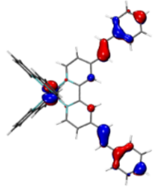
HOMO-2 (182)	-6.14	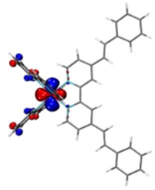
HOMO-1 (183)	-5.94	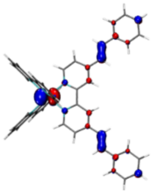
**HOMO (184)**	**-5.90**	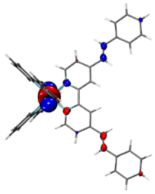
**LUMO (185)**	**-2.77**	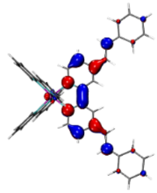

LUMO+1 (186)	-2.52	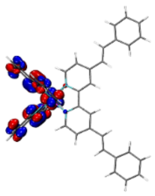
LUMO+2 (187)	-2.46	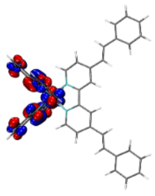
LUMO+3 (188)	-2.20	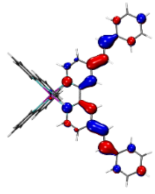
LUMO+4 (189)	-1.83	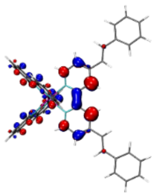
LUMO+5 (190)	-1.59	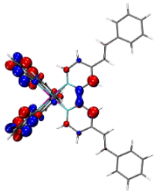

Supplementary Table 2. Calculated MOs of **2** (Isovalue = 0.04).

MOs	Energy	Orbitals
HOMO-5 (233)	-6.51	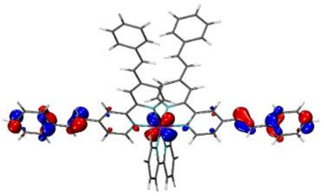
HOMO-4 (234)	6.44	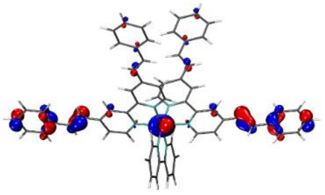
HOMO-3 (235)	-6.29	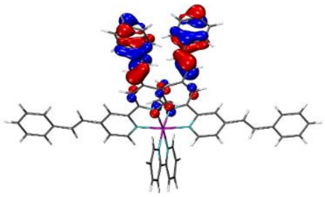
HOMO-2 (236)	-5.94	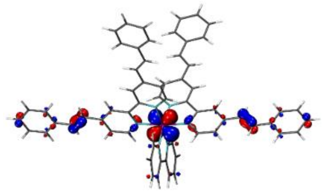
HOMO-1 (237)	-5.85	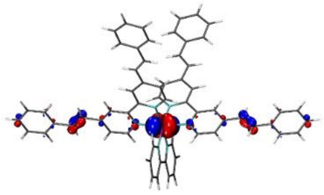
**HOMO (238)**	**-5.79**	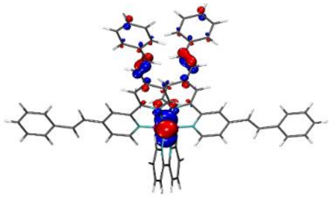
**LUMO (239)**	**-2.79**	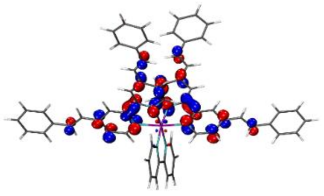

LUMO+1 (240)	-2.69	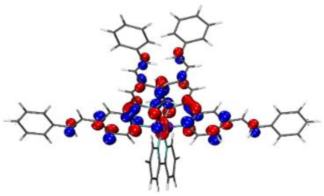
LUMO+2 (241)	-2.47	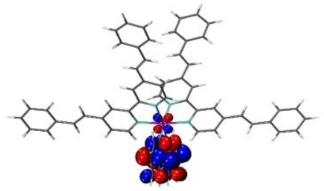
LUMO+3 (242)	-2.19	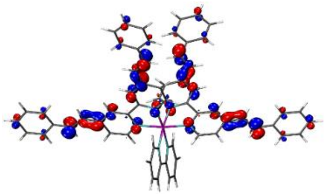
LUMO+4 (243)	-2.18	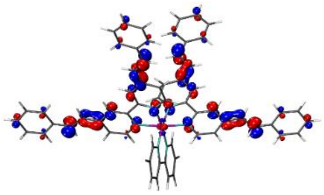
LUMO+5 (244)	-1.85	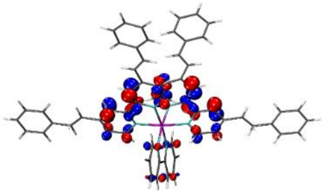

Supplementary Table 3. Calculated MOs of **3** (Isovalue = 0.04).

MOs	Energy (eV)	Orbitals
HOMO-5 (287)	-6.32	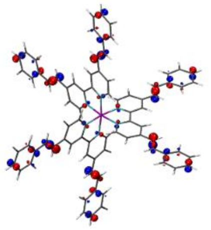
HOMO-4 (288)	-6.28	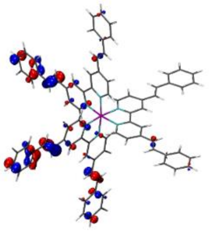
HOMO-3 (289)	-6.28	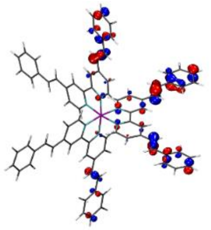
HOMO-2 (290)	-5.78	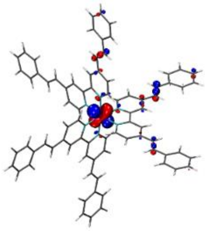
HOMO-1 (291)	-5.78	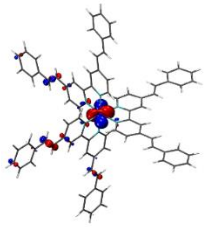
**HOMO (292)**	**-5.74**	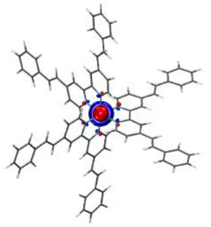

**LUMO (293)**	**-2.80**	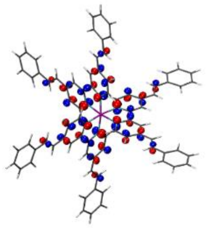
LUMO+1 (294)	-2.68	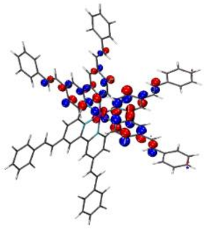
LUMO+2 (295)	-2.67	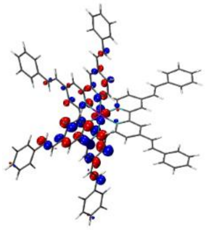
LUMO+3 (296)	-2.18	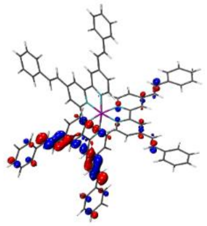
LUMO+4 (297)	-2.18	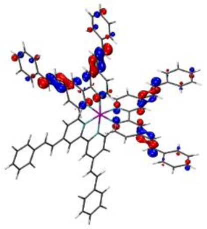
LUMO+5 (298)	-2.16	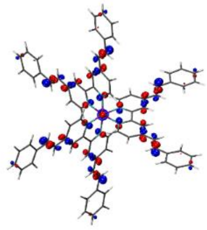

Supplementary Table 4. Calculated MOs of **4** (Isovalue = 0.04).

MOs	Energy (eV)	Orbitals
HOMO-5 (311)	-6.27	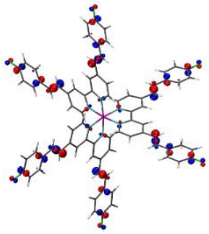
HOMO-4 (312)	-6.23	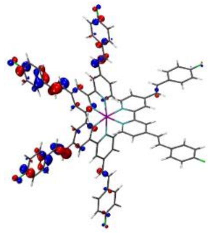
HOMO-3 (313)	-6.23	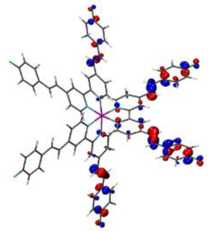
HOMO-2 (314)	-5.77	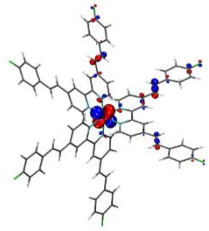
HOMO-1 (315)	-5.77	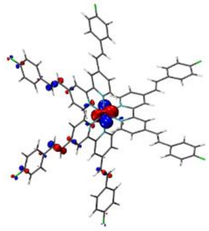
**HOMO (316)**	**-5.73**	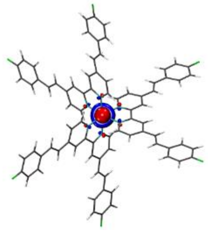

**LUMO (317)**	**-2.80**	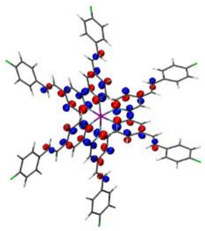
LUMO+1 (318)	-2.68	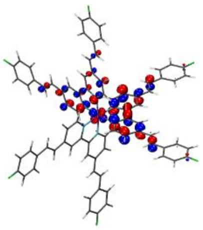
LUMO+2 (319)	-2.67	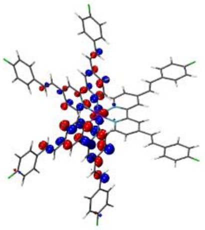
LUMO+3 (320)	-2.18	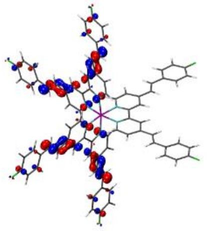
LUMO+4 (321)	-2.18	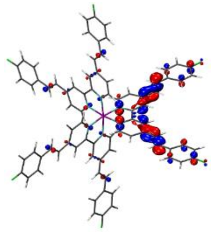
LUMO+5 (322)	-2.15	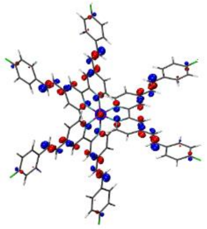

Supplementary Table 5. Calculated MOs of **5** (Isovalue = 0.04).

MOs	Energy	Orbitals
HOMO-5 (335)	-5.86	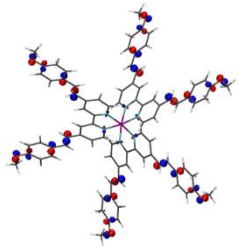
HOMO-4 (336)	-5.82	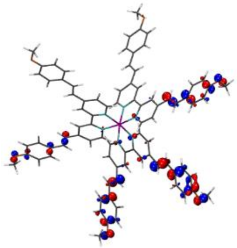
HOMO-3 (337)	-5.82	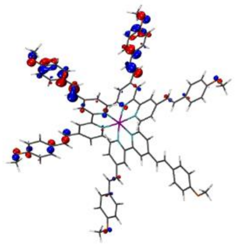
HOMO-2 (338)	-5.54	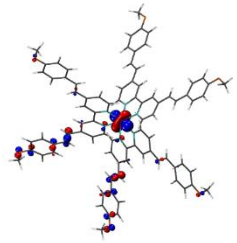
HOMO-1 (339)	-5.54	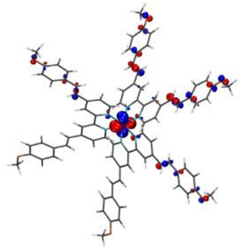
**HOMO (340)**	**-5.53**	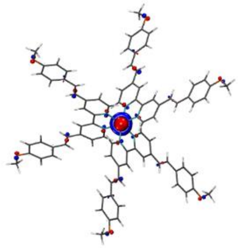

**LUMO (341)**	**-2.69**	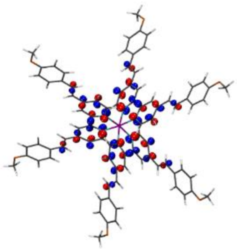
LUMO+1 (342)	-2.56	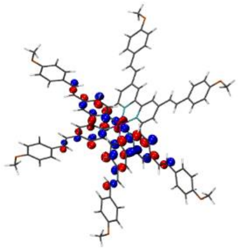
LUMO+2 (343)	-2.56	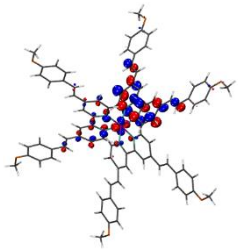
LUMO+3 (344)	-2.04	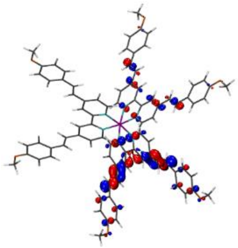
LUMO+4 (345)	-2.04	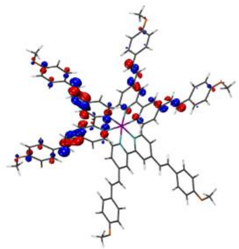
LUMO+5 (346)	-2.02	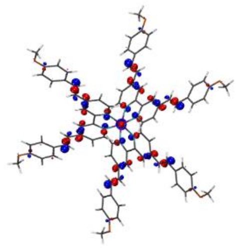

Supplementary Table 6. Calculated singlet electronic transitions (*f* > 0.01) of **1** in CH_3CN (Isovalue = 0.04).

No.	Wavelength (nm)	*f*	Major contributions	EDDM
1	515	0.050	HOMO->LUMO (89%)	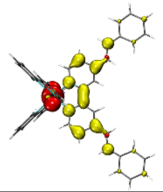
4	476	0.34	H-1->LUMO (80%), H-1->L+1 (12%)	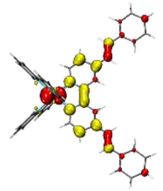
5	473	0.014	H-2->LUMO (82%)	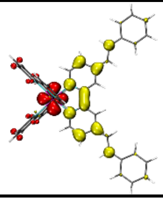
7	452	0.089	H-2->L+2 (19%), H-1->L+1 (65%)	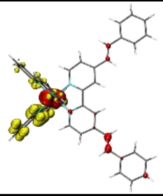
8	433	0.14	H-2->L+1 (74%), H-1->L+2 (16%)	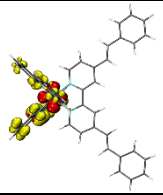
9	411	0.073	H-2->L+2 (58%), H-1->L+1 (10%), HOMO->L+3 (20%)	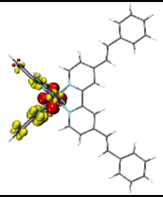
10	389	0.54	H-3->LUMO (90%)	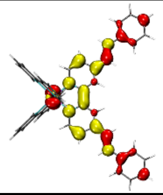
11	383	0.37	H-1->L+3 (96%)	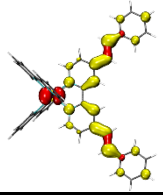

12	381	0.22	H-2->L+2 (11%), H-2->L+3 (10%), HOMO->L+3 (66%)	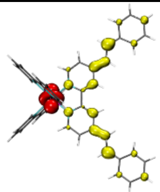
13	366	0.077	H-4->LUMO (10%), H-2->L+3 (77%)	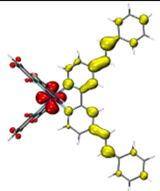
14	366	0.065	HOMO->L+4 (91%)	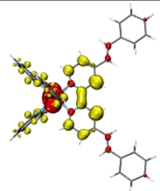
15	365	0.17	H-4->LUMO (84%)	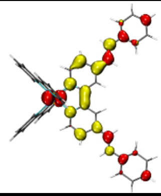
16	356	0.046	H-1->L+4 (92%)	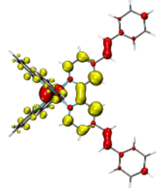
19	343	0.016	H-2->L+4 (61%), HOMO->L+5 (28%)	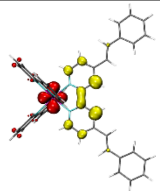
21	342	0.011	H-2->L+4 (28%), HOMO->L+5 (59%)	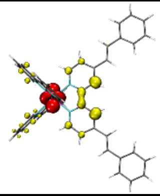
24	332	0.046	H-1->L+5 (58%), HOMO->L+7 (26%)	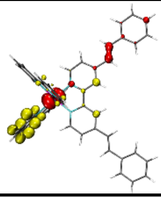

27	328	0.033	H-1->L+6 (10%), HOMO->L+8 (48%), HOMO->L+15 (10%)	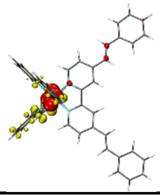
29	324	0.029	H-2->L+5 (26%), H-1->L+6 (24%), HOMO->L+8 (29%)	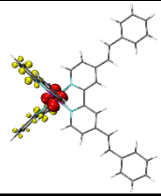
30	322	0.014	H-1->L+7 (71%)	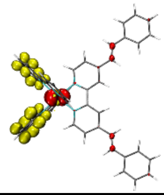
31	320	0.081	H-2->L+5 (55%), H-1->L+6 (17%)	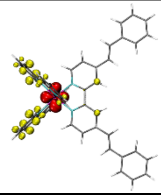
32	318	0.31	H-3->L+3 (74%), H-1->L+8 (13%)	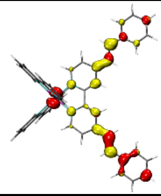
33	317	0.028	H-2->L+6 (69%), H-2->L+7 (12%)	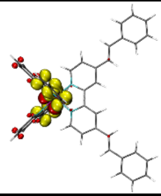
34	317	0.10	H-2->L+7 (24%), H-1->L+8 (49%)	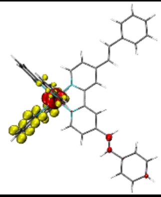
35	317	0.051	H-2->L+15 (36%), H-1->L+16 (21%)	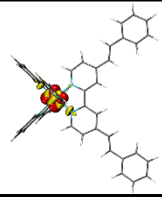

36	315	0.49	H-7->LUMO (19%), H-4->L+3 (64%)	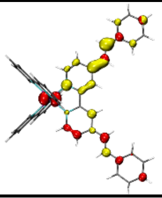
37	314	0.022	H-5->LUMO (92%)	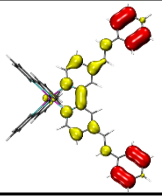
42	300	0.057	H-9->LUMO (31%), H-3->L+4 (53%)	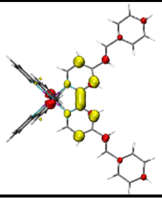
43	298	0.14	H-9->LUMO (59%), H-3->L+4 (17%)	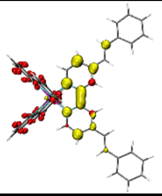
45	293	0.065	H-4->L+4 (84%)	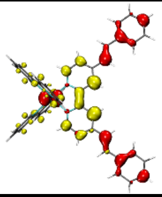
46	290	0.033	H-7->L+1 (92%)	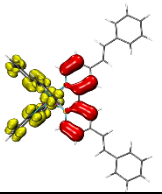
48	286	0.036	H-9->L+2 (25%), H-8->L+1 (39%), H-7->L+2 (31%)	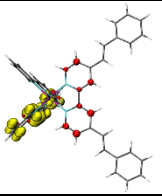

Supplementary Table 7. Calculated singlet electronic transitions (*f* > 0.01) of **2** in CH_3CN (Isovalue = 0.04).

No.	Wavelength (nm)	*f*	Major contributions	EDDM
1	530	0.063	H-1->LUMO (19%), HOMO->LUMO (74%)	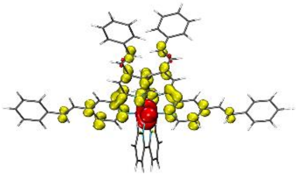
2	526	0.019	H-1->L+1 (33%), HOMO->L+1 (60%)	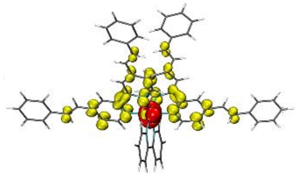
3	507	0.28	H-1->LUMO (70%), HOMO->LUMO (19%)	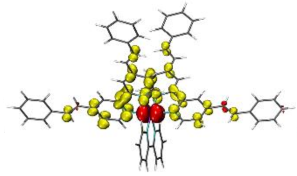
6	486	0.31	H-2->L+1 (71%), H-1->L+2 (10%)	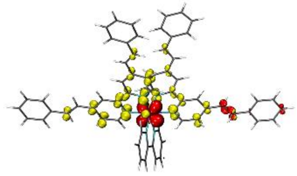
7	469	0.12	H-2->L+1 (15%), H-1->L+2 (63%), HOMO->L+2 (15%)	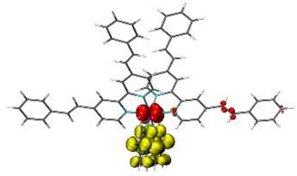
8	467	0.18	H-2->LUMO (20%), H-2->L+2 (15%), H-1->L+1 (35%), HOMO->L+1 (20%)	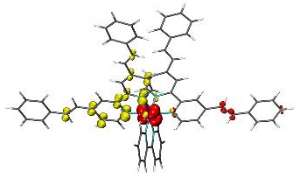
9	429	0.061	H-2->L+2 (68%), HOMO->L+4 (13%)	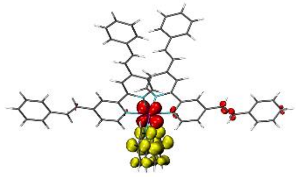
10	401	0.29	HOMO->L+3 (87%)	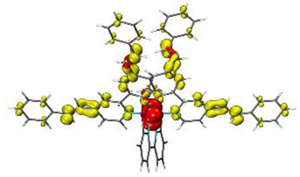

11	396	0.18	H-3->LUMO (85%)	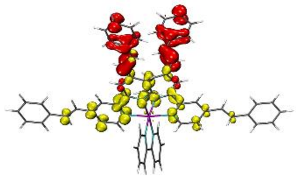
12	392	0.25	H-1->L+4 (70%), HOMO->L+4 (25%)	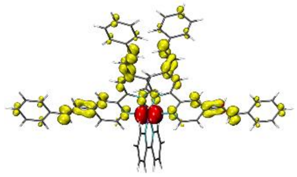
13	392	0.47	H-1->L+3 (87%), HOMO->L+3 (10%)	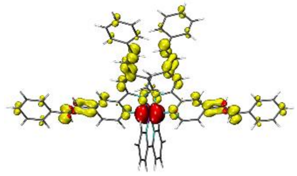
14	389	0.55	H-4->LUMO (39%), H-3->L+1 (53%)	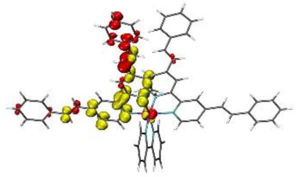
15	387	0.17	H-2->L+3 (35%), H-1->L+4 (18%), HOMO->L+4 (28%)	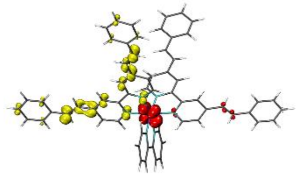
16	382	0.44	H-2->L+4 (90%)	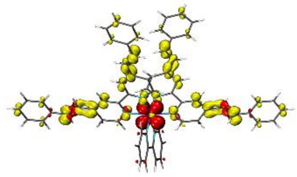
17	380	0.067	H-4->LUMO (49%), H-3->L+1 (40%)	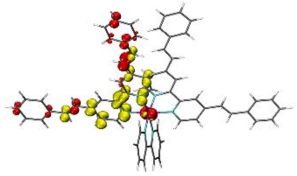
18	378	0.022	H-5->LUMO (42%), H-4->L+1 (29%), H-2->L+3 (15%)	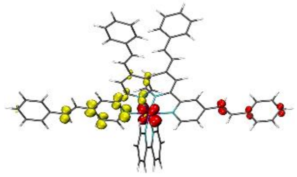

19	375	0.10	H-1->L+5 (12%), HOMO->L+5 (80%)	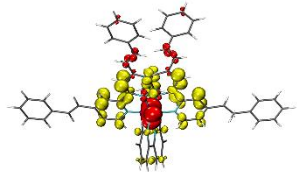
20	372	0.027	H-4->L+1 (17%), H-2->L+3 (38%), HOMO->L+4 (15%)	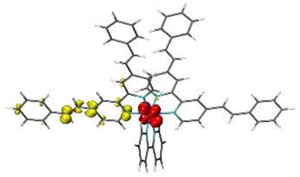
21	369	0.026	H-5->LUMO (48%), H-4->L+1 (39%)	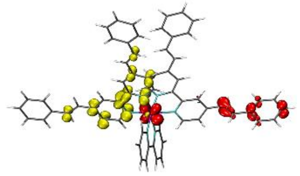
23	364	0.18	H-5->L+1 (71%), H-1->L+5 (15%)	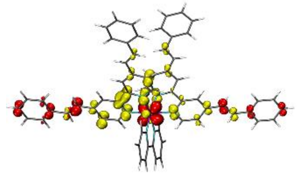
24	360	0.019	H-6->LUMO (90%)	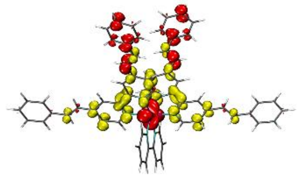
27	352	0.085	H-6->L+1 (25%), HOMO->L+6 (55%)	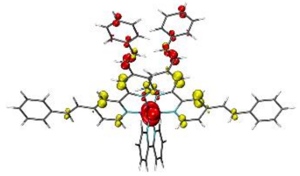
31	343	0.028	HOMO->L+7 (72%)	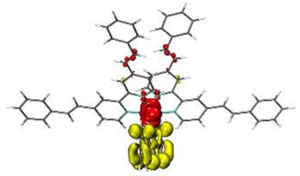
32	342	0.050	H-1->L+6 (52%), HOMO->L+6 (17%)	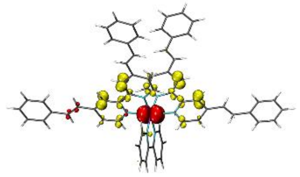

33	336	0.070	H-2->L+6 (64%), H-1->L+7 (17%)	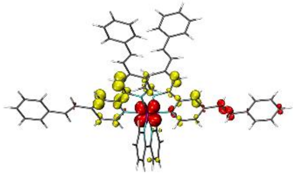
35	334	0.025	HOMO->L+8 (81%)	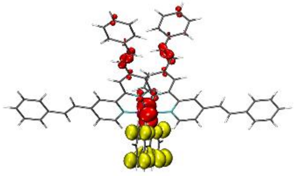
37	328	0.026	HOMO->L+19 (56%)	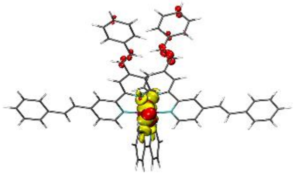
38	328	0.075	H-3->L+3 (18%), H-1->L+20 (30%)	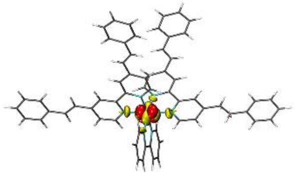
39	327	0.33	H-3->L+4 (91%)	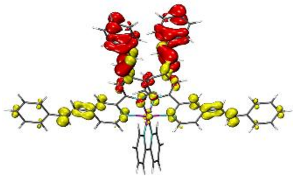
40	327	0.32	H-3->L+3 (58%), H-1->L+8 (23%)	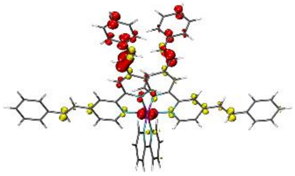
41	326	0.076	H-3->L+3 (12%), H-2->L+7 (30%), H-1->L+8 (27%), H-1->L+20 (10%)	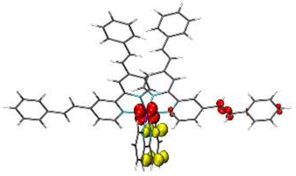
43	318	0.73	H-5->L+4 (21%), H-4->L+3 (62%)	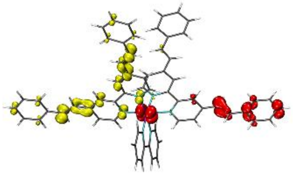

44	317	0.035	H-2->L+20 (25%), H-1->L+19 (22%)	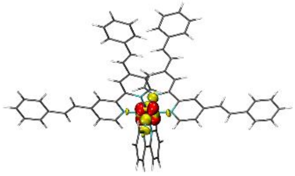
46	313	0.088	H-5->L+3 (18%), H-4->L+4 (43%)	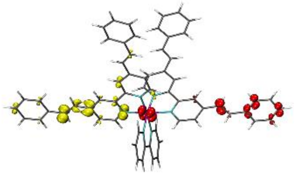
47	313	0.18	H-5->L+4 (43%), H-4->L+3 (23%)	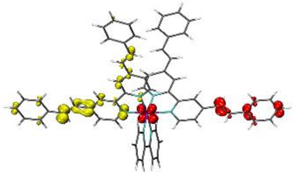
48	313	0.030	H-8->LUMO (16%), H-8->L+1 (18%), H-7->LUMO (48%)	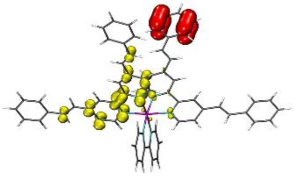
49	313	0.048	H-8->LUMO (47%), H-7->LUMO (18%), H-7->L+1 (18%)	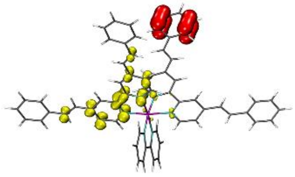

Supplementary Table 8. Calculated singlet electronic transitions (*f* > 0.01) of **3** in CH_3CN (Isovalue = 0.04).

No.	Wavelength (nm)	*f*	Major contributions	EDDM
1	544	0.085	HOMO->LUMO (96%)	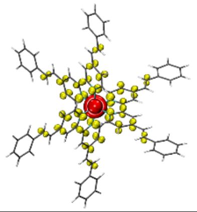
4	519	0.15	H-1->LUMO (94%)	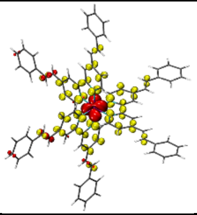
5	518	0.15	H-2->LUMO (94%)	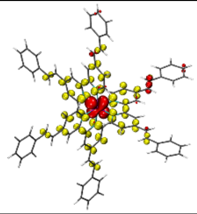
6	516	0.033	H-2->L+2 (45%), H-1->L+1 (48%)	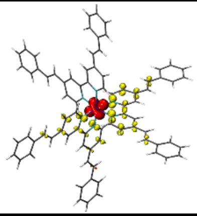
7	498	0.45	H-2->L+1 (25%), H-2->L+2 (22%), H-1->L+1 (21%), H-1->L+2 (24%)	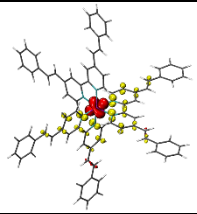
8	497	0.46	H-2->L+1 (22%), H-2->L+2 (25%), H-1->L+1 (24%), H-1->L+2 (22%)	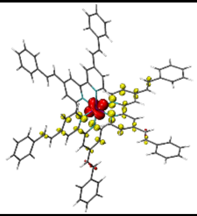
10	408	0.20	HOMO->L+3 (95%)	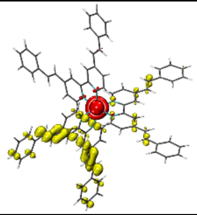

11	408	0.19	HOMO->L+4 (95%)	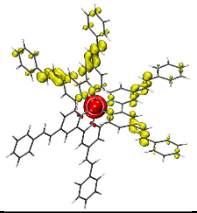
14	398	0.034	H-3->LUMO (53%), H-2->L+5 (17%), H-1->L+4 (10%)	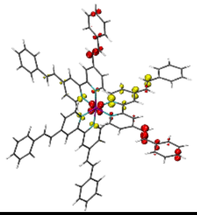
15	398	0.74	H-4->LUMO (42%), H-1->L+5 (46%)	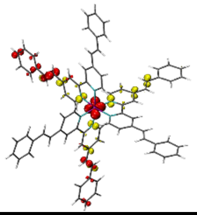
16	398	0.71	H-3->LUMO (30%), H-2->L+5 (51%)	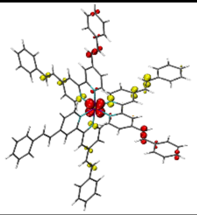
17	398	0.75	H-2->L+4 (38%), H-1->L+3 (38%)	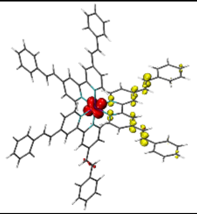
19	397	0.013	H-2->L+3 (28%), H-2->L+4 (11%), H-2->L+5 (24%), H-1->L+4 (23%)	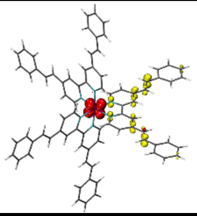
21	388	0.64	H-6->LUMO (20%), H-4->L+2 (34%), H-3->L+1 (37%)	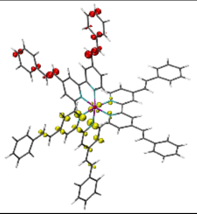
24	382	0.25	HOMO->L+6 (91%)	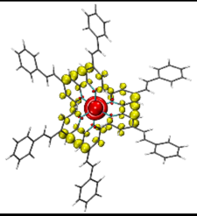

27	375	0.16	H-5->L+1 (81%)	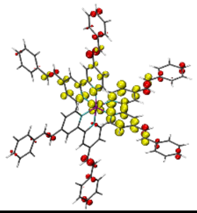
28	375	0.16	H-5->L+2 (81%)	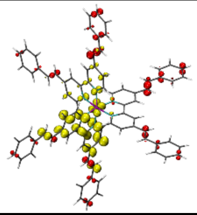
30	371	0.029	H-1->L+6 (93%)	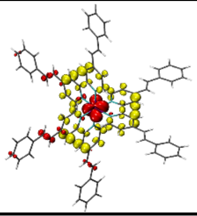
31	371	0.029	H-2->L+6 (93%)	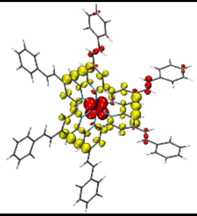
32	367	0.024	H-8->LUMO (13%), H-6->L+1 (73%)	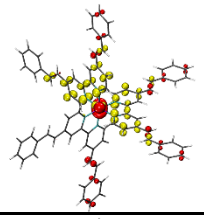
33	367	0.024	H-7->LUMO (13%), H-6->L+2 (73%)	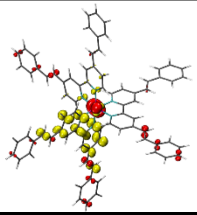
34	360	0.021	H-7->LUMO (77%), H-6->L+2 (14%)	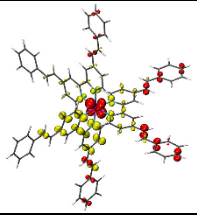
35	360	0.021	H-8->LUMO (77%), H-6->L+1 (13%)	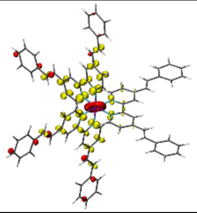

36	356	0.069	HOMO->L+7 (92%)	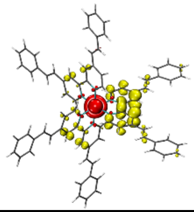
37	356	0.067	HOMO->L+8 (91%)	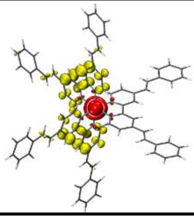
38	350	0.037	H-8->L+1 (43%), H-7->L+2 (41%)	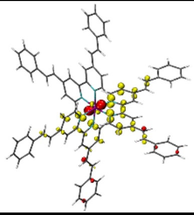
39	350	0.054	H-8->L+2 (33%), H-7->L+1 (43%)	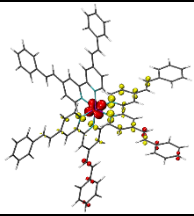
40	350	0.053	H-8->L+1 (38%), H-7->L+2 (38%)	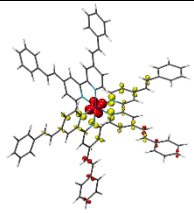
42	346	0.054	H-2->L+7 (22%), H-1->L+7 (26%), H-1->L+8 (29%)	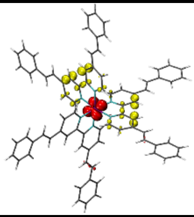
43	346	0.053	H-2->L+7 (21%), H-2->L+8 (14%), H-1->L+7 (38%), H-1->L+8 (13%)	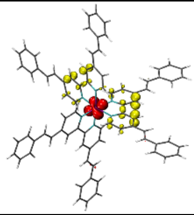
44	346	0.021	H-2->L+8 (62%), H-1->L+7 (20%)	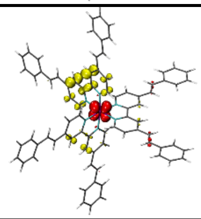

46	328	0.12	H-2->L+23 (10%), H-1->L+22 (10%), HOMO->L+22 (32%), HOMO->L+23 (11%)	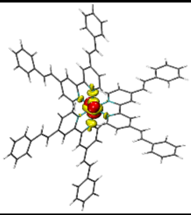
47	328	0.16	H-2->L+22 (11%), H-1->L+23 (11%), HOMO->L+23 (29%)	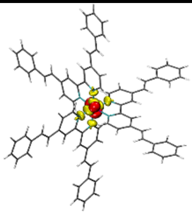
48	328	0.62	H-5->L+4 (15%), H-4->L+3 (32%), H-3->L+4 (32%)	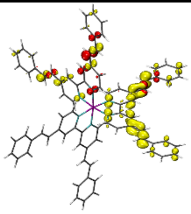
49	328	0.62	H-5->L+3 (15%), H-4->L+4 (31%), H-3->L+3 (33%)	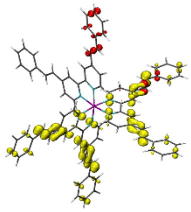
50	327	0.052	H-5->L+3 (29%), H-3->L+5 (51%)	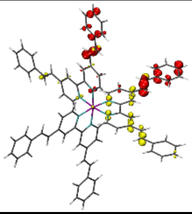

Supplementary Table 9. Calculated singlet electronic transitions (f > 0.01) of **4** in CH_3CN (Isovalue = 0.04).

No.	Wavelength (nm)	*f*	Major contributions	EDDM
1	545	0.089	HOMO->LUMO (95%)	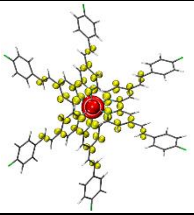
4	520	0.16	H-1->LUMO (91%)	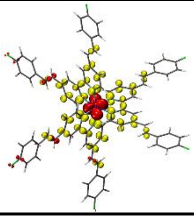
5	520	0.16	H-2->LUMO (91%)	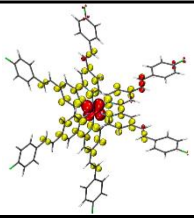
6	517	0.034	H-2->L+2 (45%), H-1->L+1 (47%)	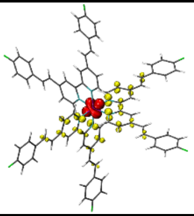
7	499	0.45	H-2->L+1 (13%), H-2->L+2 (33%), H-1->L+1 (32%), H-1->L+2 (13%)	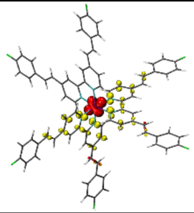
8	499	0.46	H-2->L+1 (33%), H-2->L+2 (14%), H-1->L+1 (13%), H-1->L+2 (33%)	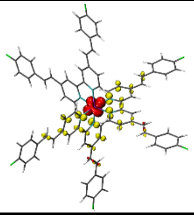
10	408	0.15	HOMO->L+3 (93%)	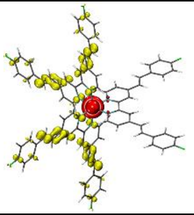

11	408	0.15	HOMO->L+4 (93%)	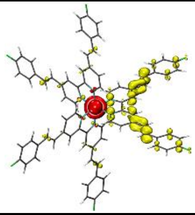
12	403	0.40	H-4->LUMO (23%), H-3->LUMO (64%)	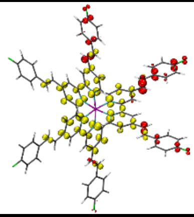
13	403	0.40	H-4->LUMO (64%), H-3->LUMO (23%)	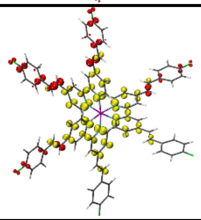
15	398	0.36	H-2->L+3 (11%), H-1->L+4 (11%), H-1->L+5 (68%)	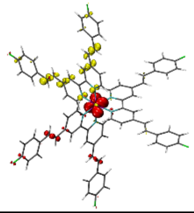
16	398	0.39	H-2->L+5 (55%), H-1->L+3 (33%)	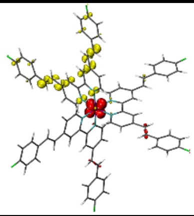
17	398	0.78	H-2->L+4 (51%), H-2->L+5 (10%), H-1->L+3 (28%)	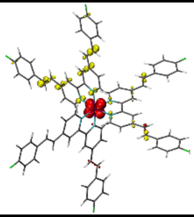
18	398	0.053	H-2->L+4 (37%), H-2->L+5 (13%), H-1->L+3 (25%), H-1->L+5 (14%)	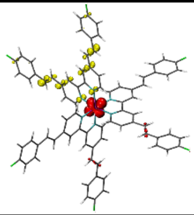
19	398	0.051	H-2->L+3 (30%), H-2->L+5 (16%), H-1->L+4 (30%), H-1->L+5 (13%)	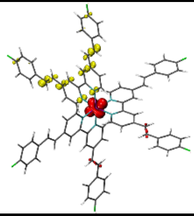

21	393	0.63	H-6->LUMO (20%), H-4->L+2 (31%), H-3->L+1 (34%)	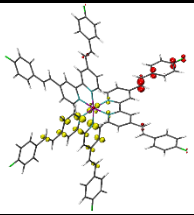
24	383	0.20	H-6->LUMO (23%), HOMO->L+6 (68%)	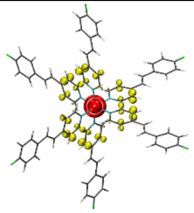
27	380	0.16	H-5->L+1 (74%)	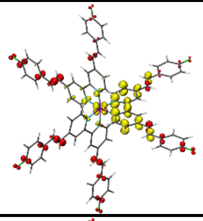
28	380	0.16	H-5->L+2 (74%)	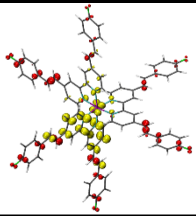
30	372	0.015	H-1->L+6 (84%)	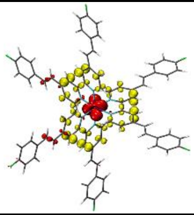
31	372	0.014	H-2->L+6 (83%)	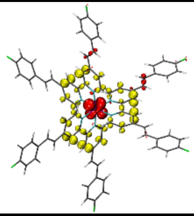
32	371	0.037	H-6->L+1 (71%)	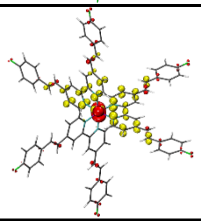
33	371	0.038	H-6->L+2 (70%)	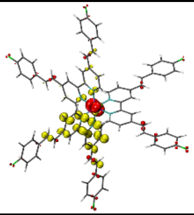

34	365	0.011	H-7->LUMO (76%), H-6->L+2 (12%)	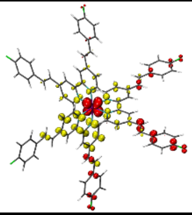
35	364	0.011	H-8->LUMO (77%), H-6->L+1 (11%)	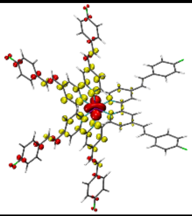
36	356	0.076	HOMO->L+7 (93%)	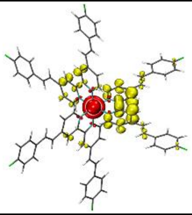
37	356	0.073	HOMO->L+8 (93%)	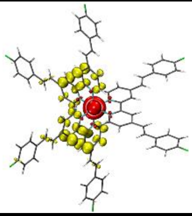
38	355	0.029	H-8->L+1 (41%), H-7->L+2 (42%)	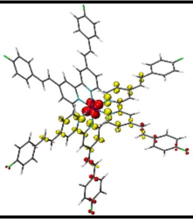
39	354	0.037	H-8->L+2 (42%), H-7->L+1 (47%)	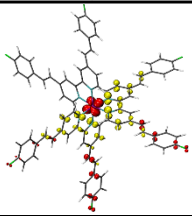
40	354	0.036	H-8->L+1 (45%), H-7->L+2 (44%)	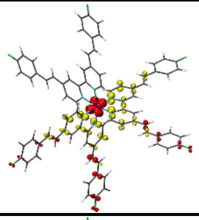
42	347	0.070	H-2->L+7 (34%), H-1->L+7 (14%), H-1->L+8 (35%)	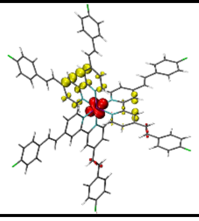

43	347	0.069	H-2->L+7 (12%), H-2->L+8 (25%), H-1->L+7 (44%), H-1->L+8 (10%)	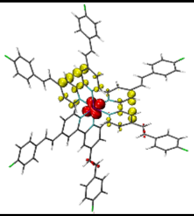
44	346	0.024	H-2->L+8 (59%), H-1->L+7 (33%)	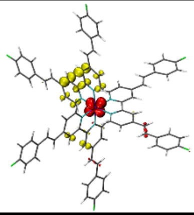
46	331	0.74	H-5->L+4 (15%), H-4->L+4 (31%), H-3->L+3 (34%)	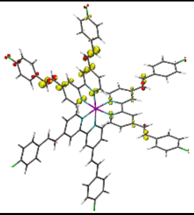
47	331	0.74	H-5->L+3 (16%), H-4->L+3 (32%), H-3->L+4 (32%)	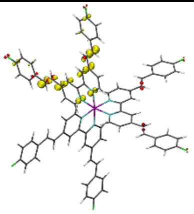
48	330	0.10	H-5->L+3 (30%), H-3->L+5 (46%)	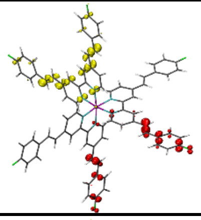
49	330	0.38	H-5->L+5 (26%), H-4->L+4 (26%), H-3->L+3 (24%), H-3->L+5 (10%)	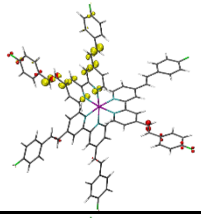
50	330	0.026	H-5->L+4 (37%), H-4->L+5 (55%)	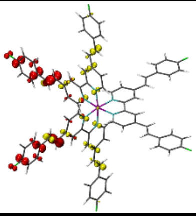

Supplementary Table 10. Calculated singlet electronic transitions (f > 0.01) of **5** in CH_3CN (Isovalue = 0.04).

No.	Wavelength (nm)	f	Major contributions	EDDM
1	551	0.14	HOMO->LUMO (90%)	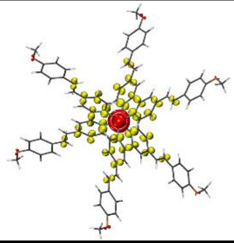
4	534	0.24	H-1->LUMO (93%)	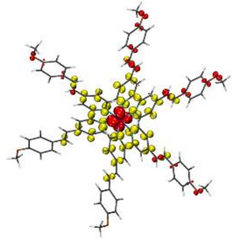
5	533	0.24	H-2->LUMO (93%)	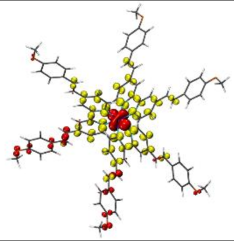
6	527	0.050	H-2->L+2 (43%), H-1->L+1 (45%)	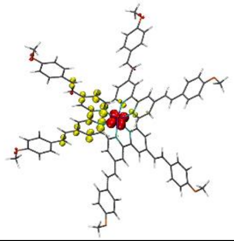
7	512	0.49	H-2->L+1 (42%), H-1->L+2 (42%)	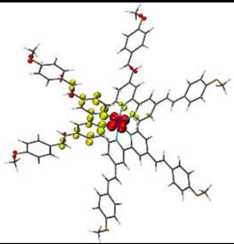
8	512	0.50	H-2->L+2 (43%), H-1->L+1 (41%)	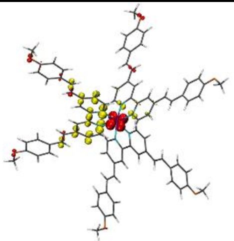

10	442	0.32	H-4->LUMO (26%), H-3->LUMO (65%)	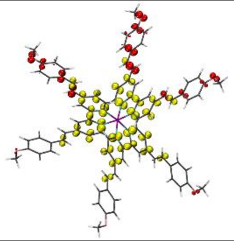
11	442	0.33	H-4->LUMO (65%), H-3->LUMO (26%)	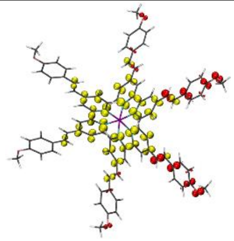
13	428	0.89	H-6->LUMO (15%), H-4->L+1 (30%), H-4->L+2 (11%), H-3->L+1 (12%), H-3->L+2 (29%)	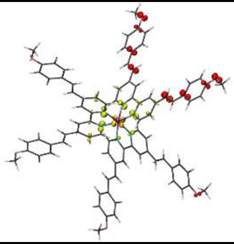
17	415	0.23	H-5->L+1 (80%)	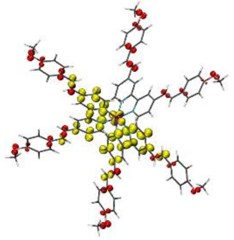
18	415	0.23	H-5->L+2 (81%)	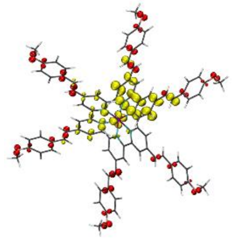
19	414	0.064	H-6->LUMO (73%)	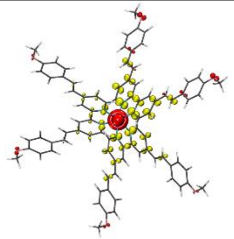
20	411	0.18	HOMO->L+3 (82%)	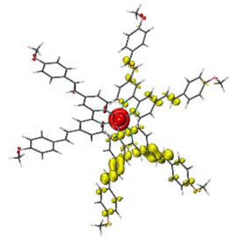

21	411	0.18	HOMO->L+4 (83%)	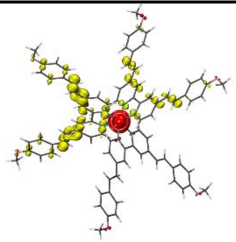
22	406	0.81	H-2->L+3 (25%), H-1->L+3 (33%), H-1->L+4 (21%)	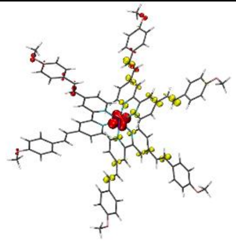
23	406	0.46	H-2->L+3 (17%), H-1->L+4 (14%), H-1->L+5 (43%)	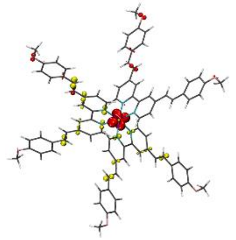
24	406	0.50	H-2->L+4 (25%), H-2->L+5 (40%), H-1->L+4 (10%)	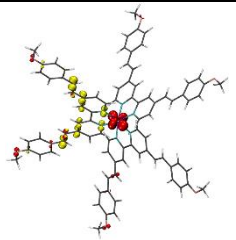
26	404	0.11	H-2->L+4 (37%), H-2->L+5 (11%), H-1->L+3 (21%), H-1->L+5 (17%)	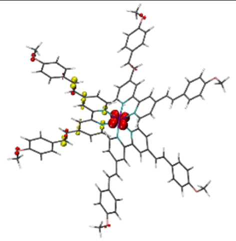
27	403	0.12	H-2->L+3 (27%), H-2->L+5 (18%), H-1->L+4 (30%), H-1->L+5 (12%)	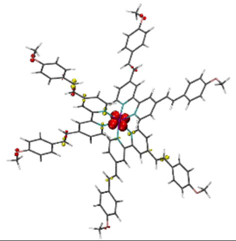
33	388	0.16	HOMO->L+6 (91%)	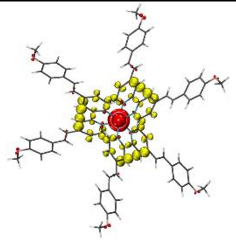

35	381	0.028	H-1->L+6 (91%)	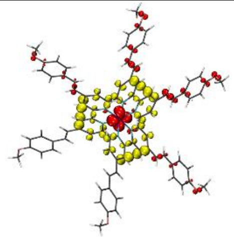
36	381	0.029	H-2->L+6 (91%)	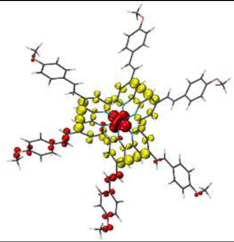
40	361	0.19	HOMO->L+7 (85%)	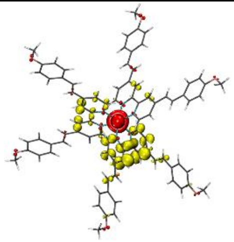
41	361	0.20	HOMO->L+8 (86%)	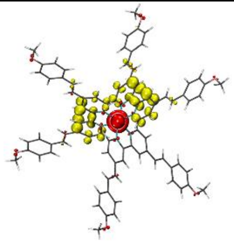
42	355	0.62	H-5->L+4 (13%), H-4->L+3 (35%), H-3->L+4 (35%)	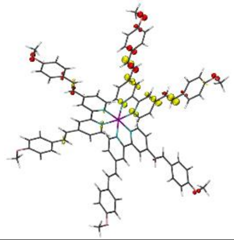
43	355	0.62	H-5->L+3 (14%), H-4->L+4 (33%), H-3->L+3 (36%)	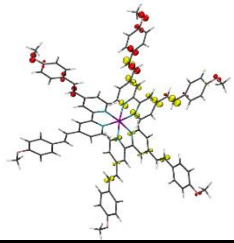
44	354	0.018	H-3->L+5 (17%), H-2->L+8 (27%), H-1->L+7 (35%)	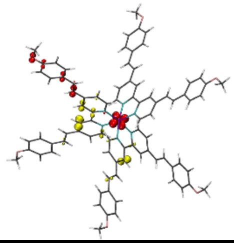

45	354	0.026	H-4->L+5 (16%), H-2->L+7 (32%), H-1->L+8 (32%)	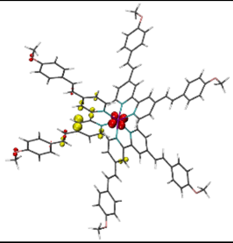
46	354	0.35	H-5->L+5 (18%), H-4->L+4 (25%), H-3->L+3 (22%), H-2->L+8 (13%), H-1->L+7 (13%)	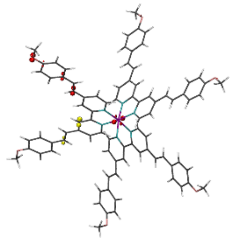
48	354	0.069	H-5->L+3 (31%), H-3->L+5 (38%), H-1->L+7 (11%)	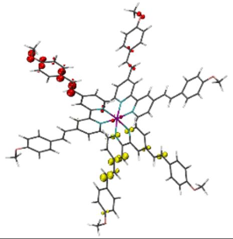
49	353	0.061	H-5->L+4 (32%), H-4->L+5 (37%), H-2->L+7 (13%)	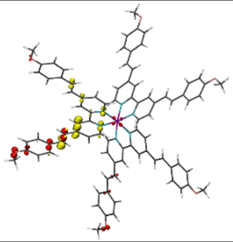
50	353	0.052	H-5->L+5 (13%), H-4->L+4 (10%), H-3->L+3 (10%), H-2->L+8 (29%), H-1->L+7 (23%)	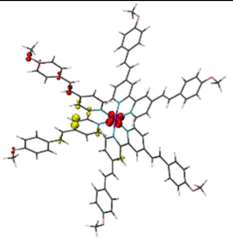

Cartesian coordinates of optimized **1**
Charge = +2, Multiplicity = 1

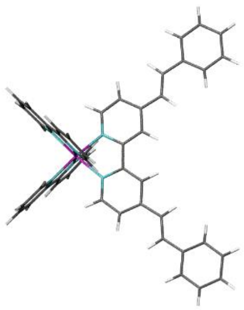

C	-5.430203	-3.400758	-0.420995
C	-5.110045	-2.926177	0.850278
C	-4.115499	-1.962623	0.973319
N	-3.444138	-1.468921	-0.085510
C	-3.752125	-1.926579	-1.333092
C	-4.743548	-2.894391	-1.520442
C	-2.971870	-1.330812	-2.433884
C	-3.125216	-1.690207	-3.776215
C	-2.350205	-1.067943	-4.750308
C	-1.433575	-0.092613	-4.360099
C	-1.324449	0.220678	-3.010019
N	-2.070489	-0.377428	-2.060556
Ru	-1.946127	0.000078	-0.000014
C	-1.324742	-0.220548	3.010065
C	-1.434046	0.092676	4.360141
C	-3.125648	1.690265	3.776121
C	-2.972117	1.330941	2.433785
N	-2.070682	0.377585	2.060539
C	-3.752218	1.926767	1.332918
N	-3.444093	1.469148	0.085364
C	-4.115293	1.962897	-0.973543
C	-5.109821	2.926478	-0.850604
C	-4.743631	2.894618	1.520168
C	-0.383770	-2.660174	0.171590
C	0.730886	-3.478467	0.207684
C	2.079519	-1.509467	0.073025
C	0.920412	-0.737393	0.041730
N	-0.314376	-1.313974	0.091040
C	0.920460	0.737382	-0.041502
N	-0.314289	1.314038	-0.090925
C	-0.383591	2.660248	-0.171415
C	0.731117	3.478477	-0.207341
C	2.079617	1.509387	-0.072653
H	-6.201072	-4.151735	-0.557404
H	-5.616788	-3.288933	1.737382

H	-3.833605	-1.567742	1.941358
H	-4.980907	-3.249999	-2.515244
H	-3.841796	-2.450084	-4.061580
H	-2.462611	-1.341074	-5.794141
H	-0.808554	0.421939	-5.081172
H	-0.627850	0.973778	-2.663713
H	-0.628083	-0.973614	2.663809
H	-0.809116	-0.421885	5.081286
H	-3.842287	2.450107	4.061428
H	-3.833286	1.568027	-1.941553
H	-5.616451	3.289291	-1.737748
H	-4.981099	3.250212	2.514948
H	-1.380149	-3.082362	0.213311
H	0.581683	-4.549727	0.271872
H	3.049723	-1.028969	0.031788
H	-1.379941	3.082500	-0.213217
H	0.581982	4.549754	-0.271411
H	3.049792	1.028830	-0.031359
C	-2.350754	1.067970	4.750284
C	-5.430125	3.401040	0.420649
C	2.020519	2.911589	-0.157395
C	3.263022	3.676041	-0.187082
H	4.166796	3.074768	-0.123946
C	3.359411	5.020827	-0.287409
H	2.445864	5.608589	-0.357405
C	4.586940	5.816868	-0.318703
C	5.878676	5.259370	-0.216306
C	4.474944	7.214571	-0.458032
C	7.007782	6.071853	-0.253954
H	6.003152	4.186629	-0.105150
C	5.606758	8.027675	-0.496302
H	3.486852	7.661043	-0.537653
C	6.878370	7.459010	-0.394493
H	7.994220	5.623952	-0.172960
H	5.495741	9.102806	-0.604958
H	7.763029	8.088707	-0.423212
C	2.020326	-2.911661	0.157815
C	3.262778	-3.676194	0.187648
H	4.166617	-3.074939	0.125275
C	3.359045	-5.021048	0.287178
H	2.445436	-5.608806	0.356390
C	4.586521	-5.817167	0.318552
C	5.878295	-5.259741	0.216235
C	4.474432	-7.214864	0.457857
C	7.007355	-6.072283	0.254027
H	6.002829	-4.187019	0.104962

C	5.606199	-8.028025	0.496285
H	3.486306	-7.661280	0.537358
C	6.877853	-7.459428	0.394611
H	7.993824	-5.624444	0.173082
H	5.495115	-9.103149	0.604947
H	7.762474	-8.089172	0.423435
H	-6.200985	4.152041	0.556960
H	-2.463293	1.341026	5.794118

Cartesian coordinates of optimized **2**
Charge = +2, Multiplicity = 1

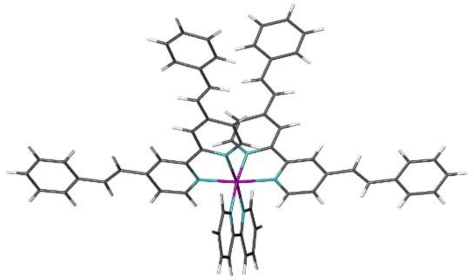

C	0.899820	-5.361835	-2.952380
C	1.029488	-4.078679	-3.482250
C	0.765314	-2.987079	-2.662839
N	0.385028	-3.116599	-1.376606
C	0.251732	-4.368411	-0.851357
C	0.507140	-5.504681	-1.624941
C	-0.174593	-4.418020	0.559722
C	-0.408550	-5.606384	1.258186
C	-0.805321	-5.556875	2.591165
C	-0.960671	-4.313179	3.202003
C	-0.716587	-3.166281	2.455142
N	-0.332178	-3.205384	1.164209
Ru	0.012919	-1.530638	-0.052927
C	-0.267984	0.719369	-2.149462
C	0.151028	1.756779	-2.963033
C	2.368520	1.391915	-2.148447
C	1.887878	0.354495	-1.352638
N	0.566374	0.018029	-1.352218
C	2.740630	-0.455132	-0.458740
N	2.079099	-1.391603	0.279456
C	2.800255	-2.160984	1.122808
C	4.170620	-2.046292	1.273381
C	4.120539	-0.295526	-0.353477
C	-2.760632	-2.120688	-1.278040
C	-4.129827	-2.004208	-1.437024
C	-4.108149	-0.354759	0.293172
C	-2.729187	-0.516552	0.407677
N	-2.053494	-1.399987	-0.381424
C	-1.892328	0.233819	1.366161
N	-0.566227	-0.083885	1.348070
C	0.254311	0.564379	2.202497
C	-0.183768	1.527034	3.094121
C	-2.392152	1.195787	2.241094
H	1.100408	-6.238192	-3.559501
H	1.331392	-3.915906	-4.510717
H	0.852487	-1.973181	-3.032728

H	0.404056	-6.493737	-1.196545
H	-0.285546	-6.563777	0.767563
H	-0.989429	-6.474124	3.140373
H	-1.266983	-4.222467	4.238008
H	-0.823654	-2.180129	2.889290
H	-1.308864	0.421169	-2.125590
H	-0.587041	2.263103	-3.573848
H	3.421846	1.644515	-2.127320
H	2.240732	-2.890616	1.695147
H	4.668853	-2.705600	1.974251
H	4.625093	0.450968	-0.955291
H	-2.190286	-2.810571	-1.887776
H	-4.616510	-2.622850	-2.181714
H	-4.623470	0.353894	0.930502
H	1.299710	0.284550	2.159922
H	0.544548	1.996482	3.744742
H	-3.449726	1.430906	2.237741
C	1.509859	2.130672	-2.981194
C	4.882475	-1.091011	0.520232
C	-1.548864	1.875261	3.137631
C	-2.120747	2.872851	4.036117
H	-3.192074	3.022825	3.925040
C	-1.437281	3.587064	4.958275
H	-0.366218	3.421904	5.060699
C	-1.978443	4.591587	5.874596
C	-3.336684	4.971775	5.898267
C	-1.097856	5.213210	6.782123
C	-3.789140	5.934049	6.795922
H	-4.043346	4.515430	5.211965
C	-1.552137	6.176953	7.681258
H	-0.047575	4.932230	6.777361
C	-2.900428	6.541240	7.691598
H	-4.838848	6.213943	6.798795
H	-0.854663	6.642297	8.371931
H	-3.258781	7.291949	8.390165
C	-4.855133	-1.097683	-0.637833
C	-6.297592	-0.892422	-0.718268
H	-6.690463	-0.159283	-0.017893
C	-7.138598	-1.523676	-1.567842
H	-6.730168	-2.253061	-2.265040
C	-8.586881	-1.341672	-1.672759
C	-9.320030	-0.450229	-0.861727
C	-9.288250	-2.096433	-2.633958
C	-10.697733	-0.323590	-1.011266
H	-8.813008	0.146961	-0.110089
C	-10.668515	-1.968858	-2.783017

H	-8.738811	-2.788371	-3.267510
C	-11.378977	-1.081319	-1.971720
H	-11.245095	0.368197	-0.377180
H	-11.187809	-2.561231	-3.530993
H	-12.454512	-0.978729	-2.084251
C	6.325510	-0.886563	0.592236
H	6.706052	-0.108733	-0.065472
C	7.180801	-1.568204	1.386829
H	6.784677	-2.341669	2.042384
C	8.630135	-1.388605	1.481021
C	9.346609	-2.193252	2.389143
C	9.349997	-0.452181	0.709756
C	10.728546	-2.069959	2.525850
C	10.729401	-0.330003	0.846767
H	8.831357	0.183181	-0.001571
C	11.425662	-1.137167	1.754734
H	11.259576	-2.701023	3.232818
H	11.266362	0.396784	0.243697
H	12.502466	-1.037879	1.857666
H	8.807632	-2.920557	2.991285
C	2.060788	3.211417	-3.792256
H	3.135428	3.347698	-3.696871
C	1.353817	4.021041	-4.612171
H	0.277612	3.877518	-4.690297
C	1.873599	5.112572	-5.436792
C	0.961937	5.864159	-6.204567
C	3.240832	5.453091	-5.504893
C	1.394437	6.917428	-7.009051
C	3.671606	6.504405	-6.308422
H	3.972049	4.893493	-4.929707
C	2.751796	7.241876	-7.063933
H	0.673047	7.483153	-7.591807
H	4.728827	6.751288	-6.348128
H	3.093324	8.061394	-7.689804
H	-0.095579	5.614958	-6.164446

Cartesian coordinates of optimized **3**
Charge = +2, Multiplicity = 1

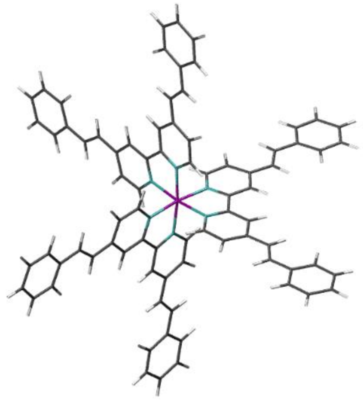

C	2.582780	-2.216714	2.790143
C	1.493083	-1.662727	2.142715
N	1.600960	-0.860115	1.062187
C	2.849977	-0.588317	0.587003
C	3.985491	-1.117638	1.196549
C	2.890737	0.297099	-0.594486
C	4.070112	0.711127	-1.209870
C	4.049124	1.556980	-2.332566
C	2.776192	1.952704	-2.790485
C	1.639971	1.508745	-2.138237
N	1.672675	0.694780	-1.061176
Ru	0.007825	-0.003845	0.002438
C	-2.181081	-0.434092	2.138906
C	-3.212907	-1.089817	2.786126
C	-2.959210	-2.871416	1.212547
C	-1.925486	-2.163614	0.602984
N	-1.535281	-0.942235	1.067415
C	-1.173111	-2.657111	-0.568369
N	-0.216861	-1.807529	-1.041178
C	0.508392	-2.199446	-2.110777
C	0.326024	-3.412556	-2.750021
C	-1.401829	-3.892981	-1.169491
C	0.721353	2.132564	2.116217
C	0.660842	3.358991	2.753253
C	-1.011498	4.006185	1.172384
C	-0.907349	2.752399	0.573932
N	-0.039521	1.813049	1.047298
C	-1.706193	2.333498	-0.595511
N	-1.438679	1.079321	-1.059314
C	-2.131904	0.637238	-2.130579
C	-3.093575	1.391739	-2.778404

C	-2.666151	3.138909	-1.204172
H	2.403698	-2.849643	3.651241
H	0.485761	-1.854072	2.491491
H	4.967687	-0.887469	0.800958
H	5.026013	0.381556	-0.820614
H	2.656903	2.602550	-3.649384
H	0.655230	1.802456	-2.480535
H	-1.841494	0.535967	2.480618
H	-3.674871	-0.607958	3.639711
H	-3.252984	-3.839429	0.824196
H	1.257682	-1.498561	-2.457606
H	0.952744	-3.645289	-3.602824
H	-2.166234	-4.551752	-0.775026
H	1.397232	1.361214	2.464130
H	1.308700	3.530246	3.604820
H	-1.706111	4.737617	0.776662
H	-1.890710	-0.362004	-2.471716
H	-3.600506	0.957969	-3.632288
H	-2.863854	4.130428	-0.814836
C	-3.639184	-2.352898	2.328524
C	-6.540659	-3.515676	4.627681
C	-7.187117	-2.954839	5.747080
C	-6.977072	-4.776407	4.169380
C	-8.229329	-3.623910	6.387250
H	-6.863675	-1.983721	6.113705
C	-8.017451	-5.443463	4.808996
H	-6.502719	-5.238726	3.309210
C	-8.648795	-4.871612	5.920343
H	-8.712694	-3.171755	7.248636
H	-8.339514	-6.413595	4.441025
H	-9.460532	-5.396490	6.415907
C	-4.717130	-3.130866	2.930500
C	-5.453952	-2.761137	4.001864
H	-4.913624	-4.084070	2.445626
H	-5.244970	-1.804395	4.477139
C	-0.655808	-4.310779	-2.285409
C	-0.933914	-5.614021	-2.880236
H	-1.722400	-6.178263	-2.387934
C	-0.305475	-6.141007	-3.954898
H	0.480165	-5.563176	-4.438430
C	-0.560043	-7.441713	-4.575531
C	-1.531524	-8.350828	-4.106770
C	0.204430	-7.807204	-5.701348
C	-1.725858	-9.573096	-4.743028
H	-2.138276	-8.103106	-3.241222
C	0.009016	-9.031955	-6.338086

H	0.957909	-7.118603	-6.075747
C	-0.957657	-9.920085	-5.861160
H	-2.478950	-10.260065	-4.367295
H	0.610339	-9.291926	-7.204616
H	-1.113387	-10.875424	-6.354169
C	-3.393448	2.690626	-2.320587
C	-4.388142	3.572436	-2.922754
H	-4.491091	4.539241	-2.435718
C	-5.153496	3.280602	-3.998133
H	-5.037337	2.309768	-4.476572
C	-6.156957	4.141228	-4.625932
C	-6.475113	5.433884	-4.159254
C	-6.842660	3.654934	-5.756746
C	-7.439862	6.203470	-4.802279
H	-5.967863	5.840533	-3.289727
C	-7.808888	4.426766	-6.400395
H	-6.609840	2.660592	-6.129815
C	-8.111361	5.704845	-5.925322
H	-7.671208	7.196743	-4.427908
H	-8.324328	4.030863	-7.270820
H	-8.863646	6.309678	-6.423544
C	-0.226921	4.349616	2.287235
C	-0.373340	5.675357	2.879247
H	-1.095727	6.317013	2.380478
C	0.297170	6.136144	3.958828
H	1.015024	5.481200	4.449413
C	0.173000	7.457380	4.575880
C	-0.699194	8.459134	4.100777
C	0.965840	7.746420	5.704293
C	-0.770267	9.696928	4.732656
H	-1.325347	8.271597	3.233903
C	0.893870	8.986784	6.336736
H	1.643589	6.985712	6.083796
C	0.024967	9.967444	5.852987
H	-1.448035	10.455796	4.351850
H	1.514967	9.186840	7.205233
H	-0.034354	10.935332	6.342547
C	5.311491	1.964423	-2.940799
H	6.198169	1.554438	-2.463183
C	5.445093	2.782507	-4.008681
H	4.548519	3.187017	-4.474969
C	6.693735	3.210391	-4.640839
C	6.620090	4.074752	-5.751219
C	7.969541	2.803382	-4.197447
C	7.773517	4.517999	-6.396816
C	9.120245	3.246196	-4.842617

H	8.064450	2.137969	-3.344854
C	9.028852	4.105200	-5.944667
H	7.691936	5.184172	-7.250993
H	10.093914	2.921633	-4.486377
H	9.930217	4.447991	-6.444683
H	5.644772	4.398401	-6.106370
C	3.886229	-1.952576	2.323368
C	5.104967	-2.482268	2.926559
H	6.024283	-2.179745	2.430955
C	5.163571	-3.286956	4.011270
H	4.234948	-3.580133	4.497756
C	6.366912	-3.834923	4.638809
C	6.214976	-4.661348	5.769969
C	7.673248	-3.580380	4.171153
C	7.321927	-5.214488	6.412123
C	8.777559	-4.132857	4.812869
H	7.828720	-2.948184	3.302351
C	8.608347	-4.952218	5.935685
H	7.179957	-5.848823	7.282490
H	9.775765	-3.925242	4.437683
H	9.473765	-5.381014	6.432850
H	5.215316	-4.868152	6.144083

Cartesian coordinates of optimized **4**
Charge = +2, Multiplicity = 1

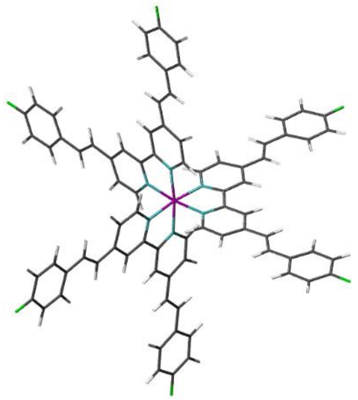

C	2.598103	2.195863	-2.782288
C	1.503818	1.653047	-2.133181
N	1.605230	0.844509	-1.056354
C	2.852368	0.555272	-0.586634
C	3.992274	1.072498	-1.198212
C	2.886411	-0.333368	0.592676
C	4.062631	-0.762838	1.203416
C	4.034904	-1.607513	2.327002
C	2.758609	-1.986848	2.789528
C	1.625907	-1.528663	2.141135
N	1.664981	-0.715746	1.063532
Ru	0.005229	0.001260	0.003029
C	-2.178412	0.457246	-2.132718
C	-3.201483	1.125582	-2.780892
C	-2.930994	2.901161	-1.203361
C	-1.905675	2.181366	-0.593813
N	-1.528431	0.956437	-1.059481
C	-1.148712	2.665584	0.578448
N	-0.202096	1.804973	1.050792
C	0.526692	2.187804	2.121287
C	0.357943	3.402588	2.761039
C	-1.363950	3.903456	1.180304
C	0.696570	-2.136084	-2.116668
C	0.620849	-3.358450	-2.759678
C	-1.058929	-3.992824	-1.181535
C	-0.938507	-2.743767	-0.576293
N	-0.060096	-1.812346	-1.046061
C	-1.730727	-2.321519	0.596644
N	-1.451294	-1.070791	1.062840
C	-2.139591	-0.624385	2.135467
C	-3.106954	-1.371581	2.783022
C	-2.695412	-3.120676	1.206279

H	2.423891	2.834114	-3.640505
H	0.497819	1.858810	-2.477708
H	4.972724	0.828193	-0.806999
H	5.021447	-0.445628	0.810970
H	2.633735	-2.635800	3.648303
H	0.638745	-1.809523	2.487022
H	-1.849521	-0.516299	-2.474932
H	-3.666992	0.650150	-3.636174
H	-3.214669	3.871478	-0.813557
H	1.267268	1.478008	2.468748
H	0.986871	3.627435	3.614354
H	-2.120641	4.571189	0.785962
H	1.381615	-1.371370	-2.461447
H	1.265371	-3.532678	-3.613189
H	-1.763291	-4.716794	-0.789393
H	-1.888862	0.371951	2.478282
H	-3.608990	-0.934529	3.638181
H	-2.900585	-4.110711	0.816598
C	-3.614512	2.392668	-2.321785
C	-6.491962	3.593934	-4.628999
C	-7.135641	3.047624	-5.757720
C	-6.921631	4.855997	-4.166059
C	-8.166116	3.723226	-6.406989
H	-6.820983	2.076995	-6.131348
C	-7.948748	5.542961	-4.802772
H	-6.452596	5.310432	-3.299454
C	-8.555453	4.963720	-5.915642
H	-8.662232	3.304253	-7.275881
H	-8.283736	6.513211	-4.451646
C	-4.681444	3.184600	-2.924501
C	-5.417611	2.826564	-4.000447
H	-4.868718	4.138391	-2.436959
H	-5.216480	1.869655	-4.478362
C	-0.613195	4.312440	2.296451
C	-0.876069	5.618651	2.891005
H	-1.658905	6.191079	2.399152
C	-0.239595	6.139407	3.964118
H	0.541388	5.554066	4.445787
C	-0.478068	7.441948	4.584339
C	-1.447083	8.358549	4.123309
C	0.298980	7.805405	5.702733
C	-1.632142	9.584691	4.751461
H	-2.066006	8.116244	3.265572
C	0.125962	9.030041	6.343211
H	1.050963	7.114836	6.074634
C	-0.840436	9.900924	5.853858

H	-2.375376	10.293445	4.401965
H	0.723197	9.311248	7.203959
C	-3.417581	-2.667746	2.324403
C	-4.418123	-3.541556	2.927873
H	-4.526826	-4.508998	2.443470
C	-5.183138	-3.240313	4.001082
H	-5.061664	-2.268150	4.474977
C	-6.192887	-4.089749	4.631118
C	-6.513393	-5.386768	4.176541
C	-6.885293	-3.590532	5.752790
C	-7.482685	-6.152236	4.814352
H	-6.003433	-5.807125	3.315883
C	-7.859064	-4.344780	6.402979
H	-6.654604	-2.594198	6.119991
C	-8.141106	-5.617158	5.919917
H	-7.733525	-7.149830	4.469713
H	-8.392653	-3.962036	7.266378
C	-0.278814	-4.340585	-2.298329
C	-0.441151	-5.661221	-2.897003
H	-1.177949	-6.293587	-2.407440
C	0.233544	-6.128168	-3.971553
H	0.967416	-5.482986	-4.450875
C	0.095869	-7.443190	-4.595936
C	-0.800008	-8.432723	-4.137850
C	0.898547	-7.742675	-5.715223
C	-0.890469	-9.667602	-4.769623
H	-1.435471	-8.240983	-3.279414
C	0.819949	-8.975075	-6.359341
H	1.595348	-6.995453	-6.085077
C	-0.076808	-9.918954	-5.872738
H	-1.577171	-10.432277	-4.422376
H	1.436956	-9.207074	-7.220811
C	5.293891	-2.030000	2.931312
H	6.184165	-1.642036	2.442092
C	5.419546	-2.835310	4.009974
H	4.518821	-3.215228	4.488436
C	6.662693	-3.277868	4.639996
C	6.581748	-4.124111	5.764345
C	7.944023	-2.903087	4.182423
C	7.724750	-4.584162	6.413783
C	9.094432	-3.353612	4.819481
H	8.049169	-2.252818	3.320129
C	8.965362	-4.188936	5.927477
H	7.664090	-5.235476	7.279089
H	10.082129	-3.069095	4.472478
H	5.605376	-4.425295	6.134043

C	3.899851	1.913205	-2.321357
C	5.123052	2.429010	-2.926781
H	6.040321	2.109035	-2.438261
C	5.187540	3.240649	-4.006094
H	4.260832	3.551329	-4.484873
C	6.393976	3.774237	-4.636986
C	6.249897	4.607118	-5.764969
C	7.699679	3.500143	-4.176916
C	7.355151	5.150032	-6.415762
C	8.813027	4.034279	-4.815196
H	7.853166	2.863735	-3.311516
C	8.621787	4.852486	-5.926973
H	7.245953	5.791080	-7.283970
H	9.819198	3.827439	-4.466316
H	5.253742	4.831443	-6.136483
F	-0.164490	-11.117885	-6.486284
F	-9.082246	-6.360236	6.539308
F	-9.552819	5.630511	-6.533874
F	-1.019976	11.091365	6.463930
F	9.702636	5.371212	-6.546840
F	10.082166	-4.626523	6.546213

Cartesian coordinates of optimized **5**
Charge = +2, Multiplicity = 1

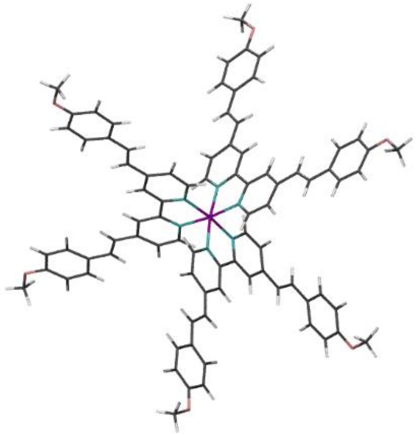

C	-2.292554	2.511109	2.782722
C	-1.277737	1.830116	2.135529
N	-1.480703	1.040402	1.058727
C	-2.754822	0.915876	0.587769
C	-3.818032	1.578480	1.196287
C	-2.903111	0.036197	-0.589867
C	-4.124535	-0.238885	-1.199968
C	-4.208486	-1.083813	-2.321897
C	-2.990266	-1.626966	-2.781106
C	-1.808358	-1.317827	-2.133099
N	-1.740814	-0.502928	-1.057991
Ru	-0.001941	-0.002788	0.000631
C	2.224966	0.178311	2.134403
C	3.321931	0.715265	2.783031
C	3.274392	2.509089	1.203950
C	2.169350	1.920722	0.593615
N	1.641649	0.752606	1.060024
C	1.480735	2.492838	-0.581655
N	0.432743	1.757096	-1.051799
C	-0.239391	2.226186	-2.125424
C	0.083497	3.406317	-2.770011
C	1.852660	3.690265	-1.187938
C	-0.958730	-2.031795	2.124919
C	-1.042308	-3.253223	2.767927
C	0.540230	-4.100114	1.189265
C	0.582383	-2.846503	0.583863
N	-0.167669	-1.808303	1.053188
C	1.422202	-2.530716	-0.589898
N	1.304640	-1.254661	-1.057381
C	2.045379	-0.902324	-2.130505
C	2.909224	-1.767511	-2.776657
C	2.276673	-3.447183	-1.198017

H	-2.037637	3.121650	3.641052
H	-0.253858	1.903733	2.481297
H	-4.821064	1.464643	0.802403
H	-5.034188	0.200910	-0.808486
H	-2.950455	-2.289693	-3.637605
H	-0.865824	-1.726314	-2.476855
H	1.777916	-0.746928	2.477077
H	3.723853	0.186612	3.639424
H	3.675606	3.436768	0.813185
H	-1.064211	1.614995	-2.471124
H	-0.510217	3.704977	-3.626062
H	2.688207	4.257027	-0.794339
H	-1.537913	-1.183974	2.469995
H	-1.703268	-3.342067	3.622091
H	1.145331	-4.909161	0.797391
H	1.924350	0.117752	-2.474123
H	3.463421	-1.399051	-3.631928
H	2.353244	-4.454905	-0.807084
C	3.890319	1.922299	2.324736
C	6.887579	2.755766	4.636257
C	7.470777	2.133406	5.755108
C	7.467080	3.966345	4.189387
C	8.575948	2.672762	6.411276
H	7.047980	1.201487	6.122329
C	8.564076	4.514323	4.829863
H	7.053574	4.485423	3.330229
C	9.131702	3.873799	5.949183
H	8.990370	2.156953	7.269290
H	9.006426	5.443720	4.484723
C	5.044051	2.576107	2.927765
C	5.732203	2.129676	4.005397
H	5.348134	3.500241	2.441736
H	5.414595	1.202563	4.479867
C	1.162484	4.188925	-2.308052
C	1.590254	5.446275	-2.906550
H	2.441721	5.915078	-2.418804
C	1.023317	6.043247	-3.981834
H	0.171158	5.560462	-4.457321
C	1.422997	7.297091	-4.608617
C	2.505679	8.089688	-4.160682
C	0.701045	7.758638	-5.724455
C	2.838984	9.271856	-4.797314
H	3.094262	7.776561	-3.303835
C	1.024294	8.947352	-6.376619
H	-0.136631	7.171194	-6.092407
C	2.101882	9.714906	-5.913432

H	3.670571	9.878030	-4.451490
H	0.438917	9.262162	-7.232325
C	3.052457	-3.093569	-2.317268
C	3.931662	-4.087707	-2.917925
H	3.914695	-5.061394	-2.433741
C	4.733238	-3.887852	-3.991068
H	4.737401	-2.906753	-4.463170
C	5.625358	-4.854384	-4.619204
C	5.779969	-6.187401	-4.171812
C	6.383113	-4.454060	-5.734959
C	6.643198	-7.060822	-4.808874
H	5.217476	-6.544437	-3.314644
C	7.257041	-5.321904	-6.387531
H	6.286075	-3.435401	-6.102432
C	7.392263	-6.638161	-5.924948
H	6.759884	-8.083411	-4.463512
H	7.820068	-4.968040	-7.242959
C	-0.278410	-4.345837	2.306926
C	-0.289154	-5.674536	2.904030
H	0.364007	-6.395920	2.418590
C	-1.026041	-6.053968	3.975078
H	-1.674289	-5.318719	4.449185
C	-1.063894	-7.371102	4.598496
C	-0.306172	-8.476655	4.146121
C	-1.896835	-7.570699	5.714588
C	-0.383057	-9.704575	4.778745
H	0.351183	-8.373881	3.288288
C	-1.985290	-8.801434	6.362851
H	-2.491904	-6.740011	6.085934
C	-1.223811	-9.881490	5.895521
H	0.199821	-10.551126	4.429192
H	-2.640998	-8.907026	7.218898
C	-5.509577	-1.340360	-2.924832
H	-6.343002	-0.840831	-2.436374
C	-5.739576	-2.123491	-4.005544
H	-4.893479	-2.615058	-4.483050
C	-7.023701	-2.402382	-4.636456
C	-7.058604	-3.246929	-5.761088
C	-8.253752	-1.870114	-4.184002
C	-8.248077	-3.558320	-6.417542
C	-9.442680	-2.169716	-4.824778
H	-8.279847	-1.213397	-3.319937
C	-9.453852	-3.017709	-5.950010
H	-8.225100	-4.213641	-7.280035
H	-10.385037	-1.759403	-4.475238
H	-6.129354	-3.671826	-6.132639

C	-3.620709	2.401825	2.320044
C	-4.765570	3.072456	2.921386
H	-5.716473	2.874750	2.432071
C	-4.725920	3.889034	4.001034
H	-3.765345	4.076332	4.478179
C	-5.847284	4.575705	4.630165
C	-5.602270	5.391888	5.749594
C	-7.184499	4.472029	4.180545
C	-6.623191	6.079905	6.403165
C	-8.208805	5.148487	4.818579
H	-7.425512	3.853837	3.321252
C	-7.940095	5.961070	5.937888
H	-6.385656	6.697045	7.261475
H	-9.234205	5.067030	4.471472
H	-4.584603	5.491210	6.119161
O	10.203589	4.495736	6.502434
O	2.512454	10.886013	-6.462713
O	-9.015524	6.579460	6.488109
O	-10.671840	-3.246063	-6.503348
O	-1.224350	-11.124022	6.441351
O	8.207868	-7.573060	-6.474833
C	-10.752071	-4.094822	-7.648656
H	-10.396533	-5.105955	-7.419100
H	-10.176230	-3.685652	-8.486825
C	-2.052656	-11.368850	7.578223
H	-3.110588	-11.208472	7.340485
H	-1.766293	-10.730975	8.422402
C	-8.811844	7.417679	7.625803
H	-8.144729	8.254283	7.388061
H	-8.401034	6.849798	8.468567
C	1.808137	11.390993	-7.597352
H	0.757575	11.589943	-7.356084
H	1.864338	10.694243	-8.441676
C	10.824766	3.900209	7.641718
H	11.217148	2.904525	7.404832
H	10.125255	3.827656	8.482523
C	8.993212	-7.209385	-7.610349
H	9.686118	-6.394975	-7.369285
H	8.358335	-6.912530	-8.453148
H	2.304229	12.325296	-7.862470
H	11.648994	4.562135	7.909731
H	9.559363	-8.102355	-7.877533
H	-1.893069	-12.415046	7.841527
H	-11.808594	-4.132289	-7.916397
H	-9.797411	7.801748	7.891247

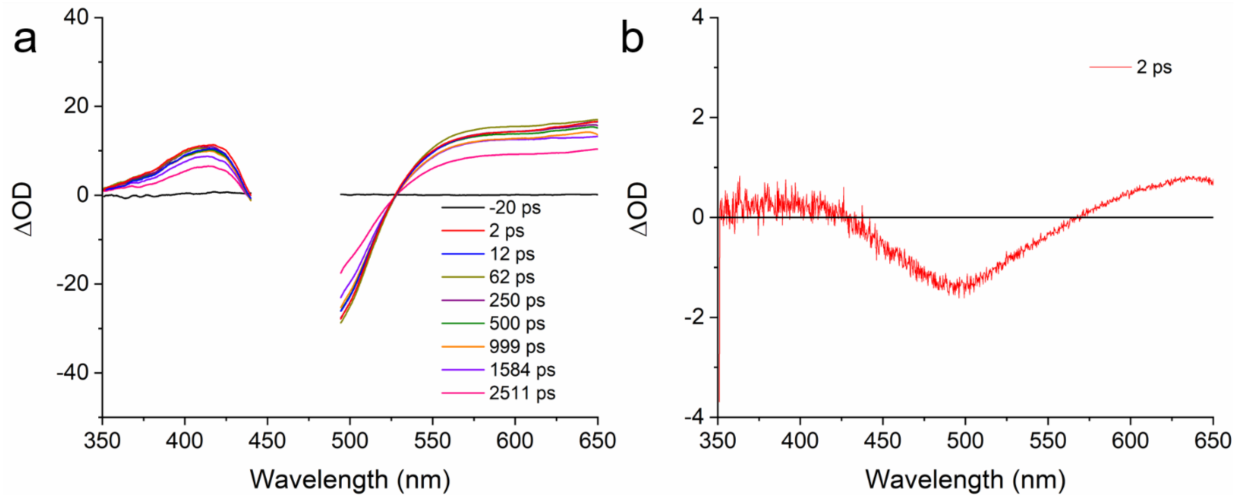

Supplementary Figure 20. Transient absorption spectra of **2** under irradiation of (a) 480 nm and (b) 800 nm.

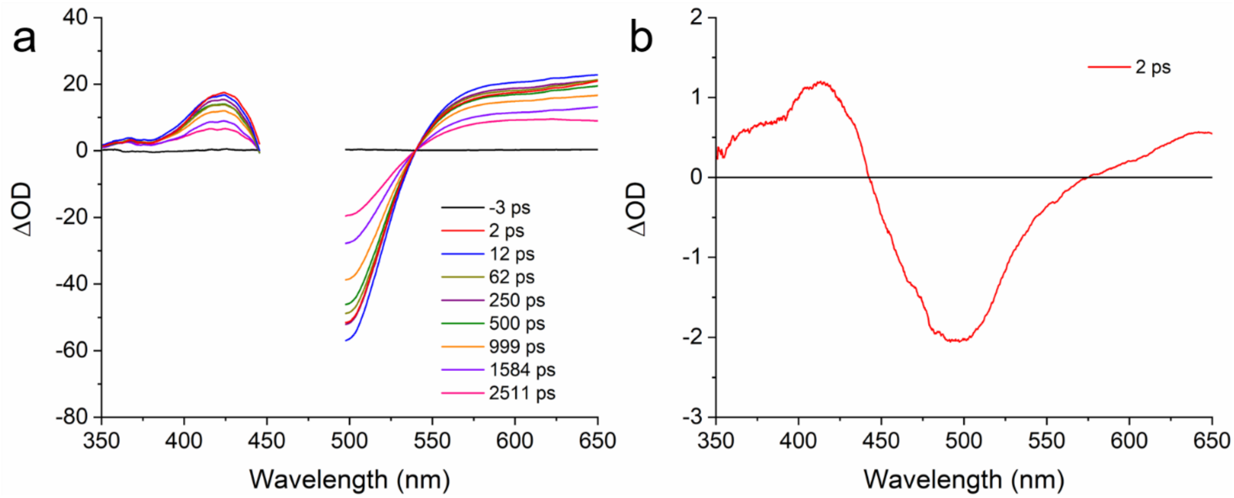

Supplementary Figure 21. Transient absorption spectra of **3** under irradiation of (a) 480 nm and (b) 800 nm.

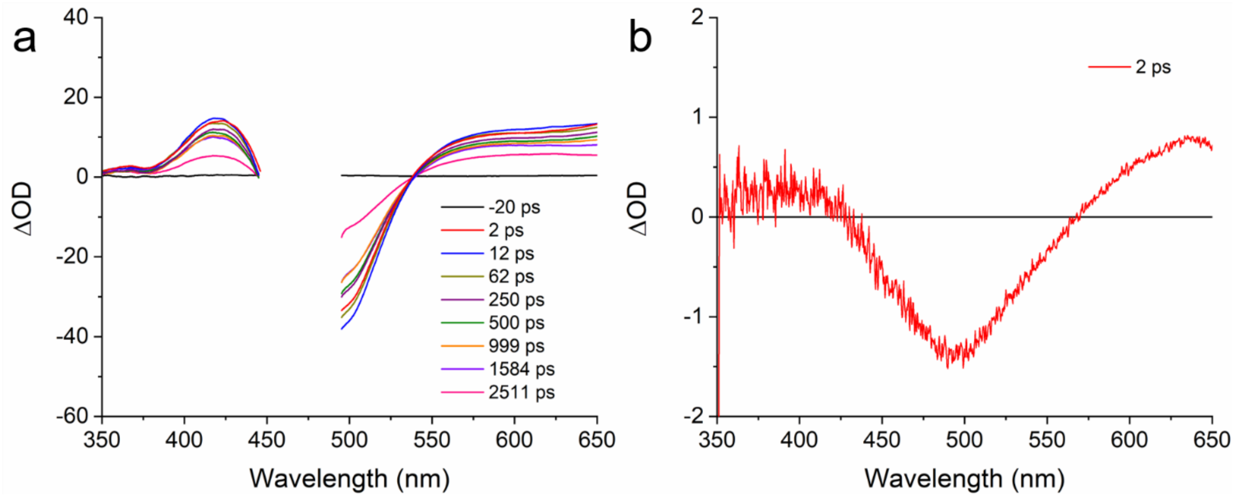

Supplementary Figure 22. Transient absorption spectra of **4** under irradiation of (a) 480 nm and (b) 800 nm.

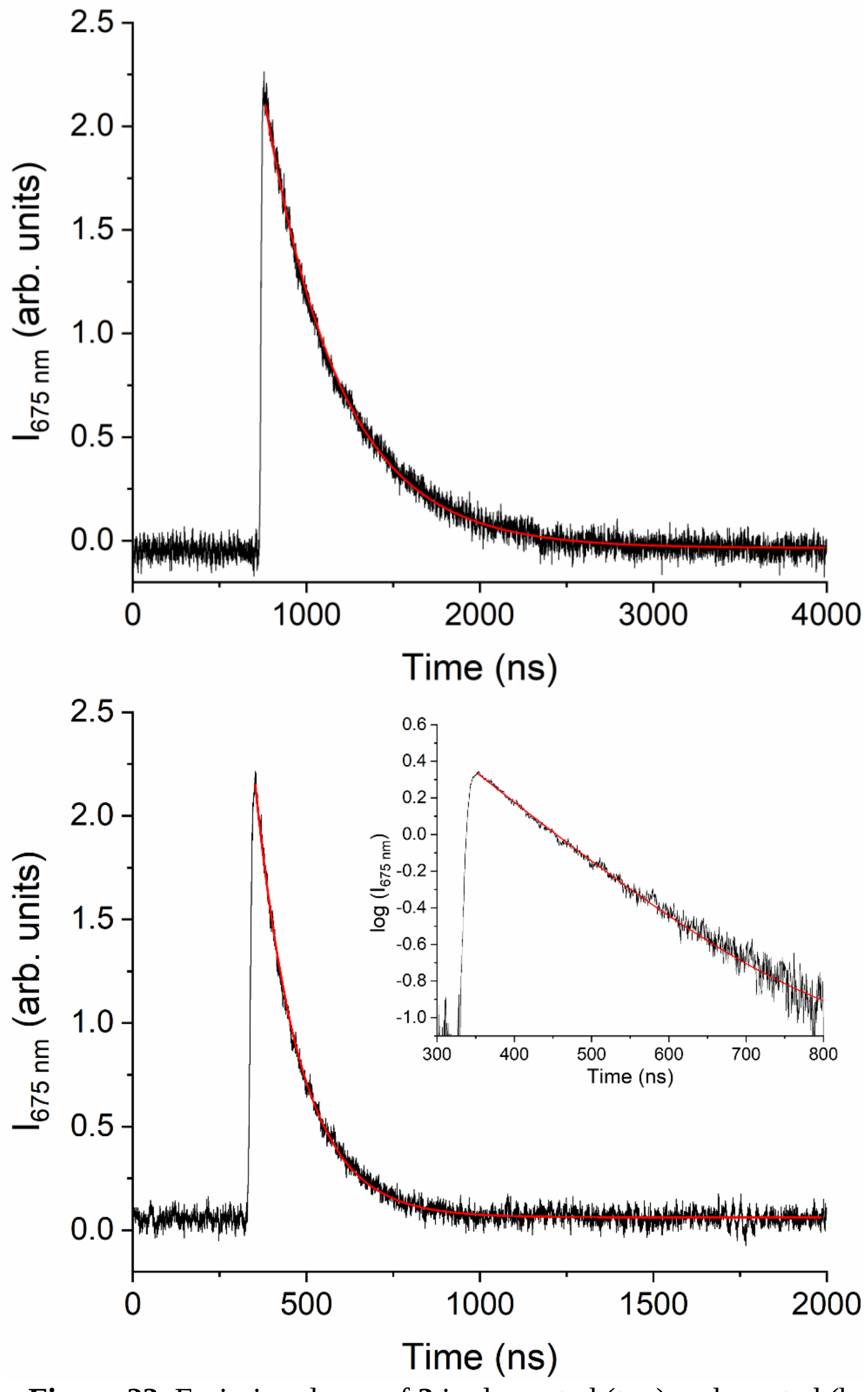

Supplementary Figure 23. Emission decay of **3** in deaerated (top) and aerated (bottom) CH_3CN. Insets show the intensity plotted on a log scale (excited at 480 nm and decay trace taken at 670 nm).

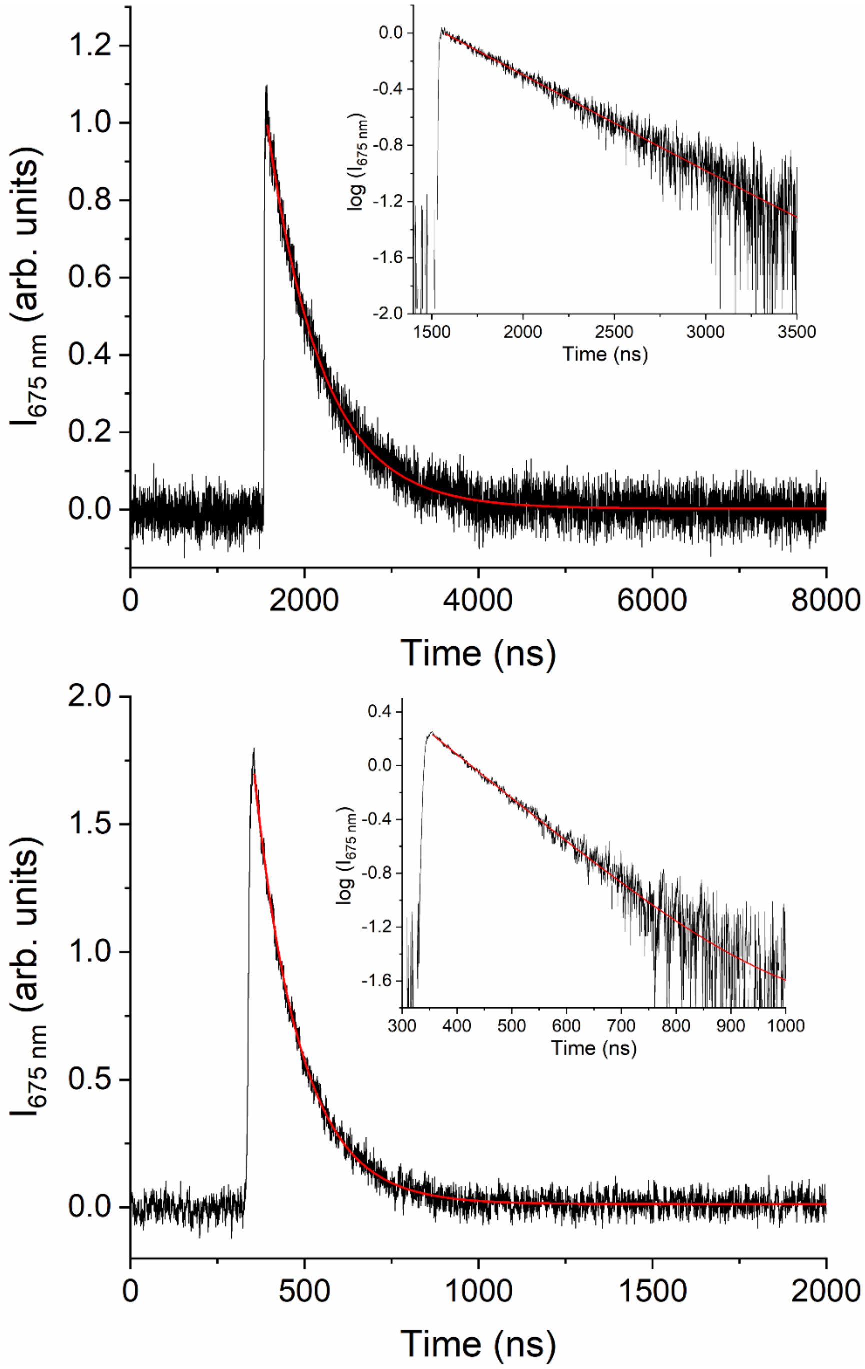

Supplementary Figure 24. Emission decay of **4** in deaerated (top) and aerated (bottom) CH_3CN. Insets show the intensity plotted on a log scale (excited at 480 nm and decay trace taken at 670 nm).

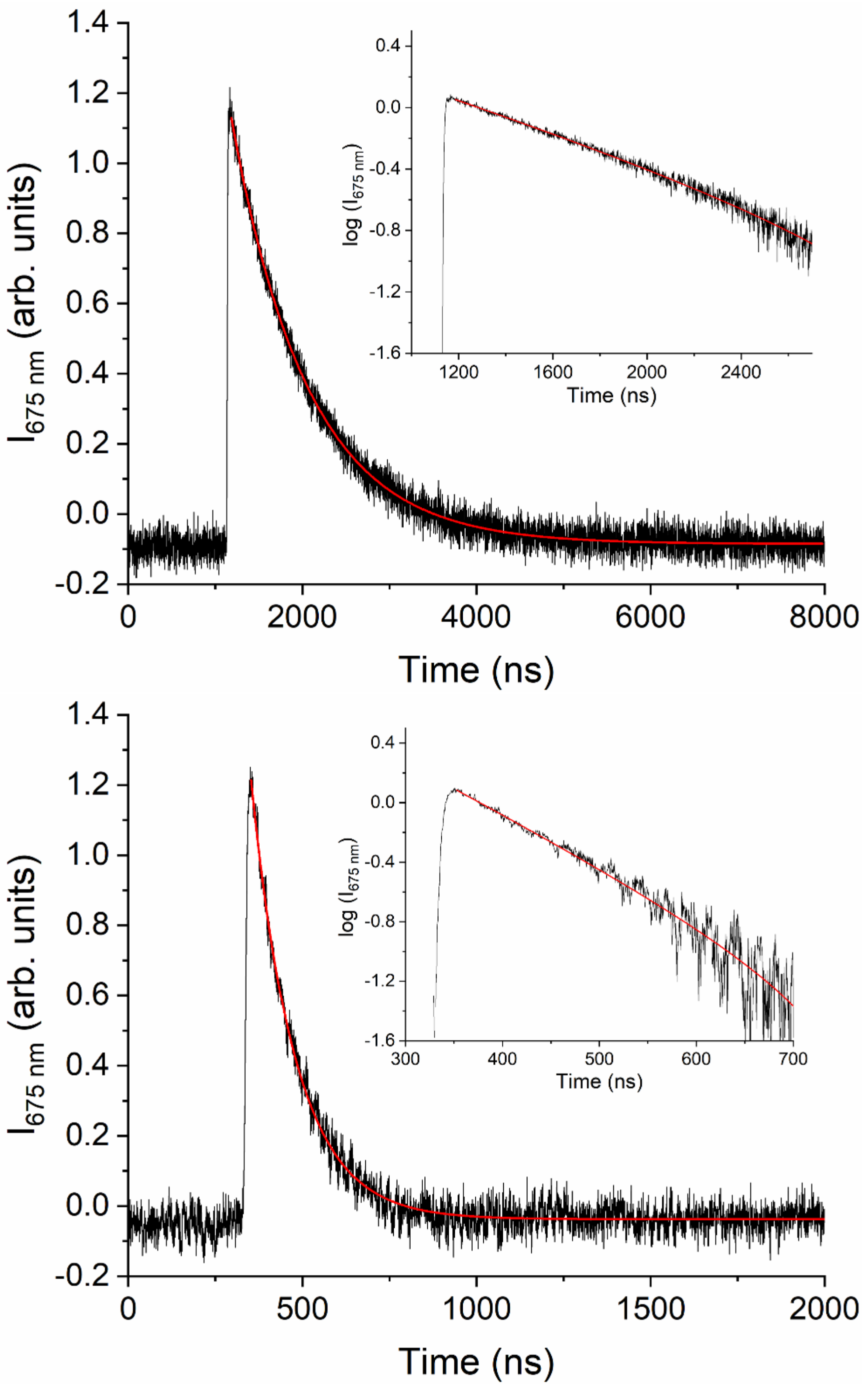

Supplementary Figure 25. Emission decay of **5** in deaerated (top) and aerated (bottom) CH_3CN. Insets show the intensity plotted on a log scale (excited at 480 nm and decay trace taken at 670 nm).

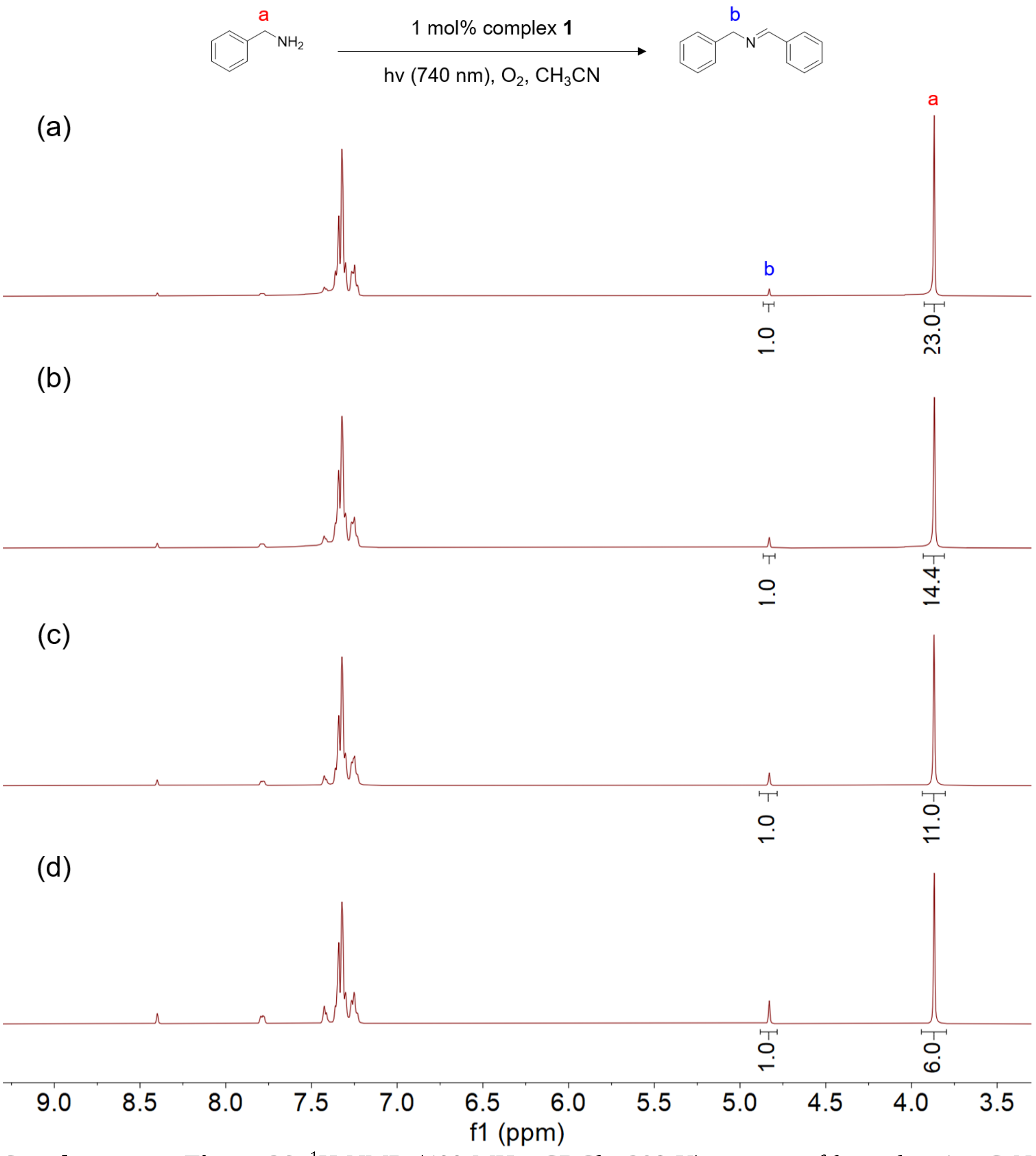

Supplementary Figure 26. ^{1}H NMR (400 MHz, $CDCl_3$, 298 K) spectra of benzyl amine C-N coupling using **1** as the photosensitizer with different irradiation time: (a) 30 min; (b) 1 h; (c) 2 h; (d) 4 h. Condition: 50 mM benzyl amine, 1 mol% **1**, 5 mL CH_3CN, O_2, 740 nm LEDs, room temperature.

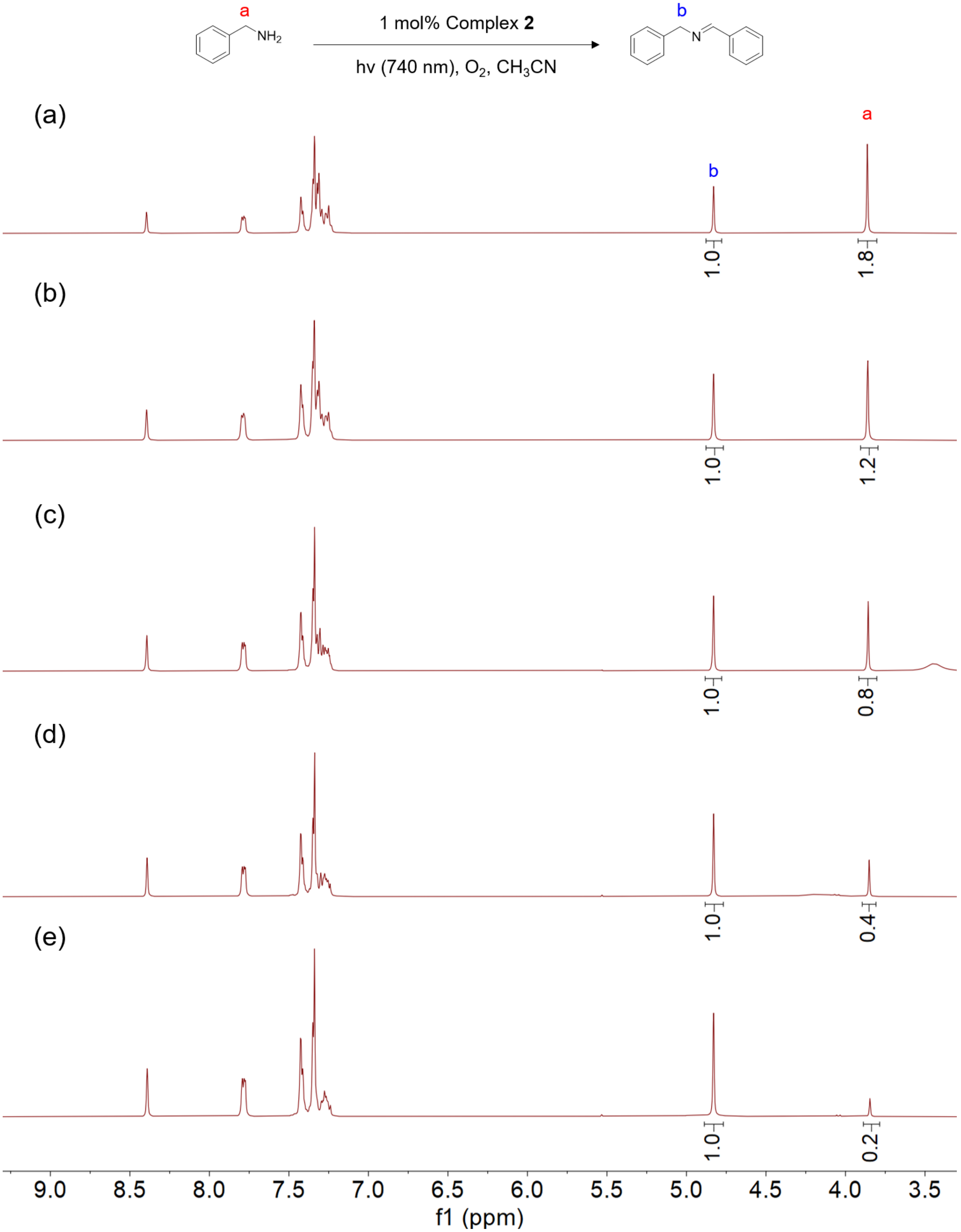

a
NH2
1 mol% Complex 2
hv (740 nm), O2, CH3CN
b
N
(a)
a
b
1.0
1.8
(b)
1.0
1.2
(c)
1.0
0.8
(d)
1.0
0.4
(e)
1.0
0.2
9.0
8.5
8.0
7.5
7.0
6.5
6.0
5.5
5.0
4.5
4.0
3.5
f1 (ppm)

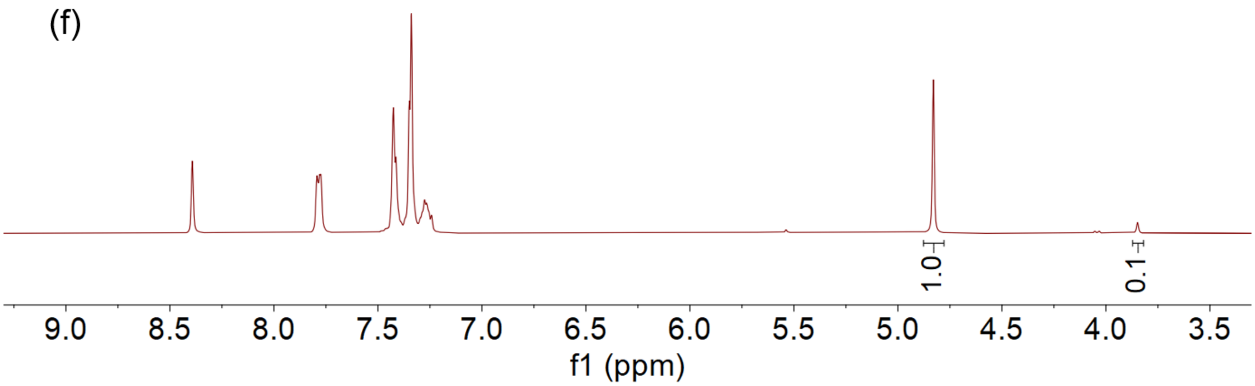

Supplementary Figure 27. ^{1}H NMR (400 MHz, $CDCl_3$, 298 K) spectra of benzyl amine C-N coupling using **2** as the photosensitizer with different irradiation time: (a) 20 min; (b) 30 min; (c) 40 min; (d) 60 min; (e) 80 min; (f) 100 min. Condition: 50 mM of benzyl amine, 1 mol% **2**, 5 mL CH_3CN, O_2, 740 nm LEDs, room temperature.

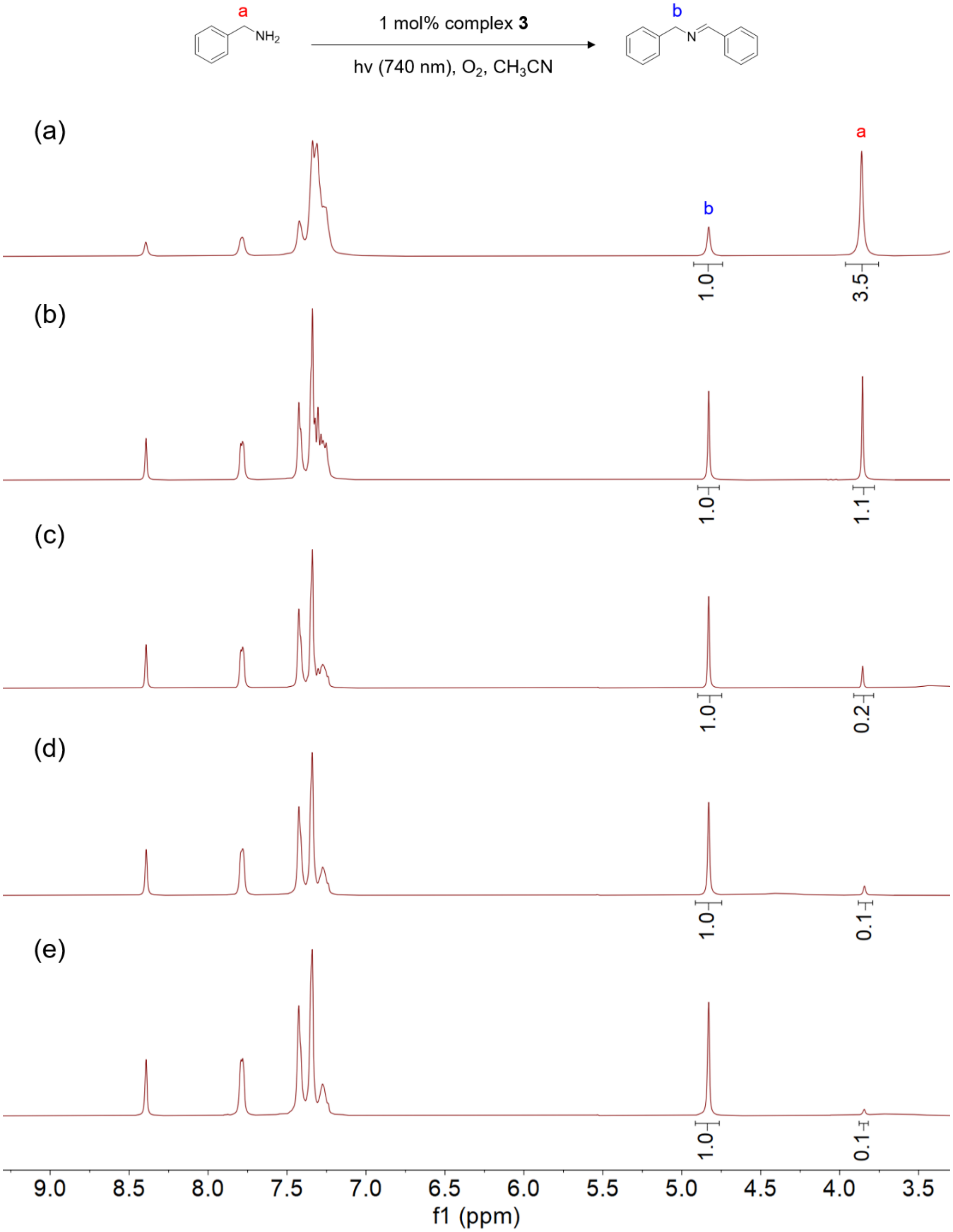

Supplementary Figure 28. ^{1}H NMR (400 MHz, $CDCl_3$, 298 K) spectra of benzyl amine C-N coupling using **3** as the photosensitizer with different irradiation time: (a) 10 min; (b) 20 min; (c) 30 min; (d) 40 min; (e) 50 min. Condition: 50 mM benzyl amine, 1 mol% **3**, 5 mL CH_3CN, O_2, 740 nm LEDs, room temperature.

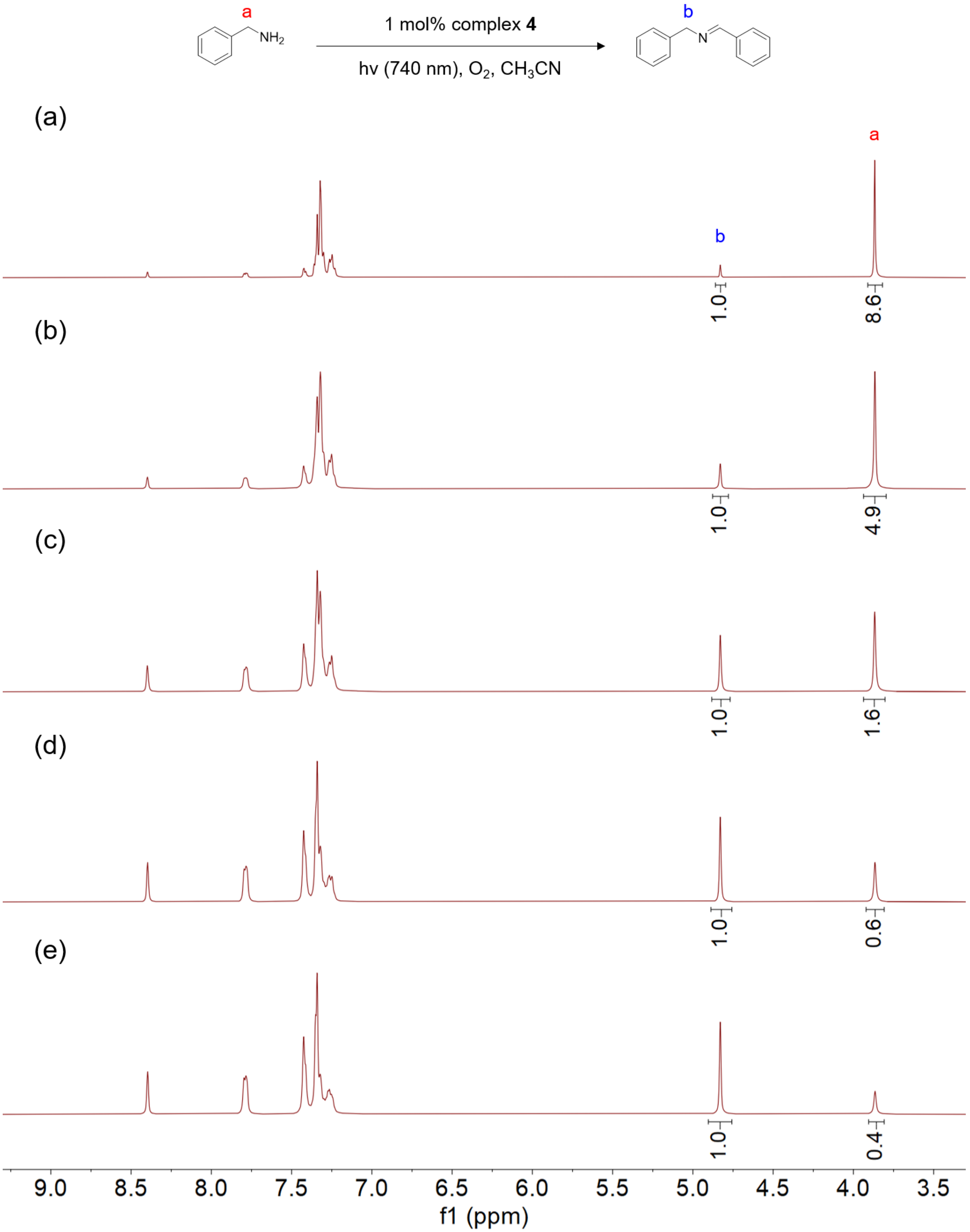

a
NH2
1 mol% complex 4
hv (740 nm), O2, CH3CN
b
N
(a)
a
b
1.0
8.6
(b)
1.0
4.9
(c)
1.0
1.6
(d)
1.0
0.6
(e)
1.0
0.4
9.0
8.5
8.0
7.5
7.0
6.5
6.0
5.5
5.0
4.5
4.0
3.5
f1 (ppm)

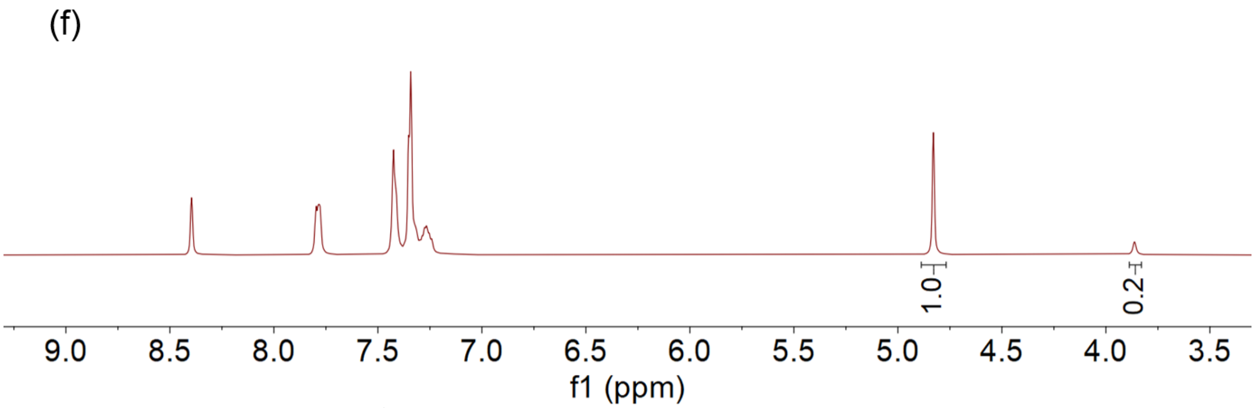

Supplementary Figure 29. ^{1}H NMR (400 MHz, $CDCl_3$, 298 K) spectra of benzyl amine C-N coupling using **4** as the photosensitizer with different irradiation time: (a) 10 min; (b) 20 min; (c) 40 min; (d) 80 min; (e) 120 min; (f) 160 min. Condition: 50 mM benzyl amine, 1 mol% **4**, 5 mL CH_3CN, O_2, 740 nm LEDs, room temperature.

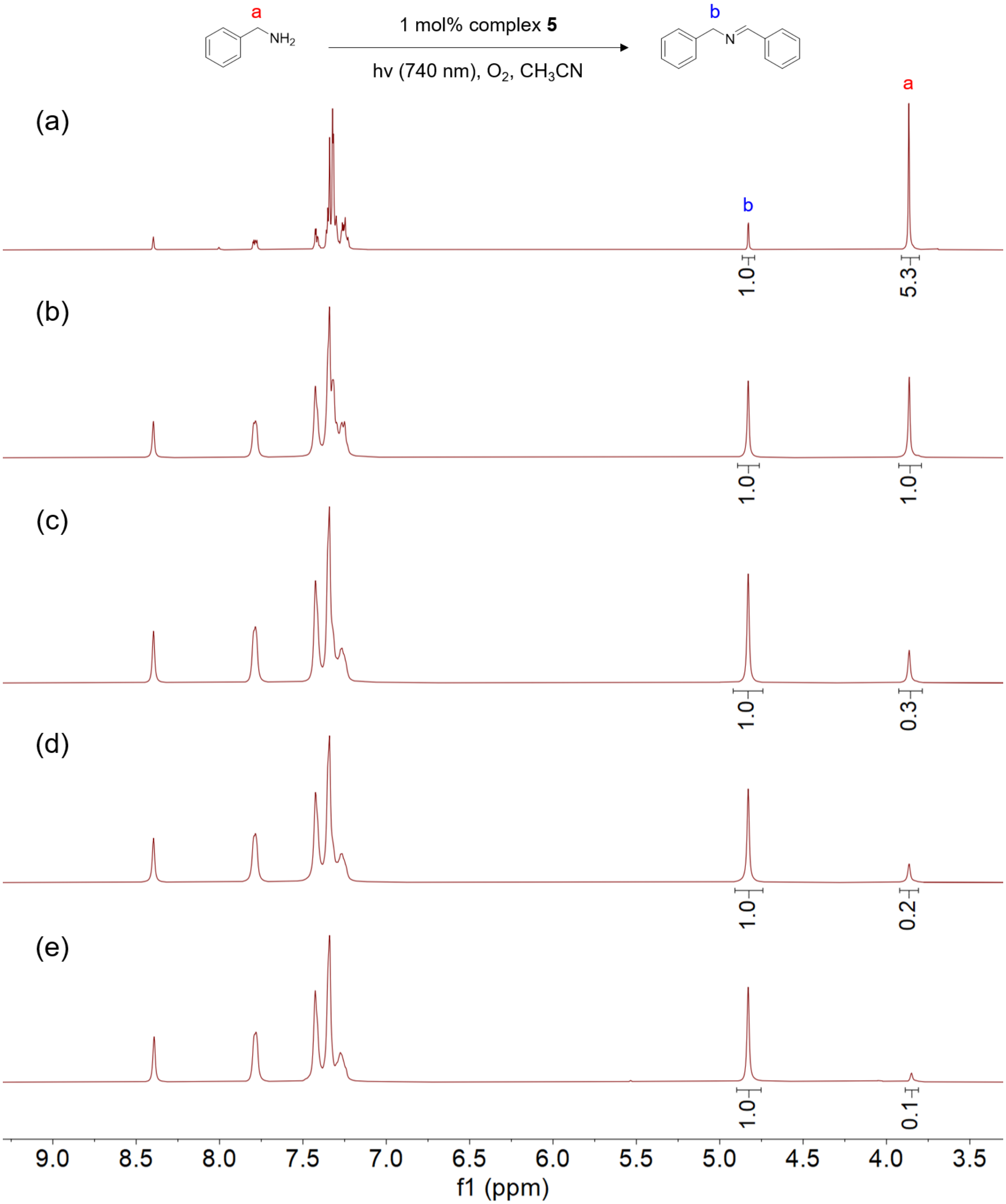

Supplementary Figure 30. ^{1}H NMR (400 MHz, $CDCl_3$, 298 K) spectra of benzyl amine C-N coupling using **5** as the photosensitizer with different irradiation time: (a) 3 min; (b) 10 min; (c) 15 min; (d) 20 min; (e) 30 min. Condition: 50 mM benzyl amine, 1 mol% **5**, 5 mL CH_3CN, O_2, 740 nm LEDs, room temperature.

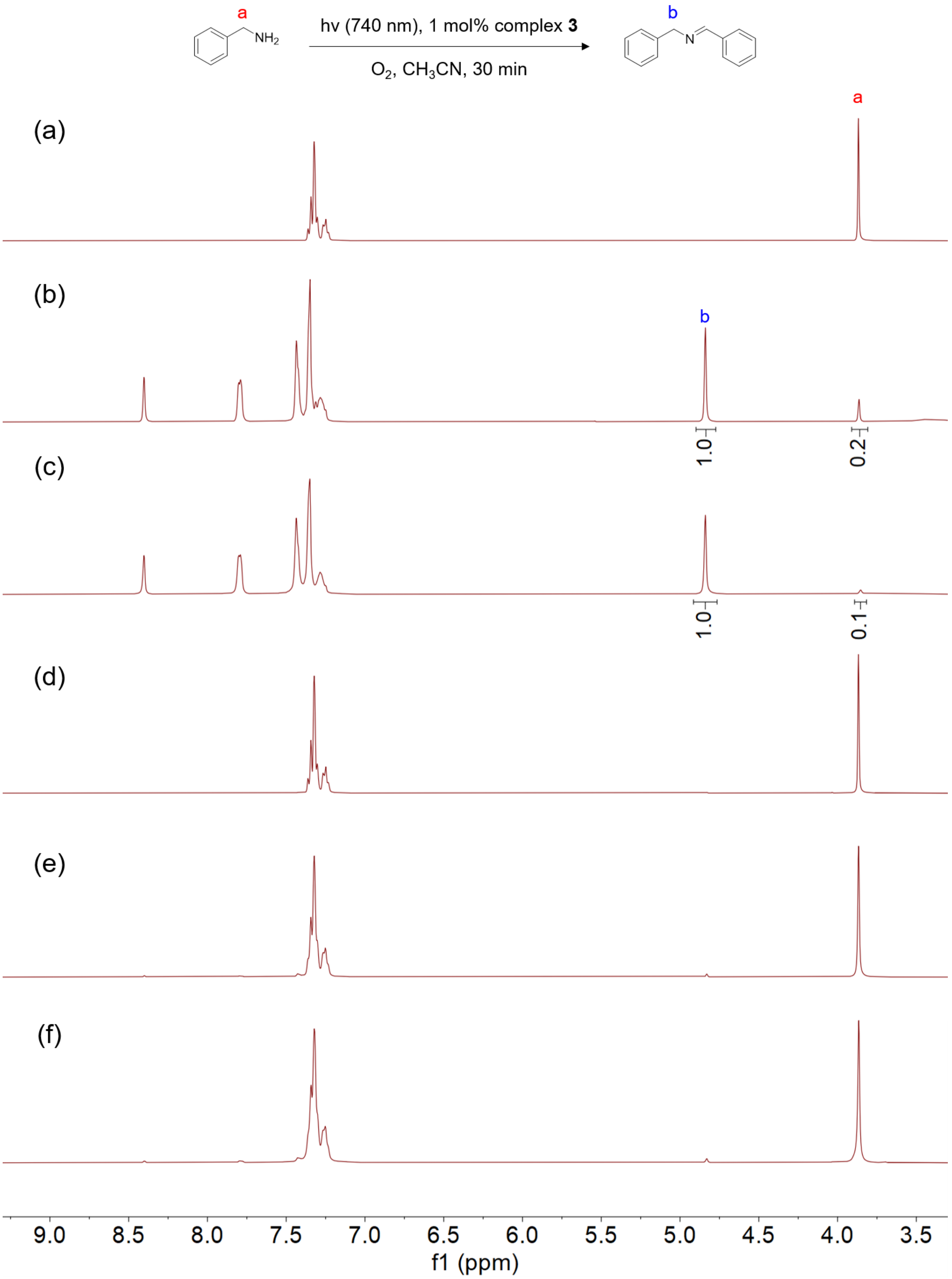
a
NH2
hv (740 nm), 1 mol% complex 3
O2, CH3CN, 30 min
b
N
a
(a)
(b)
b
1.0
0.2
(c)
1.0
0.1
(d)
(e)
(f)
9.0
8.5
8.0
7.5
7.0
6.5
6.0
5.5
5.0
4.5
4.0
3.5
f1 (ppm)

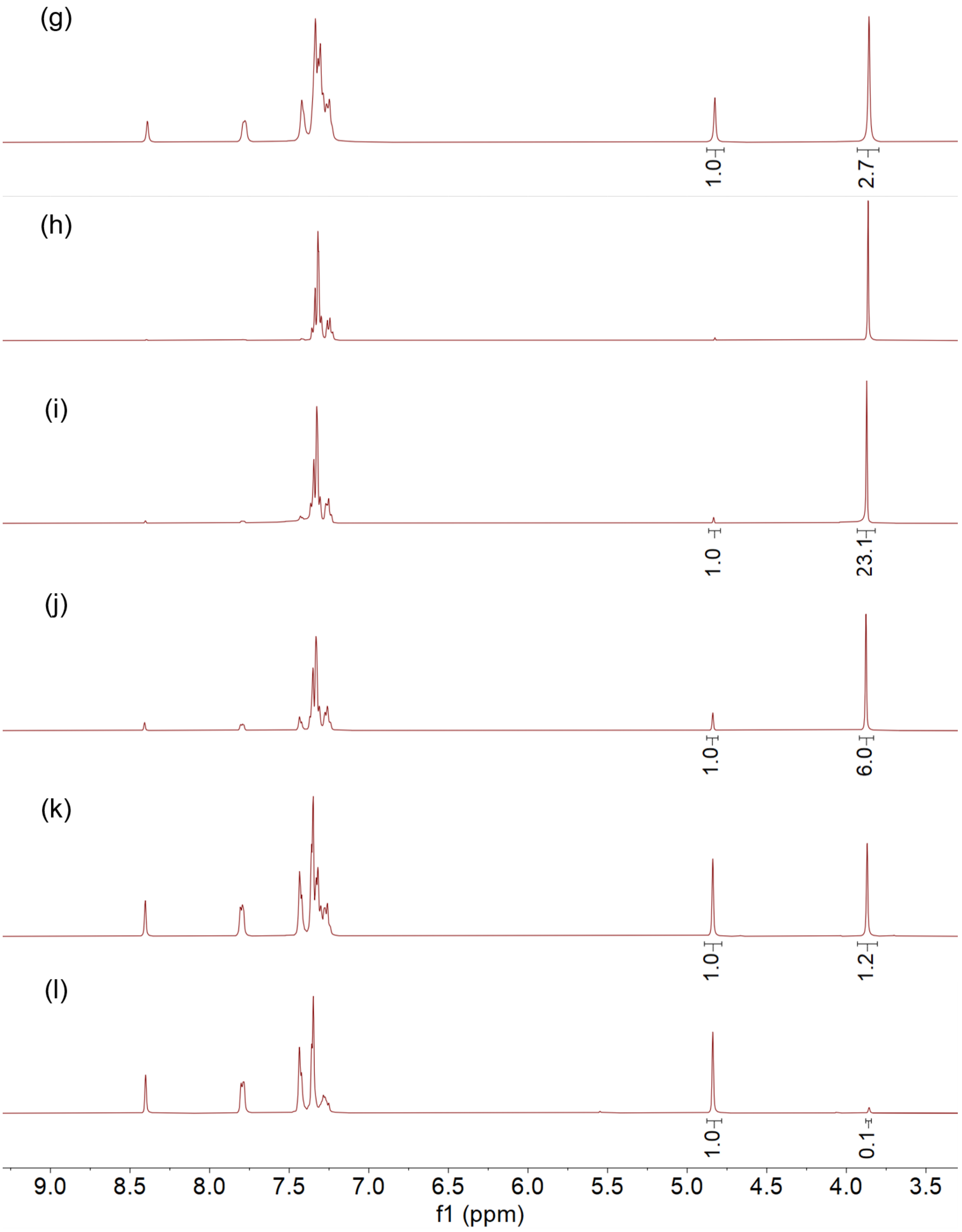
(g)
1.0
2.7
(h)
(i)
1.0
23.1
(j)
1.0
6.0
(k)
1.0
1.2
(l)
1.0
0.1
9.0 8.5 8.0 7.5 7.0 6.5 6.0 5.5 5.0 4.5 4.0 3.5
f1 (ppm)

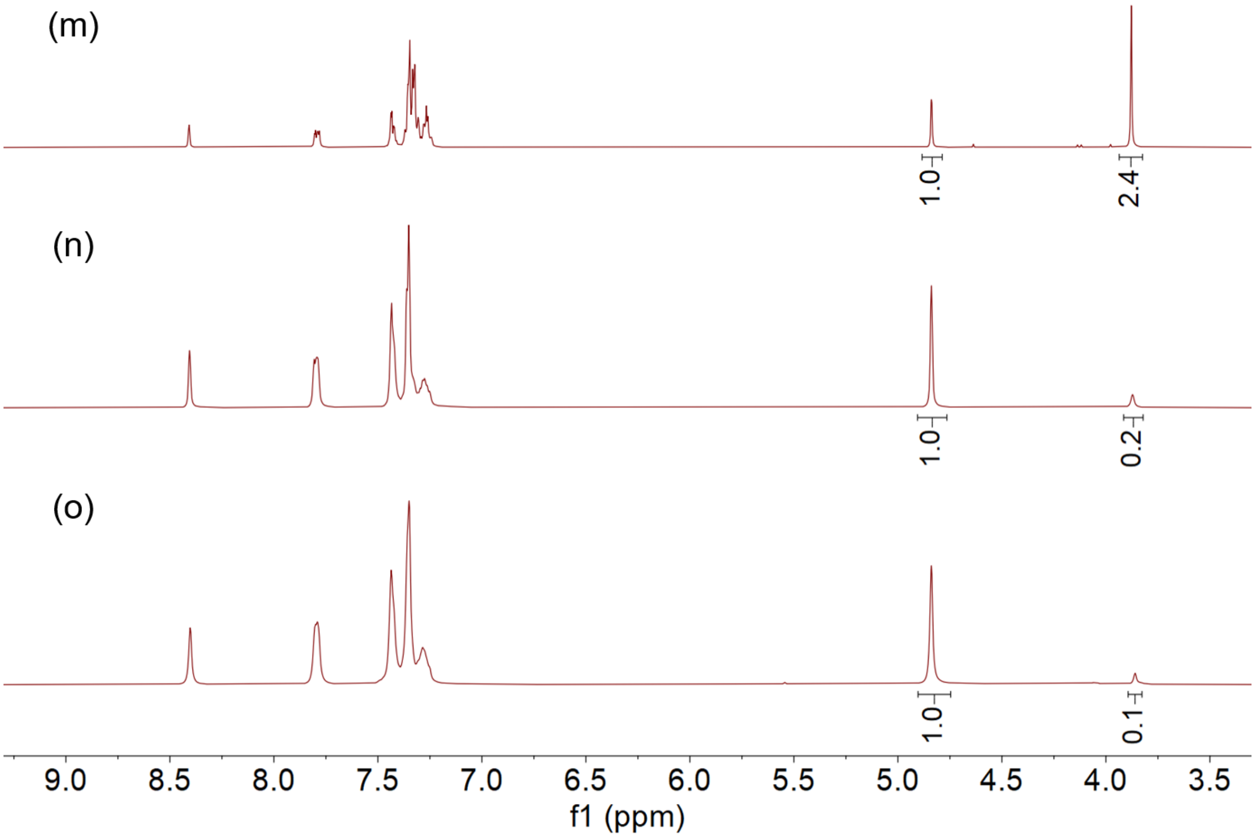

Supplementary Figure 31. ^{1}H NMR (400 MHz, $CDCl_3$, 298 K) spectra of benzyl amine C-N coupling. (a) Standard benzyl amine; (b) Standard condition: 50 mM benzyl amine, 1 mol%, 5 mL CH_3CN, O_2, 740 nm LEDs, room temperature, 30 min; (c) time is 50 min; (d) no photosensitizer; (e) no irradiation; (f) Ar atmosphere; (g) Air atmosphere; (h) photosensitizer is $[Ru(bpy)_3](PF_6)_2$; (i) photosensitizer is **1**; (j) photosensitizer is complex **1** and time is 4 h; (k) photosensitizer is **2**; (l) photosensitizer is **2** and time is 100 min; (m) photosensitizer is **4**; (n) photosensitizer **4** and time is 160 min; (o) photosensitizer is **5**.

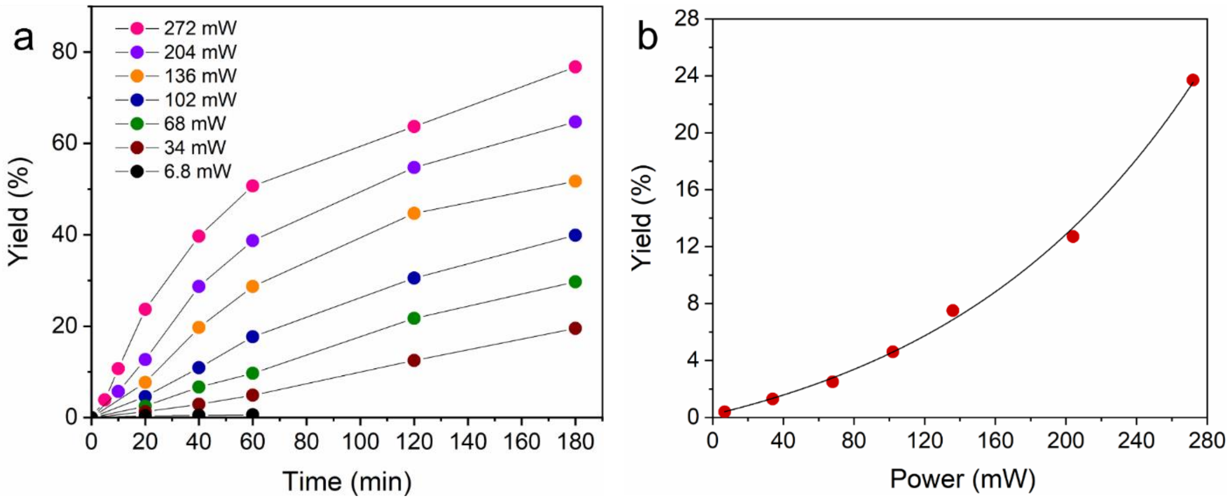

Supplementary Figure 32. (a) Yield change over time upon irradiation at 730 nm of different power. (b) The obtained yields after 20 min irradiation at 730 nm of different power. Conditions: 50 mM benzyl amine, 1 mol% **5**, 2 mL CH_3CN, O_2, irradiation using 730 nm LED (M730L5) of controlled power at room temperature with a 695 nm long-pass filter.

The LED illumination power of our newly purchased 730 nm LED can be finely tuned by the LED driver (Thorlabs, DC2200). As shown in **Supplementary Figure 32a**, during the first 20 min photocatalysis, the product yield is linearly increased with the irradiation duration as the starting material concentration is not depleted to become a limiting factor. Consequently, we plotted the yield obtained at 20 min versus the LED power in **Supplementary Figure 32b**. It is apparent that when we increased the LED power from 6.8 to 272 mW, a non-linear increase in yield was observed, in agreement with the two-photon absorption mechanism of our photocatalyst (complex **5** in this case).

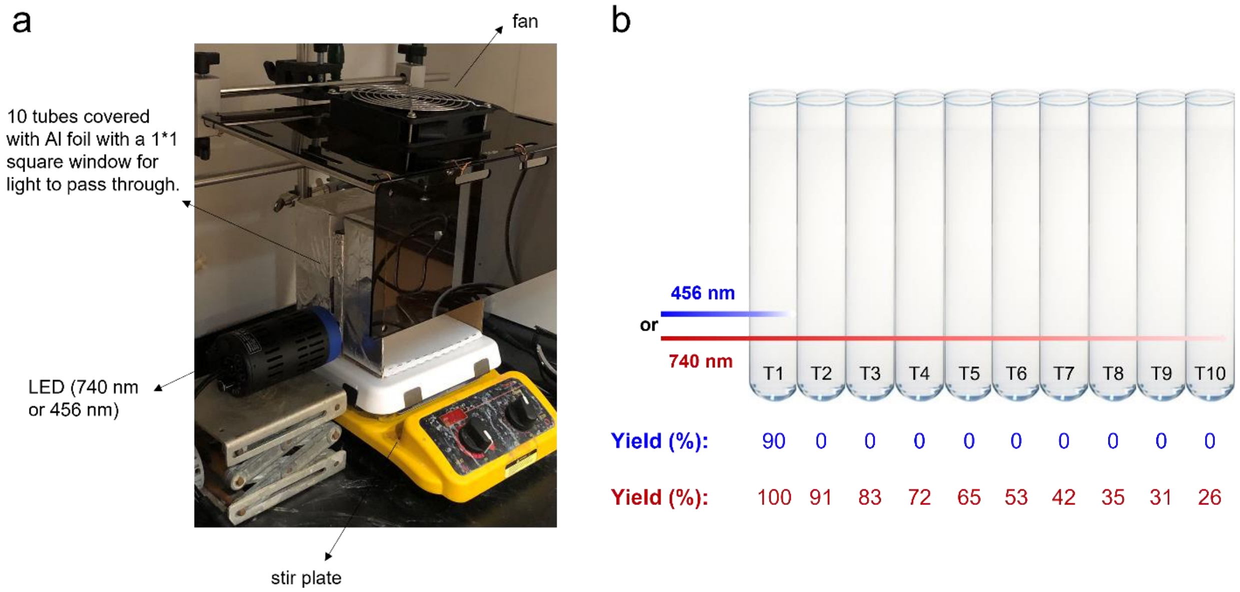

Supplementary Figure 33. (a) Experimental set-up. (b) Yields of the photocatalytic C-N coupling of benzyl amine in different reaction tubes. Photocatalysis condition of each tube: 50 mM benzyl amine, 1 mol% **5**, 4 mL CH_3CN, open to air, irradiation at 456 or 740 nm for 6 h at room temperature.

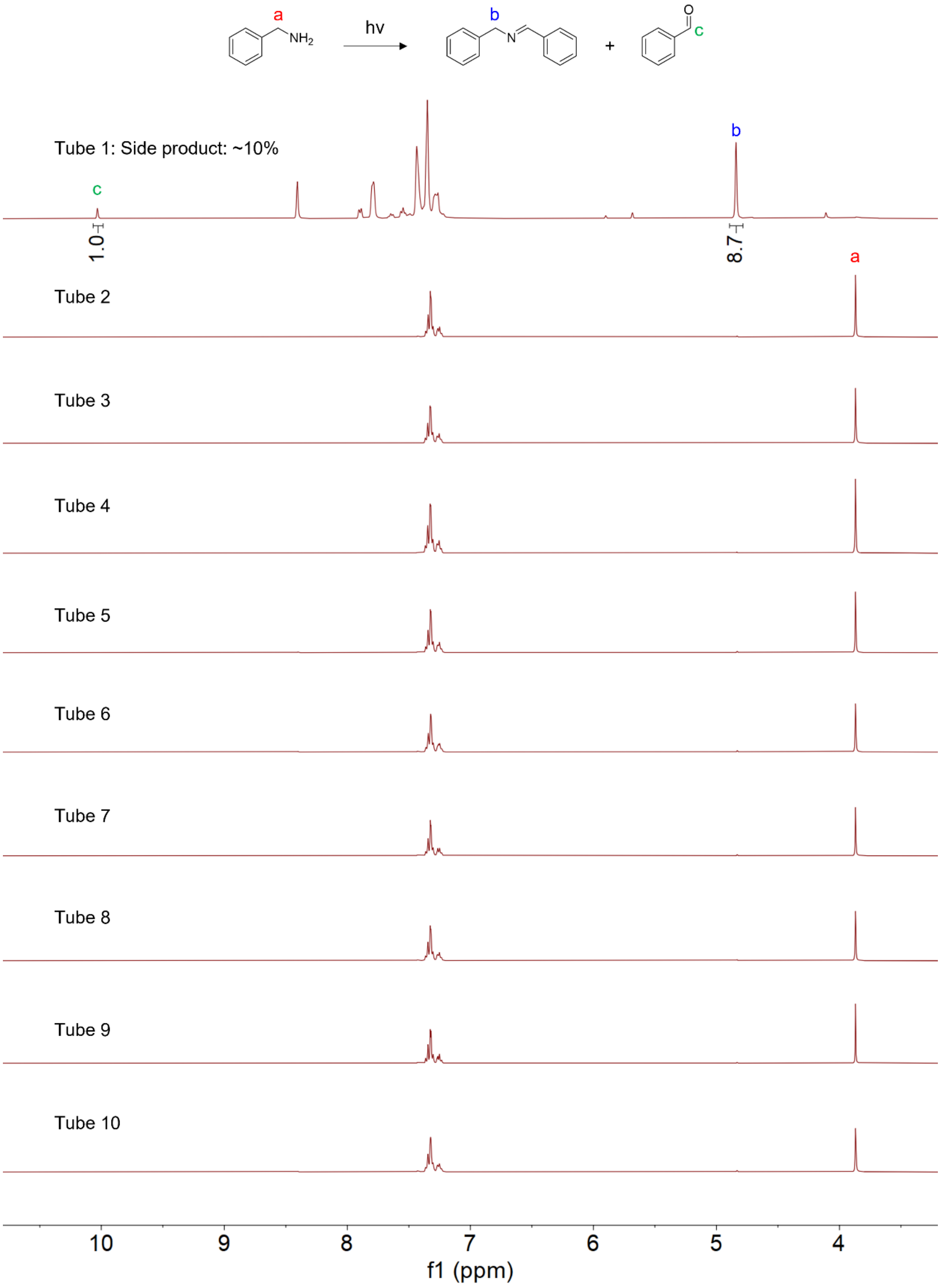

Supplementary Figure 34. ^{1}H NMR (400 MHz, $CDCl_3$, 298 K) spectra of the photocatalytic C-N coupling of benzyl amine in different reaction tubes. Photocatalysis condition of each tube: 50 mM benzyl amine, 1 mol% **5**, 4 mL CH_3CN, open to air, irradiation at 456 nm for 6 h at room temperature.

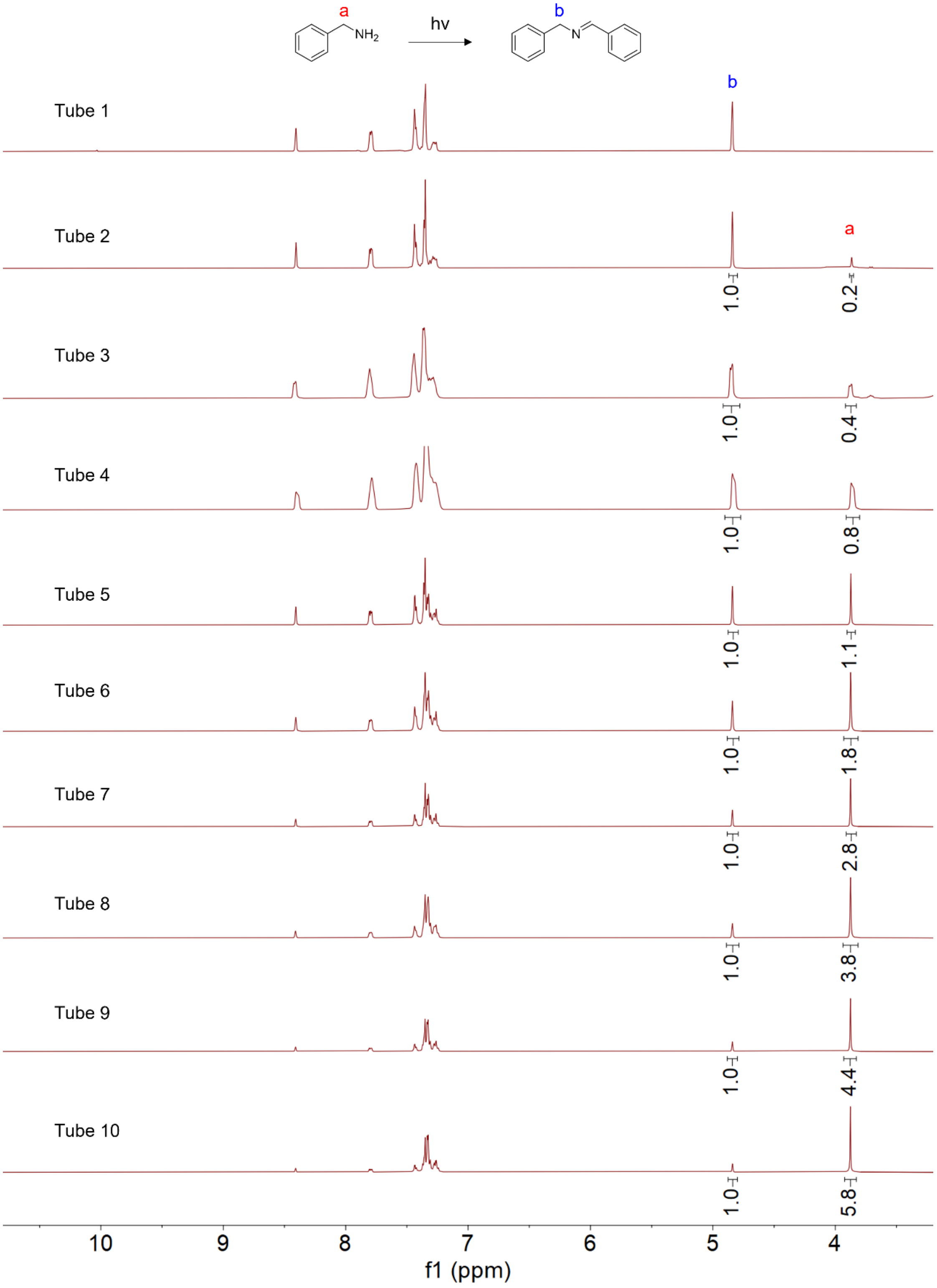

Supplementary Figure 35. ^{1}H NMR (400 MHz, $CDCl_3$, 298 K) spectra of the photocatalytic C-N coupling of benzyl amine in different reaction tubes. Photocatalysis condition of each tube: 50 mM benzyl amine, 1 mol% **5**, 4 mL CH_3CN, open to air, irradiation at 740 nm for 6 h at room temperature.

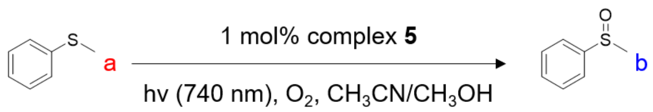
1 mol% complex 5
hv (740 nm), O2, CH3CN/CH3OH
S
a
O
S
b

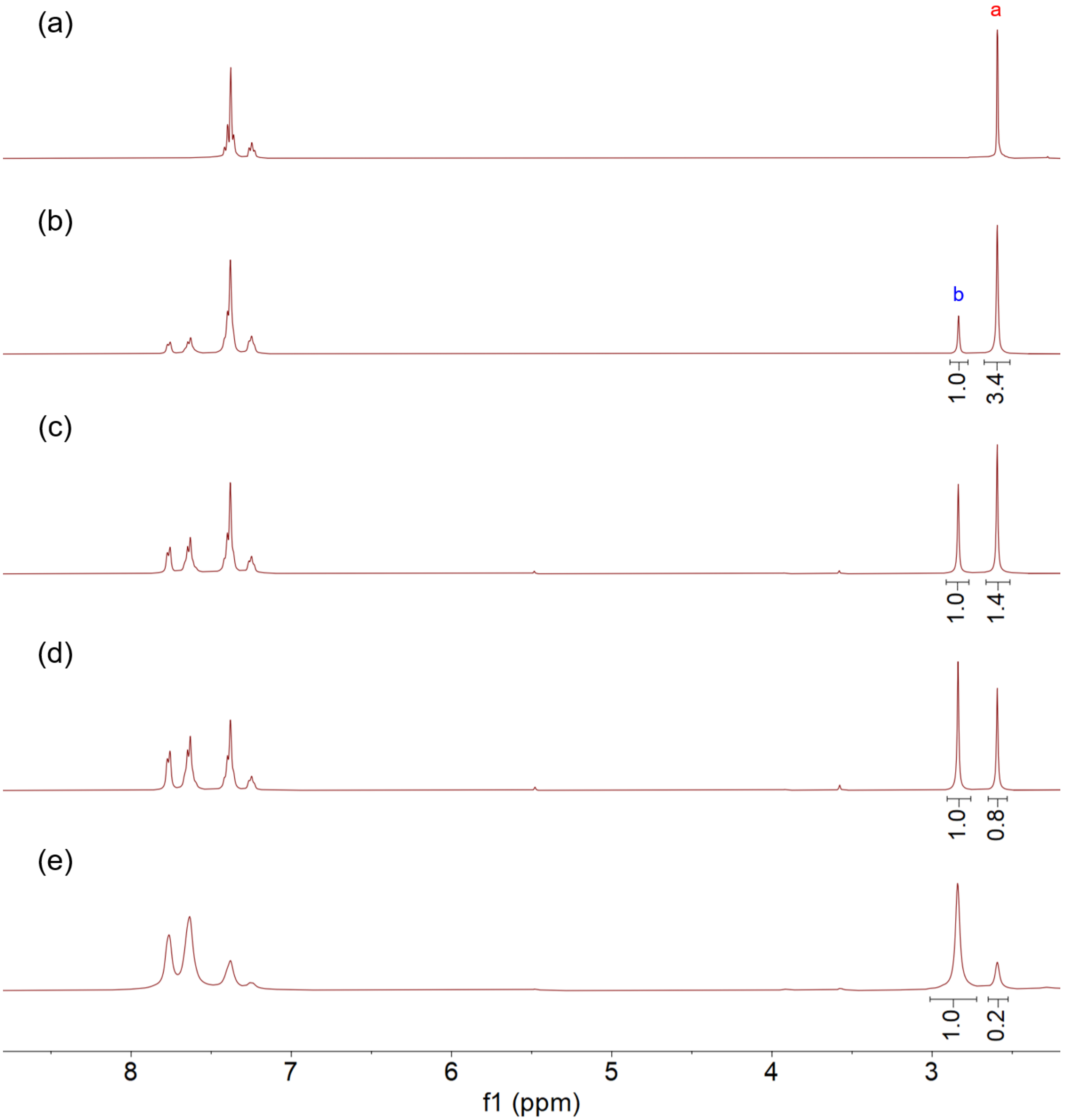
(a)
a
(b)
b
1.0
3.4
(c)
1.0
1.4
(d)
1.0
0.8
(e)
1.0
0.2
8
7
6
5
4
3
f1 (ppm)

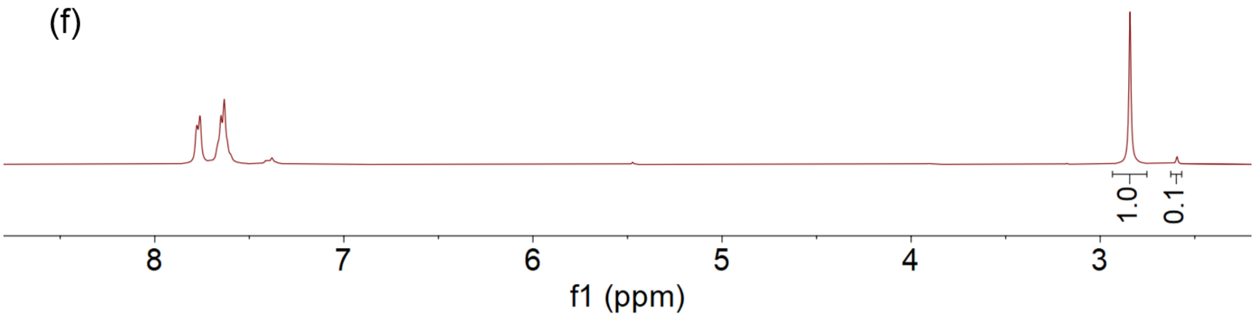

Supplementary Figure 36. ^{1}H NMR (400 MHz, $CDCl_3$, 298 K) spectra of thioanisole sulfoxidation using **5** as the photosensitizer with different irradiation time: (a) standard thioanisole; (b) 20 min; (c) 40 min; (d) 1 h; (e) 2 h; (f) 3 h. Condition: 50 mM benzyl amine, 1 mol% **5**, 4 mL CH_3CN, 1 mL CH_3OH, O_2, 740 nm LEDs, room temperature.

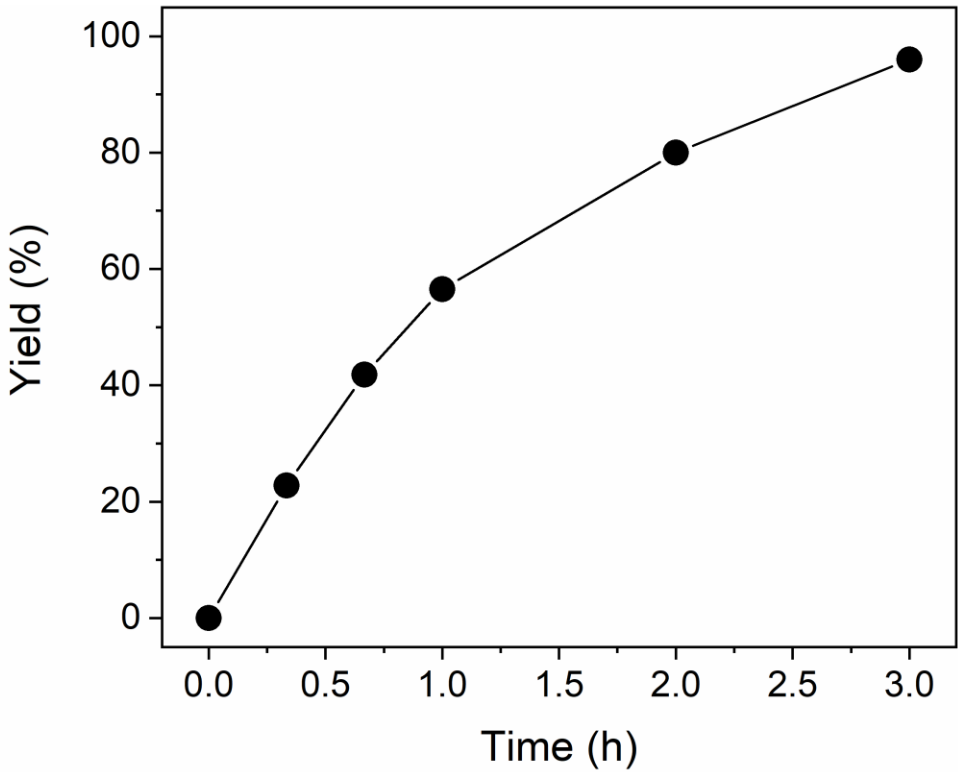

Supplementary Figure 37. Yield change over time of thioanisole sulfoxidation. Condition: 50 mM thioanisole, 1 mol% **5**, 4 mL CH_3CN, 1 mL CH_3OH, O_2, 740 nm LEDs, room temperature.

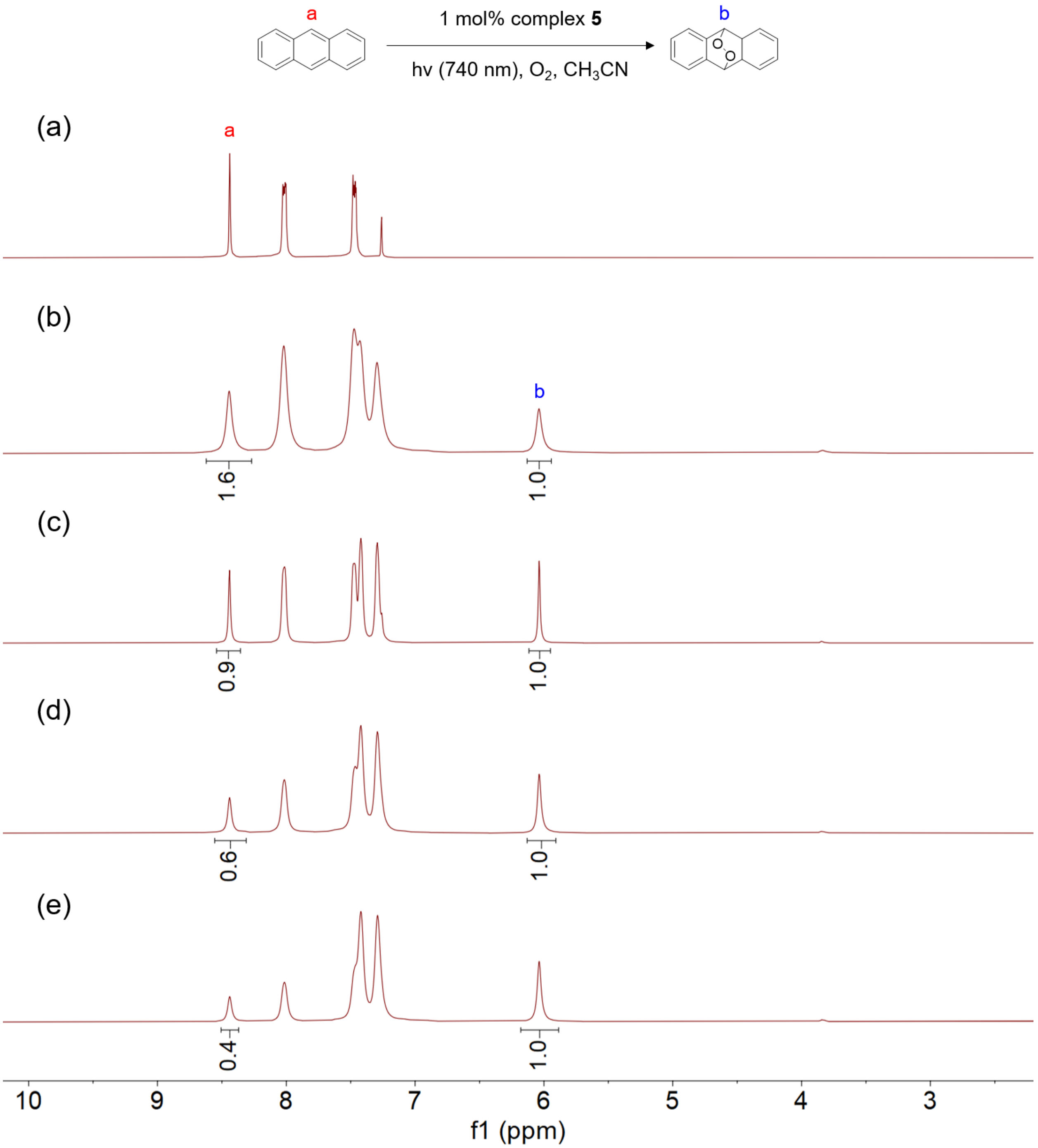
a
1 mol% complex **5**
hv (740 nm), O_2, CH_3CN
b
O
O
(a)
a
(b)
b
1.6
1.0
(c)
0.9
1.0
(d)
0.6
1.0
(e)
0.4
1.0
10
9
8
7
6
5
4
3
f1 (ppm)

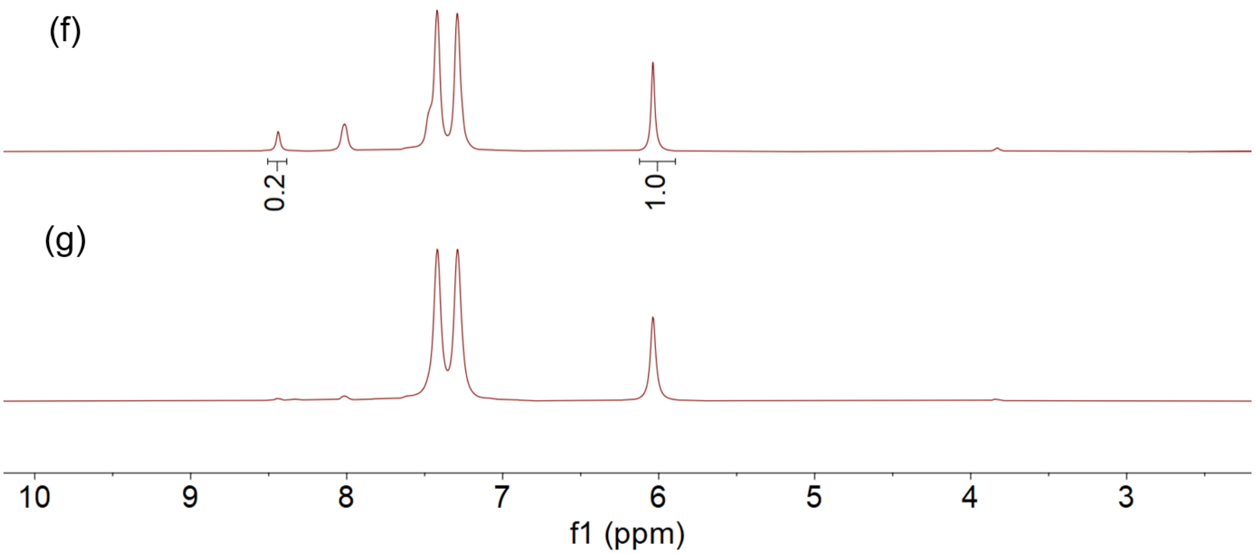

Supplementary Figure 38. ^{1}H NMR (400 MHz, $CDCl_3$, 298 K) spectra of anthracene [4+2] Diels-Alder reaction using **5** as the photosensitizer with different irradiation time: (a) standard anthracene; (b) 20 min; (c) 40 min; (d) 1 h; (e) 2 h; (f) 4 h; (g) 6 h. Condition: 10 mM anthracene, 1 mol% **5**, 5 mL CH_3CN, O_2, 740 nm LEDs, room temperature.

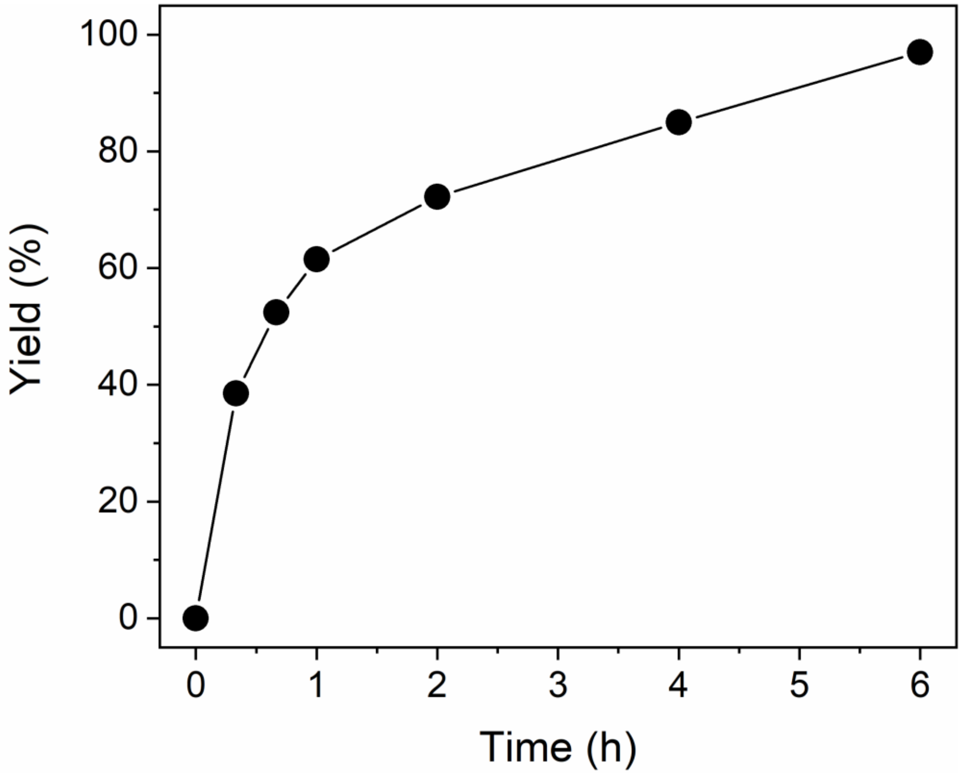

Supplementary Figure 39. Yield change over time of anthracene [4+2] Diels-Alder reaction. Condition: 10 mM anthracene, 1 mol% **5**, 4 mL CH_3CN, O_2, 740 nm LEDs, room temperature.

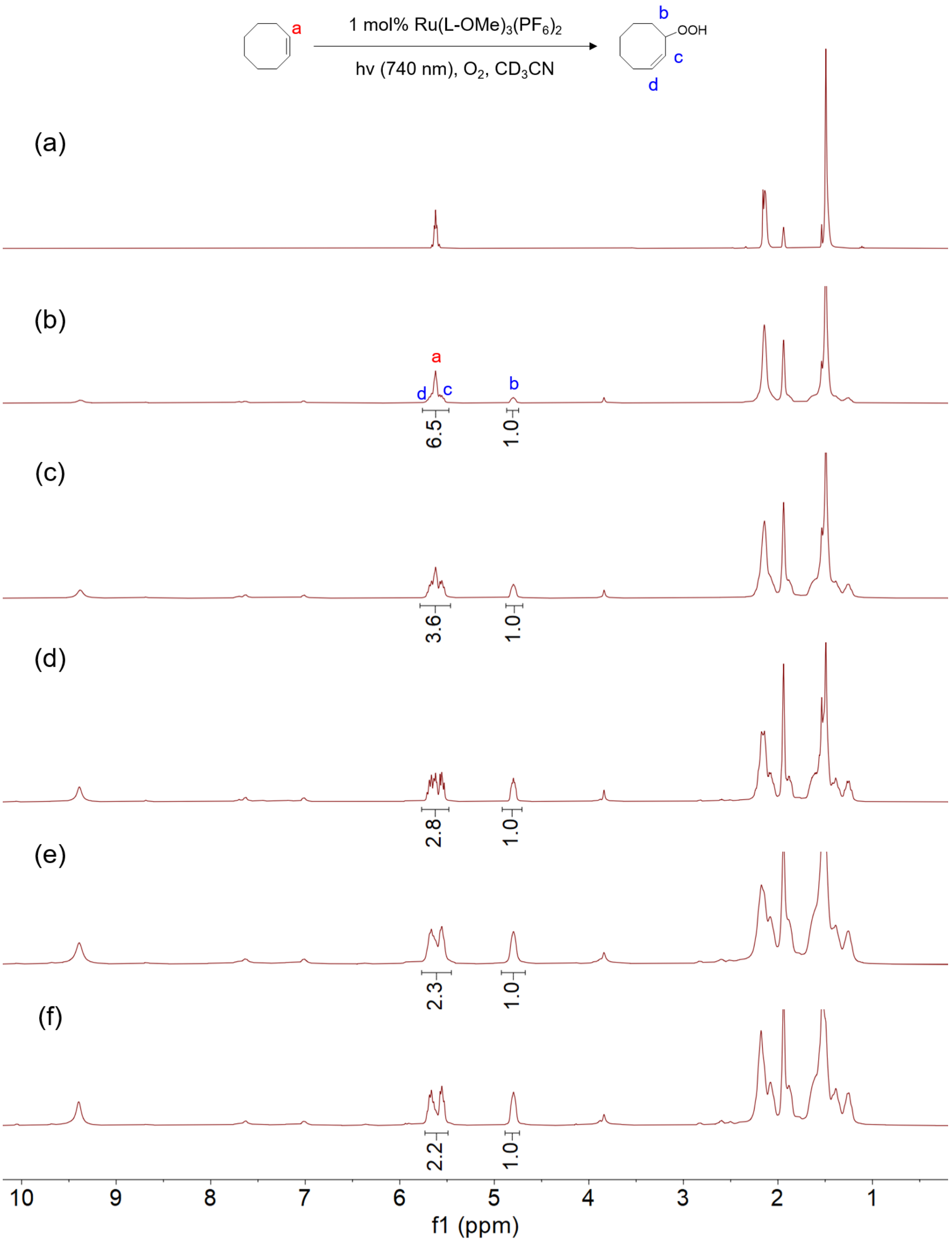

Supplementary Figure 40. ^{1}H NMR (400 MHz, $CDCl_3$, 298 K) spectra of cyclooctene oxidation using **5** as the photosensitizer with different irradiation time: (a) standard cyclooctene; (b) 1 h; (c)

3 h; (d) 5 h; (e) 7 h; (f) 10 h. Condition: 50 mM cyclooctene, 1 mol% **5**, 5 mL CD_3CN, O_2, 740 nm LEDs, room temperature.

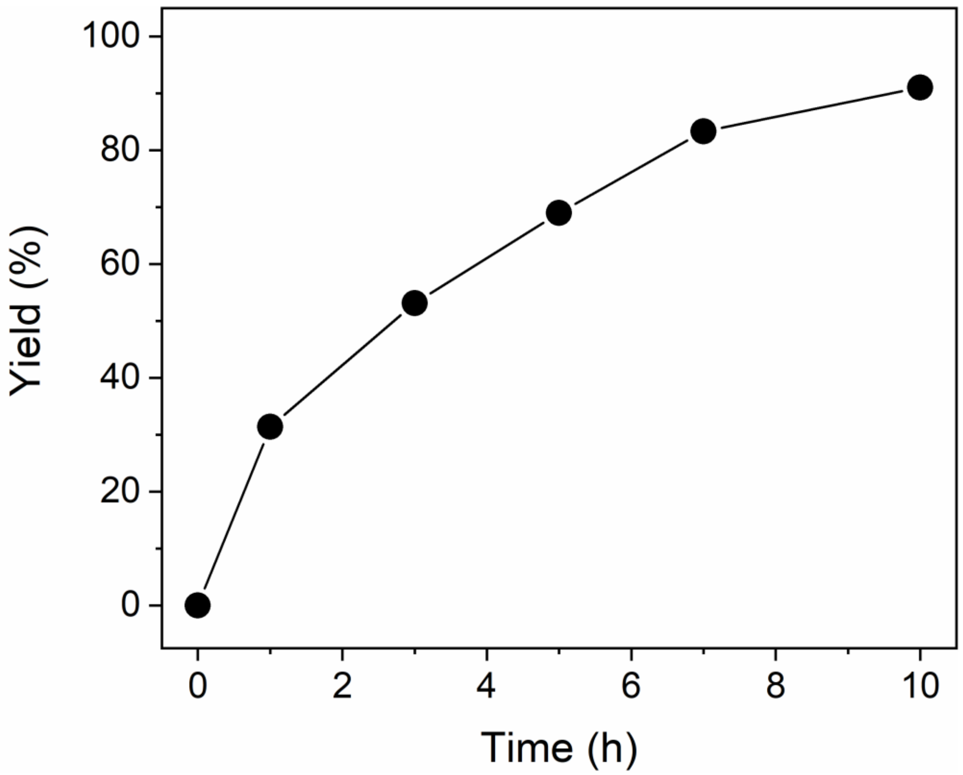

Supplementary Figure 41. Yield change over time of anthracene oxidation. Condition: 10 mM anthracene, 1 mol% **5**, 5 mL CH_3CN, O_2, 740 nm LEDs, room temperature.

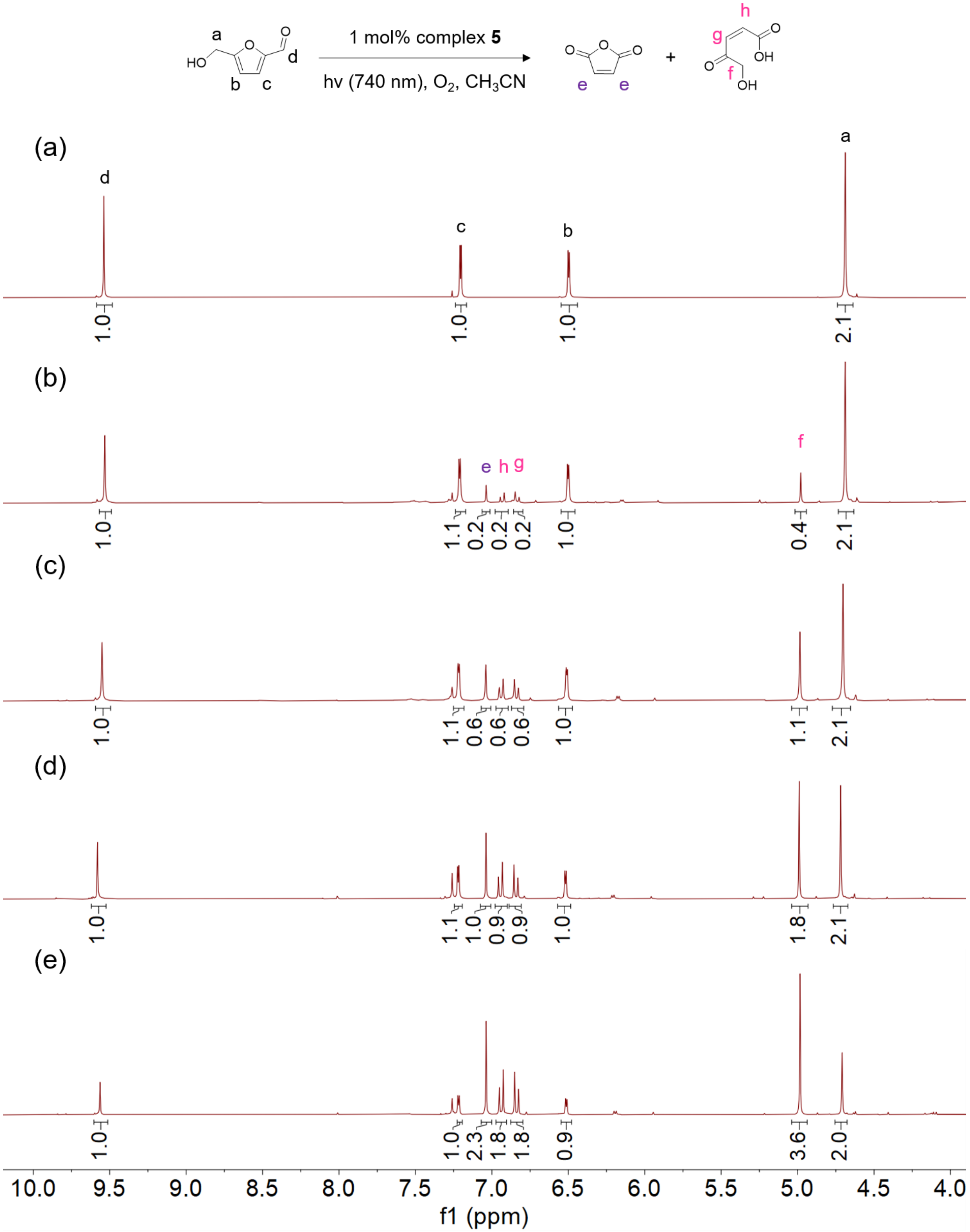
1 mol% complex 5
hv (740 nm), O2, CH3CN
(a)
(b)
(c)
(d)
(e)
f1 (ppm)

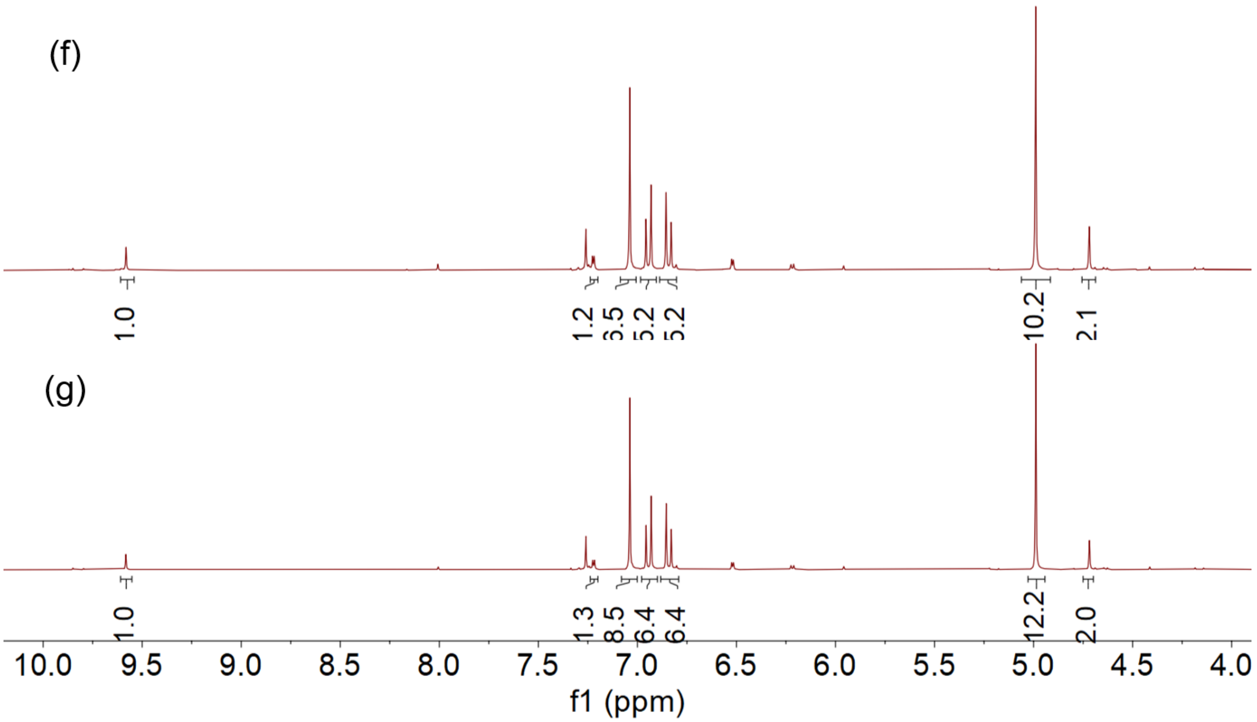

Supplementary Figure 42. ^{1}H NMR (400 MHz, $CDCl_3$, 298 K) spectra of HMF oxidation using **5** as the photosensitizer with different irradiation time: (a) standard HMF; (b) 30 min; (c) 1 h; (d) 2 h; (e) 3 h; (f) 4 h; (g) 5 h. Condition: 50 mM HMF, 1 mol% **5**, 5 mL CH_3CN, O_2, 740 nm LEDs, room temperature.

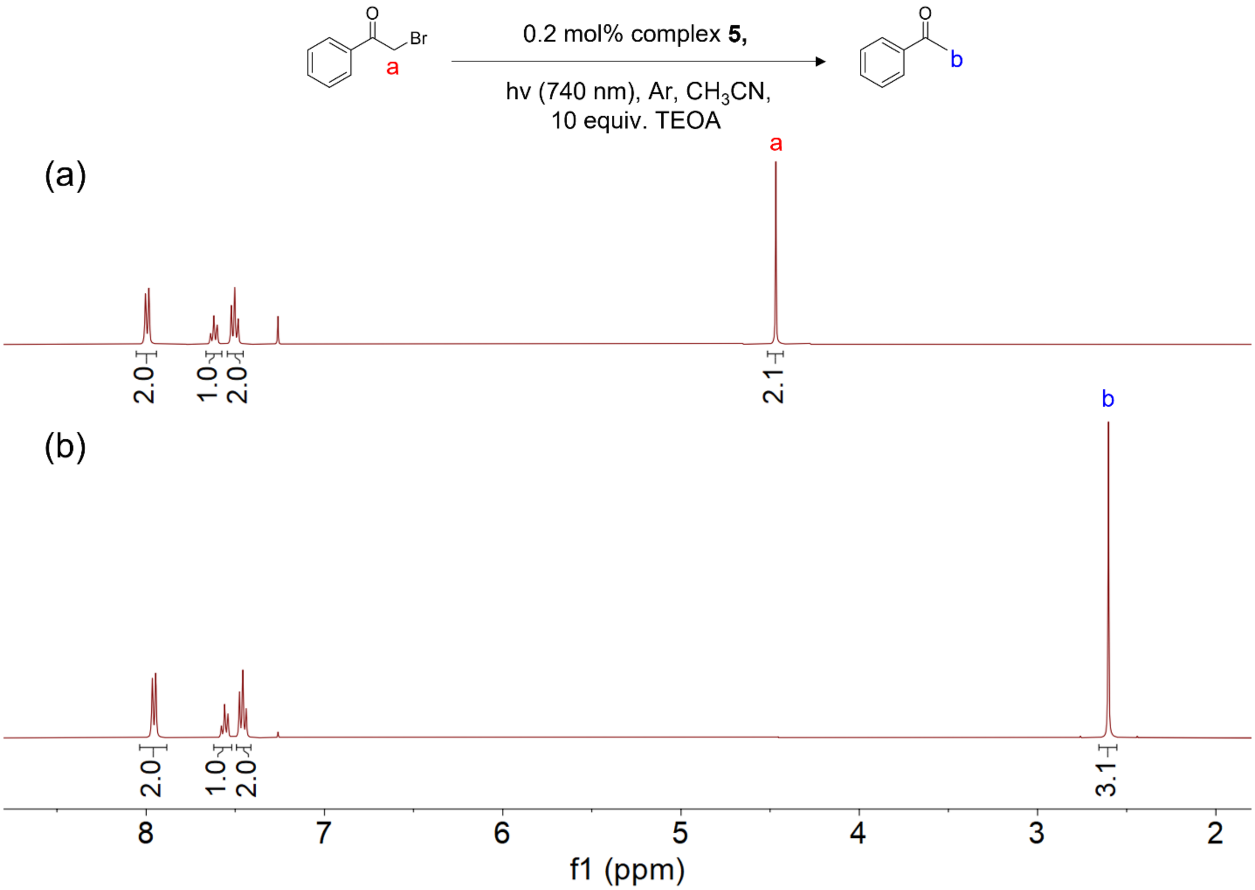

Supplementary Figure 43. ^{1}H NMR (400 MHz, $CDCl_3$, 298 K) spectra before (a) and after (b) dehalogenation of phenacyl bromide using **5** as the photosensitizer. Condition: 50 mM phenacyl bromide, 10 equiv. TEOA, 0.2 mol% **5**, 5 mL CH_3CN, Ar, 740 nm LEDs, room temperature, 8 h, purified via column chromatography (hexane/ethyl acetate = 100/3).

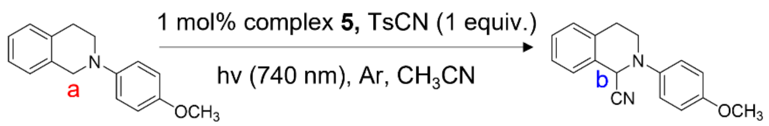

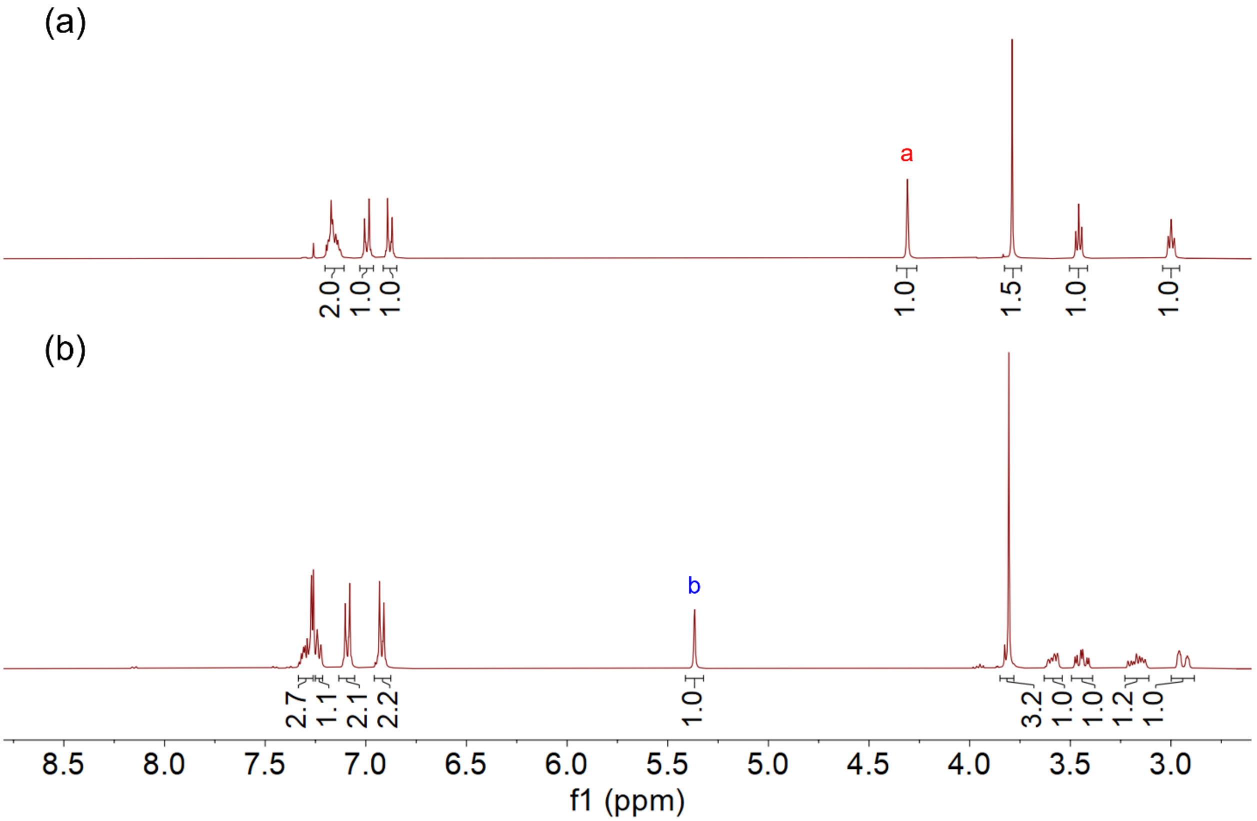

Supplementary Figure 44. ^{1}H NMR (400 MHz, $CDCl_3$, 298 K) spectra before (a) and after (b) cyanation of tetrahydroisoquinoline using **5** as the photosensitizer. Condition: 50 mM tetrahydroisoquinoline, 1 mol% **5**, 5 mL CH_3CN, Ar, 740 nm LEDs, room temperature, 24 h, purified via column chromatography (hexane/ethyl acetate = 100/3).

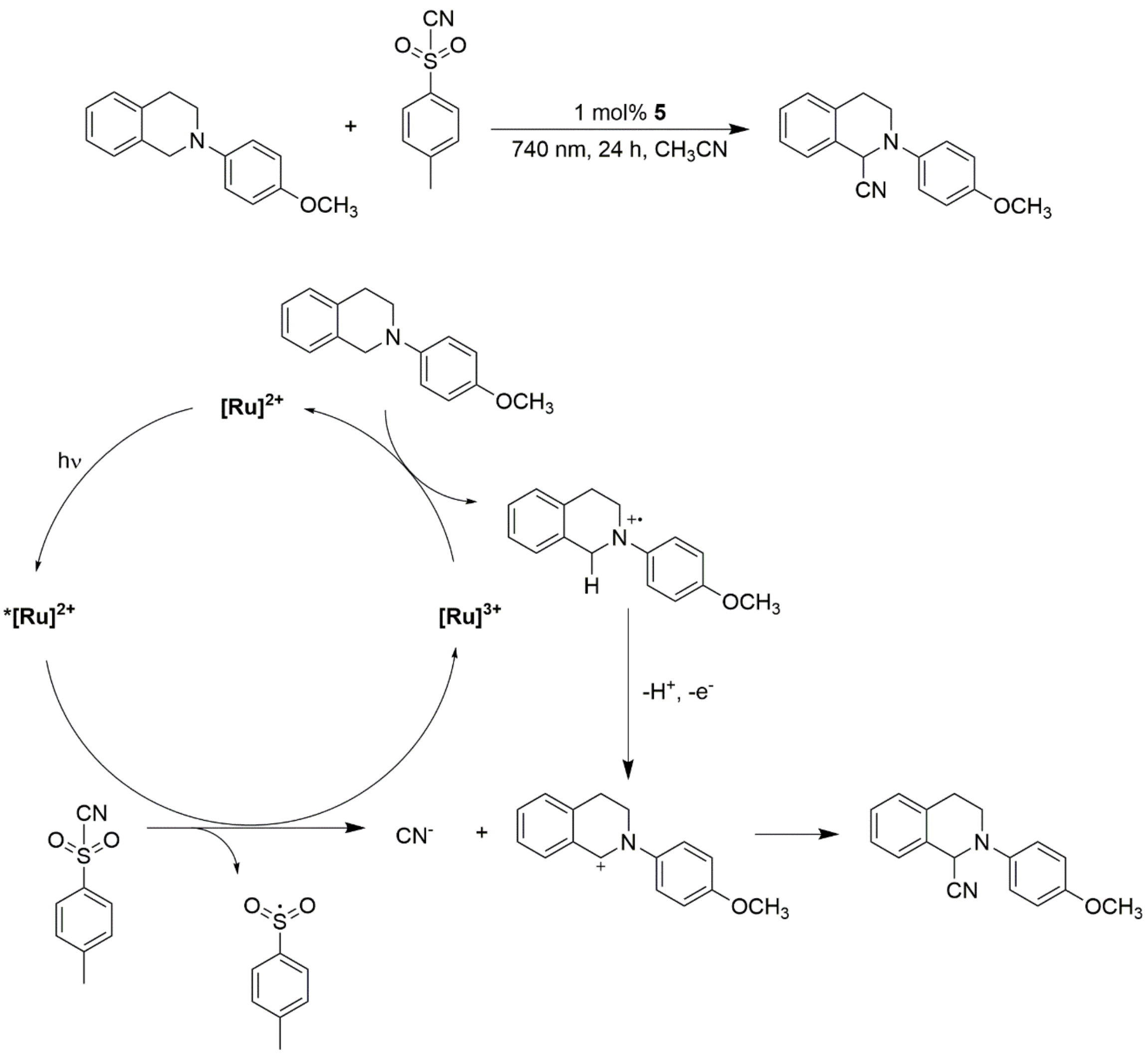

Supplementary Figure 45. Proposed photocatalytic cycle of cyanation using **5** as the photosensitizer under 740 nm irradiation. **[Ru]$^{2+}$** represents **5**.

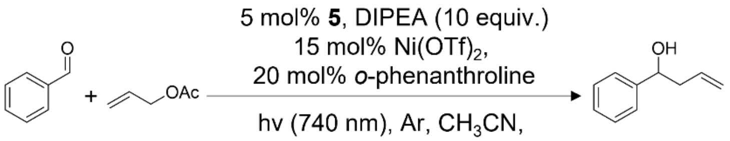

(a)

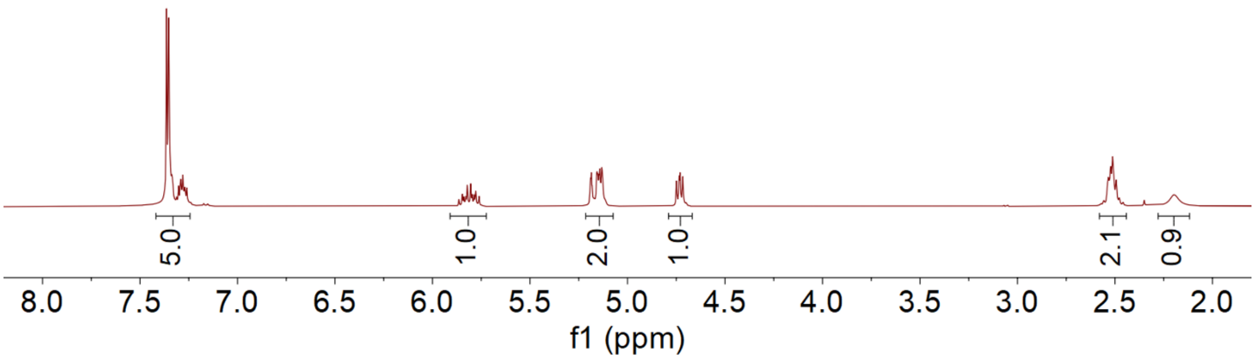

(b)

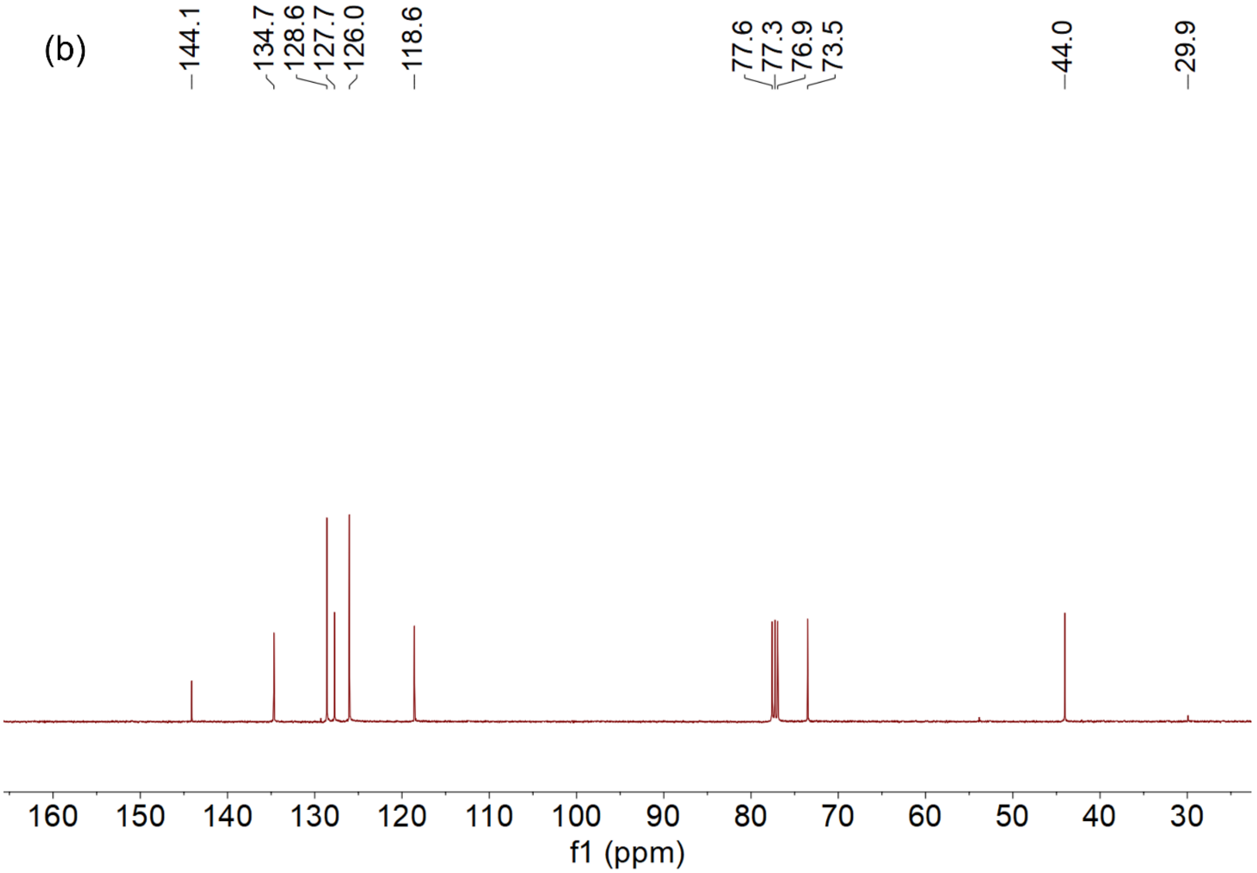

Supplementary Figure 46. ^{1}H NMR (400 MHz, $CDCl_3$, 298 K) (a) and $^{13}C\{^{1}H\}$ NMR (101 MHz, $CDCl_3$, 298 K) (b) spectra after C-C coupling between benzaldehyde and allyl acetate using **5** as the photosensitizer and $Ni(OTf)_2$ as a cocatalyst. Condition: 50 mM benzaldehyde, 10 equiv. allyl acetate, 5 mol% **5**, 15 mol% $Ni(OTf)_2$, 20 mol% *o*-phenanthroline, 10 equiv. DIPEA, 2 mL CH_3CN, Ar, 740 nm LEDs, room temperature, 72 h, purified via column chromatography (hexane/ethyl acetate = 10/1).

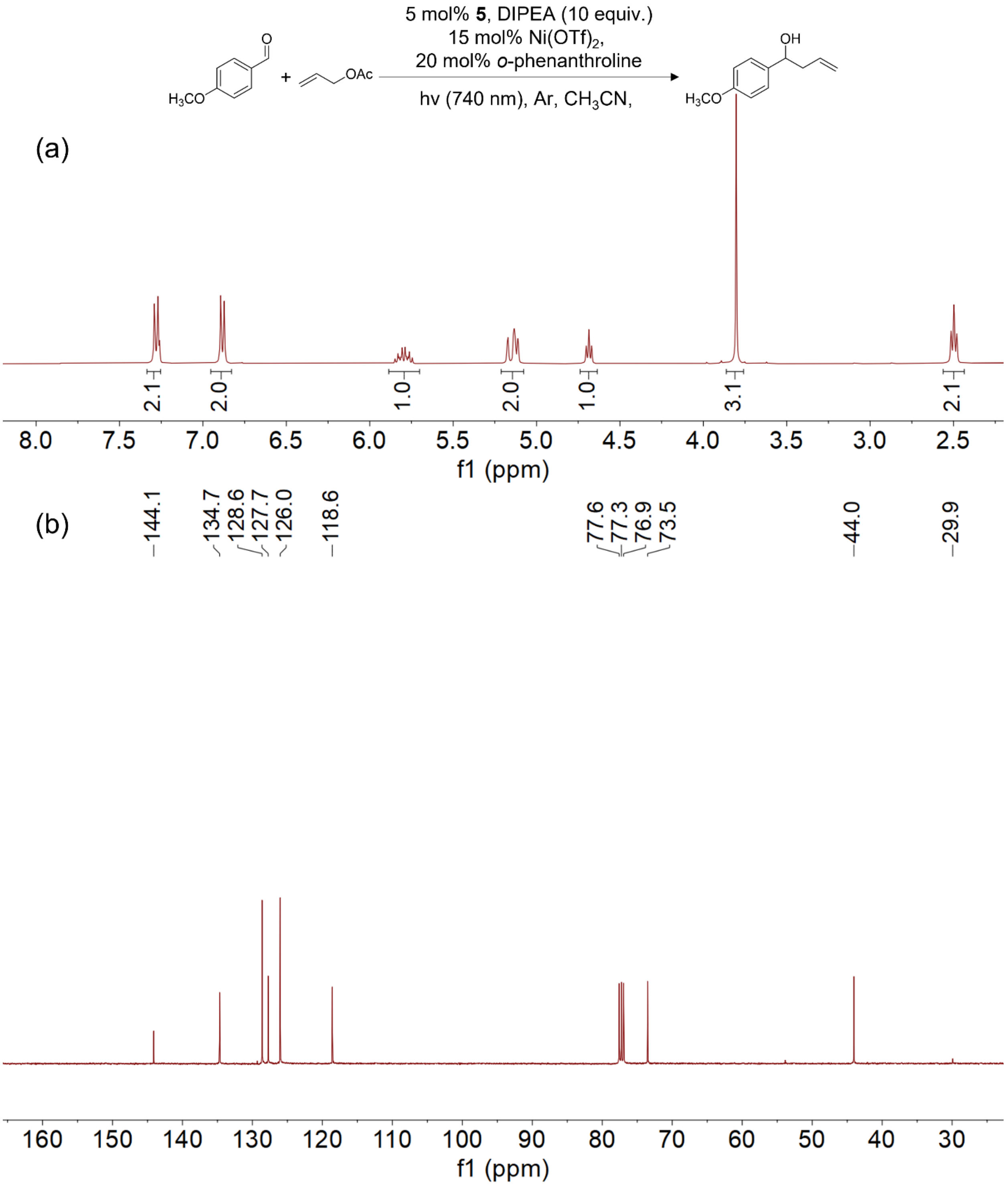

Supplementary Figure 47. ^{1}H NMR (400 MHz, $CDCl_3$, 298 K) (a) and $^{13}C\{^{1}H\}$ NMR (101 MHz, $CDCl_3$, 298 K) (b) spectra after C-C coupling between 4-methoxybenzaldehyde and allyl acetate using **5** as the photosensitizer and $Ni(OTf)_2$ as a cocatalyst. Condition: 50 mM 4-methoxybenzaldehyde, 10 equiv. allyl acetate, 5 mol% **5**, 15 mol% $Ni(OTf)_2$, 20 mol% *o*-phenanthroline, 10 equiv. DIPEA, 2 mL CH_3CN, Ar, 740 nm LEDs, room temperature, 72 h, purified via column chromatography (hexane/ethyl acetate = 10/1).

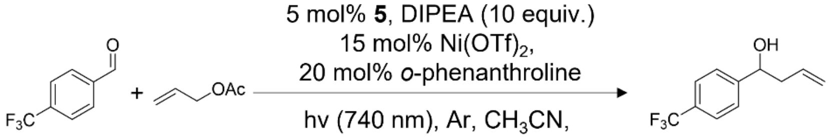

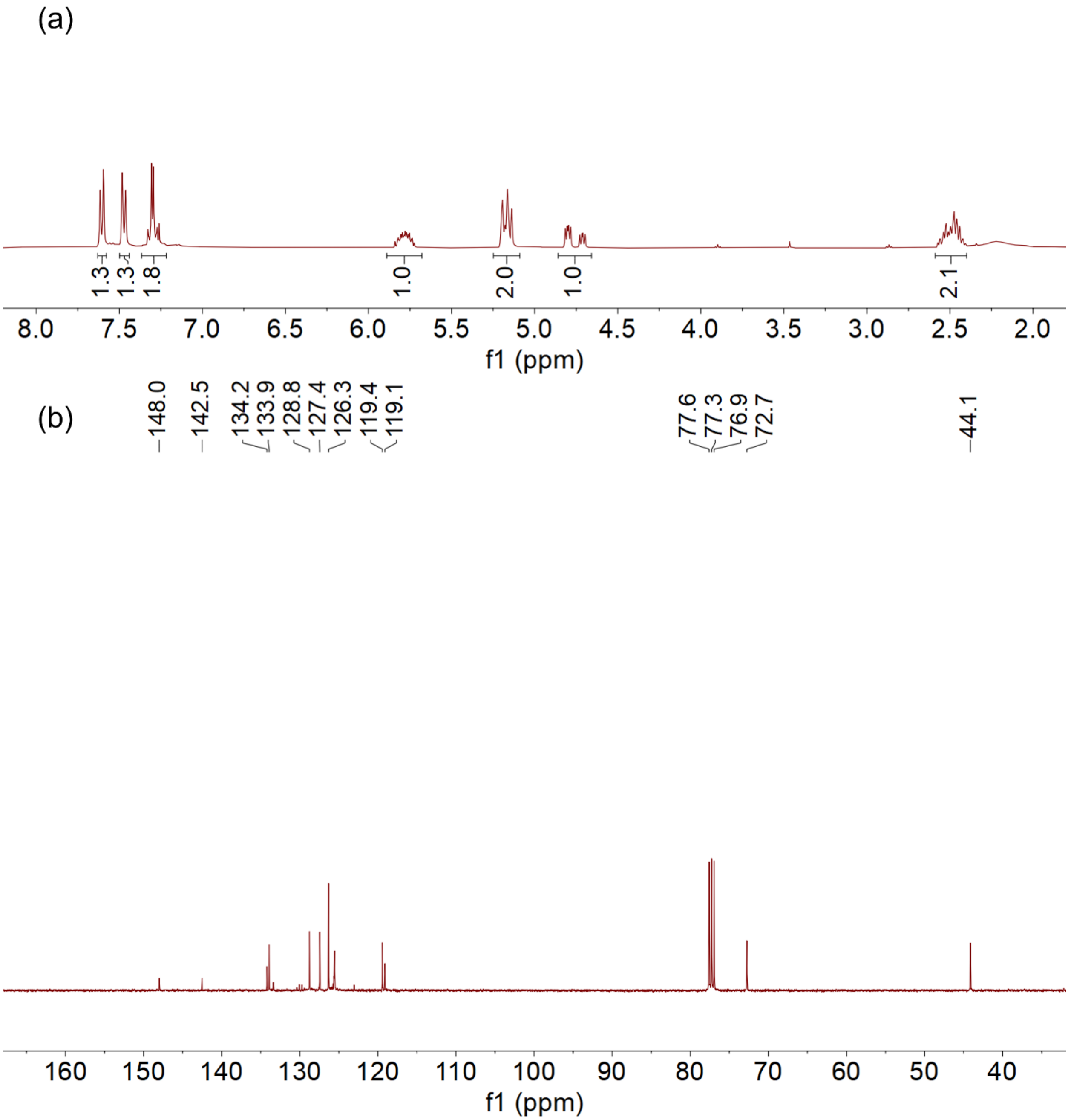

Supplementary Figure 48. ^{1}H NMR (400 MHz, $CDCl_3$, 298 K) (a) and $^{13}C\{^{1}H\}$ NMR (101 MHz, $CDCl_3$, 298 K) (b) spectra after C-C coupling between 4-(trifluoromethyl)benzaldehyde and allyl acetate using **5** as the photosensitizer and $Ni(OTf)_2$ as a cocatalyst. Condition: 50 mM 4-(trifluoromethyl)benzaldehyde, 10 equiv. allyl acetate, 5 mol% **5**, 15 mol% $Ni(OTf)_2$, 20 mol% *o*-phenanthroline, 10 equiv. DIPEA, 2 mL CH_3CN, Ar, 740 LEDs nm, room temperature, 72 h, purified via column chromatography (hexane/ethyl acetate = 10/1).

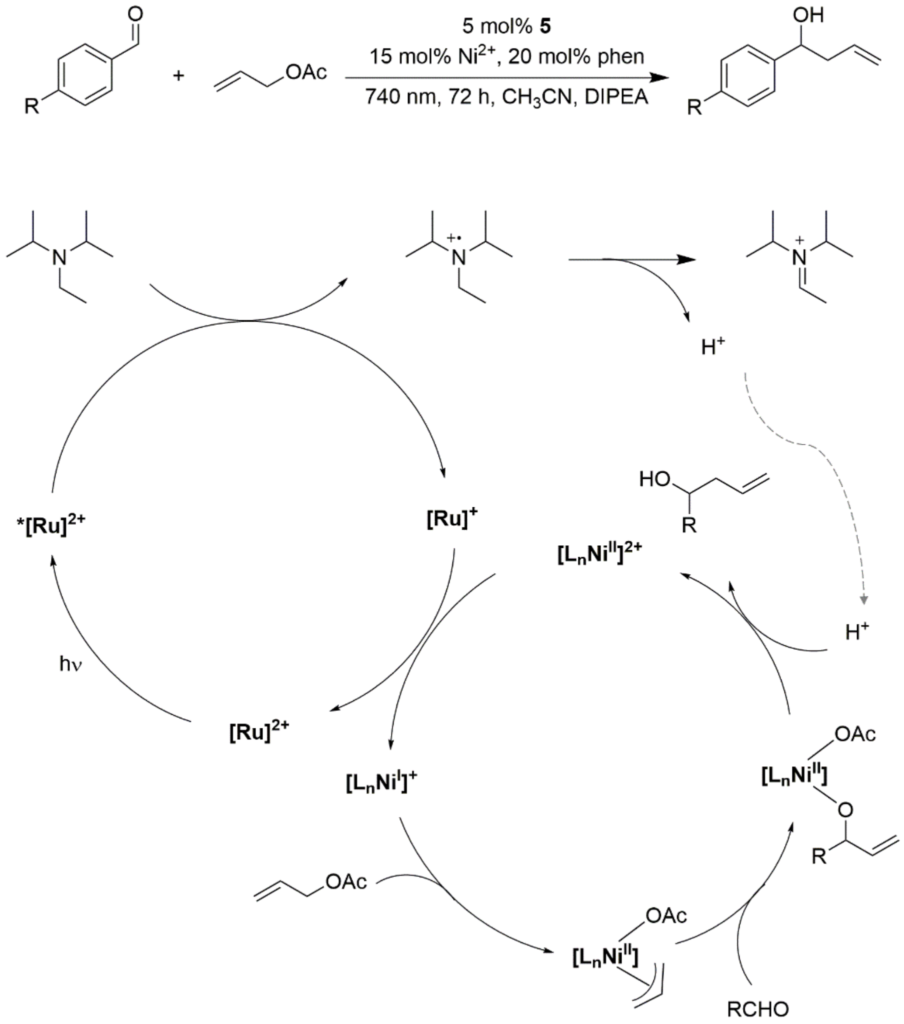

Supplementary Figure 49. Proposed photocatalytic cycle of the allylation of aromatic aldehydes using **5** and Ni-cocatalyst under 740 nm irradiation. **$[Ru]^{2+}$** represents **5**.
